# Age at onset of mental disorders worldwide: large-scale meta-analysis of 192 epidemiological studies

**DOI:** 10.1038/s41380-021-01161-7

**Published:** 2021-06-02

**Authors:** Marco Solmi, Joaquim Radua, Miriam Olivola, Enrico Croce, Livia Soardo, Gonzalo Salazar de Pablo, Jae Il Shin, James B. Kirkbride, Peter Jones, Jae Han Kim, Jong Yeob Kim, Andrè F. Carvalho, Mary V. Seeman, Christoph U. Correll, Paolo Fusar-Poli

**Affiliations:** 1 Neurosciences Department, University of Padua, Padua, Italy; 2 Neuroscience Centre, University of Padua, Padua, Italy; 3 Department of Psychosis Studies, Early Psychosis: Interventions and Clinical-detection (EPIC) Lab, Institute of Psychiatry, Psychology & Neuroscience, King’s College London, London, UK; 4 Imaging Mood- and Anxiety-Related Disorders (IMARD) group, Institut d’Investigacions Biomèdiques August Pi i Sunyer (IDIBAPS), Mental Health Research Networking Center (CIBERSAM), Barcelona, Spain; 5 Department of Clinical Neuroscience, Centre for Psychiatric Research and Education, Karolinska Institutet, Solna, Sweden; 6 Department of Biomedical and Specialty Surgical Sciences, Institute of Psychiatry, University of Ferrara, Ferrara, Italy; 7 Department of Brain and Behavioral Sciences, University of Pavia, Pavia, Italy; 8 Department of Child and Adolescent Psychiatry, Institute of Psychiatry, Psychology & Neuroscience, King’s College London, London, UK; 9 Department of Child and Adolescent Psychiatry, Institute of Psychiatry and Mental Health, Hospital General Universitario Gregorio Marañón School of Medicine, Universidad Complutense, Instituto de Investigación Sanitaria Gregorio Marañón (IiSGM), CIBERSAM, Madrid, Spain; 10 Department of Pediatrics, Yonsei University College of Medicine, Seoul, South Korea; 11 Division of Psychiatry, University College London, London, UK; 12 Department of Psychiatry, University of Cambridge, Cambridge, England; 13 CAMEO Early Intervention Service, Cambridgeshire and Peterborough National Health Service Foundation Trust, Cambridge, England; 14 Yonsei University College of Medicine, Seoul, South Korea; 15 IMPACT (Innovation in Mental and Physical Health and Clinical Treatment) Strategic Research Centre, School of Medicine, Barwon Health, Deakin University, Geelong, VIC, Australia; 16 Department of Psychiatry, University of Toronto, Toronto, ON Canada; 17 Department of Psychiatry, Zucker Hillside Hospital, Glen Oaks, NY USA; 18 Department of Psychiatry and Molecular Medicine, Donald and Barbara Zucker School of Medicine at Hofstra/Northwell, Hempstead, NY USA; 19 Center for Psychiatric Neuroscience, Feinstein Institute for Medical Research, Manhasset, NY USA; 20 Department of Child and Adolescent Psychiatry, Charité-Universitätsmedizin Berlin, Berlin, Germany; 21 OASIS service, South London and Maudsley NHS Foundation Trust, London, UK; 22 National Institute for Health Research, Maudsley Biomedical Research Centre, London, UK

**Keywords:** Psychiatric disorders, Schizophrenia, Bipolar disorder, Depression

## Abstract

Promotion of good mental health, prevention, and early intervention before/at the onset of mental disorders improve outcomes. However, the range and peak ages at onset for mental disorders are not fully established. To provide robust, global epidemiological estimates of age at onset for mental disorders, we conducted a PRISMA/MOOSE-compliant systematic review with meta-analysis of birth cohort/cross-sectional/cohort studies, representative of the general population, reporting age at onset for any ICD/DSM-mental disorders, identified in PubMed/Web of Science (up to 16/05/2020) (PROSPERO:CRD42019143015). Co-primary outcomes were the proportion of individuals with onset of mental disorders before age 14, 18, 25, and peak age at onset, for any mental disorder and across International Classification of Diseases 11 diagnostic blocks. Median age at onset of specific disorders was additionally investigated. Across 192 studies (*n* = 708,561) included, the proportion of individuals with onset of any mental disorders before the ages of 14, 18, 25 were 34.6%, 48.4%, 62.5%, and peak age was 14.5 years (*k* = 14, median = 18, interquartile range (IQR) = 11–34). For diagnostic blocks, the proportion of individuals with onset of disorder before the age of 14, 18, 25 and peak age were as follows: neurodevelopmental disorders: 61.5%, 83.2%, 95.8%, 5.5 years (*k* = 21, median=12, IQR = 7–16), anxiety/fear-related disorders: 38.1%, 51.8%, 73.3%, 5.5 years (*k* = 73, median = 17, IQR = 9–25), obsessive-compulsive/related disorders: 24.6%, 45.1%, 64.0%, 14.5 years (*k* = 20, median = 19, IQR = 14–29), feeding/eating disorders/problems: 15.8%, 48.1%, 82.4%, 15.5 years (*k* = 11, median = 18, IQR = 15–23), conditions specifically associated with stress disorders: 16.9%, 27.6%, 43.1%, 15.5 years (*k* = 16, median = 30, IQR = 17–48), substance use disorders/addictive behaviours: 2.9%, 15.2%, 48.8%, 19.5 years (*k* = 58, median = 25, IQR = 20–41), schizophrenia-spectrum disorders/primary psychotic states: 3%, 12.3%, 47.8%, 20.5 years (*k* = 36, median = 25, IQR = 20–34), personality disorders/related traits: 1.9%, 9.6%, 47.7%, 20.5 years (*k* = 6, median = 25, IQR = 20–33), and mood disorders: 2.5%, 11.5%, 34.5%, 20.5 years (*k* = 79, median = 31, IQR = 21–46). No significant difference emerged by sex, or definition of age of onset. Median age at onset for specific mental disorders mapped on a time continuum, from phobias/separation anxiety/autism spectrum disorder/attention deficit hyperactivity disorder/social anxiety (8-13 years) to anorexia nervosa/bulimia nervosa/obsessive-compulsive/binge eating/cannabis use disorders (17-22 years), followed by schizophrenia, personality, panic and alcohol use disorders (25-27 years), and finally post-traumatic/depressive/generalized anxiety/bipolar/acute and transient psychotic disorders (30-35 years), with overlap among groups and no significant clustering. These results inform the timing of good mental health promotion/preventive/early intervention, updating the current mental health system structured around a child/adult service schism at age 18.

## Introduction

Individuals with mental disorders have a decreased life expectancy of 10–15 years in comparison with the general population [1–4]. Early interventions at the first onset of mental disorders can improve several outcomes [5, 6]. Primary indicated prevention in those at clinical high risk has the potential to alter the course of the disorder and improve outcomes [7–9]. For example, young people with attenuated symptoms for psychosis [10–13] and functional impairments accumulate several risk factors and have a 25% probability of developing the disorder over 3 years [14]. Clinical care for these individuals is typically implemented in specialised clinical services [15–18] and has the potential to delay or impede the transition to psychosis, although the efficacy of preventive interventions awaits more robust evidence [19–21]. Targeted preventive approaches involve screening programmes in asymptomatic individuals who have significant risk factors for certain psychiatric disorders [7, 22, 23] (primary selective prevention [7, 8]) or public health campaigns in the general population (primary universal prevention) [7, 8, 24]. To date, these initiatives have been mostly piloted for young people with emerging severe mental disorders [8]. A further complementary approach is to promote good mental health, as opposed to preventing mental disorders [7, 25, 26].

Although promotion of good mental health, prevention and early intervention can be implemented over the lifespan, the benefits are maximal when young people are targeted at around the time of onset of mental disorders. Unfortunately, the peak ages and ranges at onset for mental disorders are not fully established, with conflicting findings across [27, 28] and within studies [29], partly due to methodological limitations, including selection biases in recruitment for clinical studies [30]. General population-level studies (birth cohort, cross-sectional or incidence studies) provide the most robust onset age estimates [30]. However, to date, no comprehensive epidemiologically sound, large-scale meta-analysis has pooled data from these population-based studies that are representative of the general population to estimate the peak age at the onset across the globe and the proportion of individuals with mental disorders at specific age points. This study’s goal was to fill this gap aiming to optimise timely intervention, prevention and promotion of good mental health opportunities at the time of onset of mental disorders.

## Method

### Search strategy

A study protocol was registered and is publicly available on PROSPERO (CRD42019143015). We performed a systematic review adhering to the preferred reporting items for systematic reviews and meta-analyses (PRISMA) recommendations [31] (e-Table 1) and the meta-analysis of observational studies in epidemiology (MOOSE) guidelines (e-Table 2) [32].

At least two authors (MO, ER, LS, MS, all MDs) independently searched PubMed and Web of Science using the following terms: “age at onset” (topic) and “mental disorder” (topic), plus “birth cohort” (topic) and “mental disorder” (topic), plus “incidence” (topic) and “mental disorder” (topic), without restriction to the type of mental disorder. Additionally, reviews and reference lists of included studies were manually searched. The literature was searched from database inception until 16/05/2020. Hits were first screened at the title/abstract level, then full texts of the remaining articles were assessed, recording reasons for exclusion (as per the criteria noted below).

### Inclusion and exclusion criteria

Included were: (i) original birth cohort or cross-sectional studies retrospectively assessing age at disorder onset, or prospective incidence studies [30], (ii) focusing on the general population, (iii) assessing age at onset of mental disorders, defined according to international classification diseases (ICD) or diagnostic and statistical manual (DSM)-any version criteria, or by established psychometric instruments with validated cut-offs that defined ICD/DSM diagnostic categories, (iv) written in English.

Excluded were: (i) studies sampling clinical groups that were not representative of the general population, (ii) studies assessing the prevalence, not onset age of mental disorders, (iii) reviews, meta-analyses, case reports or other non-original studies, (iv) non-English language articles.

### Data extraction

The following variables were extracted into pre-defined excel spreadsheets: DOI/PMID, author, publication year, country of study, study design (birth cohort, cross-sectional, incidence studies, all representative of the general population), name of the cohort (if available), ICD/DSM diagnostic criteria, onset definition (first symptoms, first diagnosis, first hospitalisation), a specific type of mental disorder, sample age range, number of participants, number of cases developing incident mental disorders. Age at onset of mental disorders was extracted as available in each of the included studies (see statistics). Data extraction was performed independently by the same pairs of authors who performed the literature screening.

### Study quality assessment

To the best of our knowledge, no quality assessment measure has been validated for the type of studies included in the current meta-analysis. Therefore, the risk of bias was evaluated with an ad-hoc list of criteria derived from the Newcastle-Ottawa scale (NOS) [33], which included the definition of onset age of the mental disorder, diagnostic criteria employed, and study design. However, since these criteria were not validated, they were not employed to categorise studies according to their quality; they were only used for descriptive reporting.

### Statistical analysis

Study-defined age at onset of mental disorders encompassed: (i) mean and standard deviation (SD), percentiles, median and/or interquartile range (IQR) of age at disorder onset; (ii) proportion of cases of a sample whose age at disorder onset fell into a certain age group, (iii) number of incident cases developing mental disorders, separately for age groups (e.g., for national registries).

We defined co-primary outcomes as the proportion of individuals with age at disorder onset of any and specific mental disorder groups before 14, 18 and 25 years old, and peak age at onset for any mental disorder and for each diagnostic group. Diagnostic groups matched all 19 ICD-11 diagnostic blocks under “Mental, behavioural or neurodevelopmental disorder”, namely neurodevelopmental disorders, schizophrenia-spectrum and primary psychotic disorders, catatonia, mood disorders, anxiety and fear-related disorders, obsessive-compulsive or related disorders, disorders specifically associated with stress, dissociative disorders, feeding or eating disorders, elimination disorders, disorders of bodily experience, disorders due to substance use or addictive behaviour, impulse-control disorders, disruptive behaviour or dissocial disorders, personality disorders and related traits, paraphilic disorders, factitious disorders, neurocognitive disorders, disorders associated with pregnancy childbirth or puerperium. Individual studies adopted different age subgroupings and age ranges. To our knowledge, there is no standard method to pool such varying descriptive statistics of the distribution of a variable (age at onset of mental disorders) when the variable of interest follows a non-normal distribution and/or is heterogeneously censored. Therefore, we have developed an ad-hoc method to meta-analyse these estimates. To assess these co-primary outcomes, we first estimated the histogram of the age at disorder onset that minimised the sum of squared errors (SSE) of the study-reported data. From this histogram, we then derived peak age at onset, as well as the proportion of individuals showing an onset before 14, 18 and 25 years of age, and the 25%, 50% and 75% percentiles of the age at disorder onset. Thereafter, we used an empirical bootstrap approach [34] to estimate the 95% confidence intervals of age at onset.

Additional sensitivity analyses were performed. First, we repeated the analyses for specific mental disorders, pre-selected on the basis of their epidemiological and clinical relevance: attention-deficit/hyperactivity disorder (ADHD), autism spectrum disorder (ASD), generalised anxiety disorder (GAD), panic disorder, separation anxiety disorder, specific phobia, social anxiety disorder, obsessive-compulsive disorder (OCD), disorders due to use of alcohol, disorders due to use of cannabis, anorexia nervosa, bulimia nervosa, binge eating disorder, depressive disorders, bipolar or related disorders (bipolar disorder), acute and transient psychotic disorders (ATPD), schizophrenia, and post-traumatic stress disorder (PTSD). Second, we repeated analyses for any disorder, stratifying by onset definition (i.e., first symptoms, first diagnosis, first hospitalisation). Third, we repeated the analyses for any disorder stratifying by sex. Finally, we compared the median age at onset between each pair of specific mental disorders, counting the proportion of bootstrapped randomisations in which the median age at onset of one disorder was larger than the median age of the other disorder (alpha = 0.05, two-sided).

For all analyses, we required ≥2 studies to estimate the histogram and to apply the bootstrap procedure. For full details regarding the statistical analysis, see e-methods.

## Results

### Literature search and database

We identified 5,442 possible publications after removing duplicates, of which 4,516 were excluded after title/abstract screening. An additional 734 publications were excluded after full-text review (see e-Fig. 1 and supplementary material—e-Table 3—for full details). For characteristics of the 192 included studies (birth cohort studies = 15, cross-sectional studies = 150, incidence studies = 27), see e-Table 4. These studies were comprised of data from 708,561 individuals, all of whom were diagnosed with a mental disorder. Overall, 54 studies were set in U.S., 23 studies in multiple countries, 11 in Australia, ten in Finland, eight in Germany, six each in Canada and the Netherlands, five in China, Denmark, South Africa, Spain, United Kingdom, four in Israel, South Korea, Sweden, three in Ethiopia, Mexico, New Zealand, Nigeria, Switzerland, Taiwan, two in France, Iraq, Singapore, and one study each in additional countries.

**Fig. 1:**
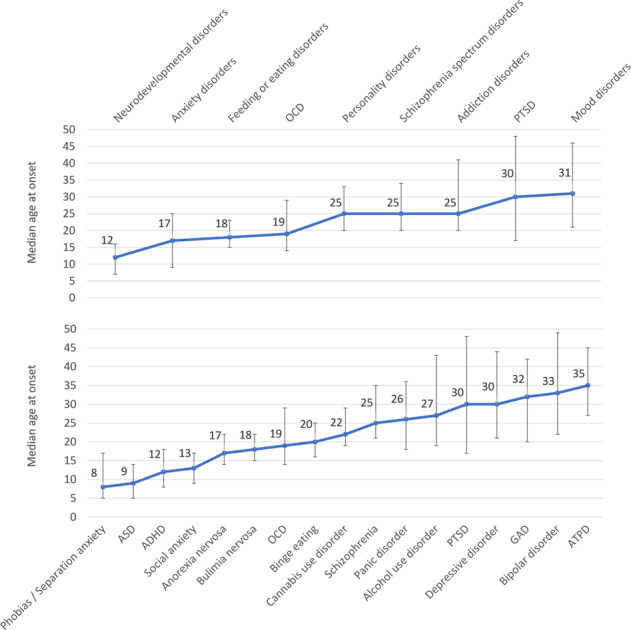
Meta-analytic median age at onset of mental disorders. The line indicates the median age at onset of mental disorders (ICD-11 diagnostic blocks or spectra above, specific mental disorders below), the bar indicates the 25th and 75th percentiles. ICD-11 blocks of mental disorders. Addiction disorders: disorders due to substance use or addictive behaviour, Anxiety and fear: anxiety and fear-related disorders, OCD related: obsessive-compulsive or related disorders, Schizophrenia spectrum disorders: schizophrenia-spectrum and primary psychotic disorders. ICD-11 specific mental disorders. ADHD attention deficit hyperactivity disorder, ASD: autism spectrum disorder, ATPD, acute and transient psychotic disorder; Binge eating: binge eating disorder, Bipolar disorder: bipolar or related disorders, GAD generalised anxiety disorder, OCD obsessive-compulsive disorder, Phobia: specific phobia, PTSD post-traumatic stress disorder, Separation anxiety: separation anxiety disorder, Social anxiety: social anxiety disorder.

There were insufficient studies for inclusion on catatonia, dissociative disorders, elimination disorders, disorders of bodily experience, impulse-control disorders, disruptive behaviour or dissocial disorders, paraphilic disorders, factitious disorders, neurocognitive disorders, and disorders associated with pregnancy childbirth/puerperium.

### Global age at the onset across diagnostic spectra of mental disorders

The proportion of individuals with age at onset before 14, 18 and 25 years of age and peak age at onset for diagnostic spectra (co-primary outcome measures) are reported in Table 1. Overall, before age 14, 18, and 25 years, a disorder had already emerged in 34.6%, 48.4%, and 62.5% of individuals (Table 1).

**Table 1:** Meta-analytic epidemiological estimates of age at onset of mental disorder (ICD-11 blocks highlighted in italics) in the general population.

	No of Samples	Peak age at onset (years)	Proportion onset by 14 years	Proportion onset by 18 years	Proportion onset by 25 years	p25	Median	p75
Any mental disorder	14	14.5	34.6%	48.4%	62.5%	11	18	34
*Neurodevelopmental disorders*	*21*	*5.5*	*61.5%*	*83.2%*	*95.8%*	*7*	*12*	*16*
Autism spectrum disorder	2	5.5	72.4%	89.8%	94.8%	5	9	14
Attention deficit hyperactivity disorder	12	9.5	56.8%	73.0%	91.8%	8	12	18
*Anxiety and fear-related disorders*	*73*	*5.5*	*38.1%*	*51.8%*	*73.3%*	*9*	*17*	*25*
Specific phobia/separation anxiety disorder	22	5.5	72.4%	75.0%	80.4%	5	8	17
Social anxiety disorder	42	14.5	50.9%	79.1%	87.5%	9	13	17
Panic disorder	22	15.5	8.2%	22.5%	45.7%	18	26	36
Generalised anxiety disorder	24	15.5	8.6%	20.4%	33.0%	20	32	42
*Obsessive-compulsive related disorders*	*20*	*14.5*	*24.6%*	*45.1%*	*64.0%*	*14*	*19*	*29*
Obsessive-compulsive disorder	20	14.5	24.6%	45.1%	64.0%	14	19	29
*Feeding or eating disorders*	*11*	*15.5*	*15.8%*	*48.1%*	*82.4%*	*15*	*18*	*23*
Anorexia nervosa	8	15.5	18.2%	55.2%	78.7%	14	17	22
Bulimia Nervosa	8	15.5	16.0%	45.3%	82.9%	15	18	22
Binge eating disorder	5	19.5	12.3%	34.5%	73.5%	16	20	25
*Trauma-related disorders*	*16*	*15.5*	*16.9%*	*27.6%*	*43.1%*	*17*	*30*	*48*
Post-traumatic stress disorder	16	15.5	16.9%	27.6%	43.1%	17	30	48
*Disorders due to substance use or addictive behaviours*	*58*	*19.5*	*2.9%*	*15.2%*	*48.8%*	*20*	*25*	*41*
Disorder due to use of cannabis	10	19.5	3.2%	17.5%	64.6%	19	22	29
Disorder due to use of alcohol	44	19.5	4.2%	18.3%	44.8%	19	27	43
*Schizophrenia-spectrum and primary psychotic disorders*	*36*	*20.5*	*3.0%*	*12.3%*	*47.8%*	*20*	*25*	*34*
Schizophrenia	25	20.5	2.0%	8.2%	47.4%	21	25	35
Acute and transient psychotic disorder	2	18.5	1.8%	6.6%	20.6%	27	35	45
*Personality disorders*	*6*	*20.5*	*1.9%*	*9.6%*	*47.7%*	*20*	*25*	*33*
*Mood disorders*	*79*	*20.5*	*2.5%*	*11.5%*	*34.5%*	*21*	*31*	*46*
Depressive disorders	62	19.5	3.1%	13.2%	36.9%	21	30	44
Bipolar or related disorders	40	19.5	5.1%	13.7%	32.0%	22	33	49

p25, p75, 25th and 75th percentile. Only the earliest peaks are shown. Different metric on the same sample has been extracted from multiple studies.

Corresponding figures were, respectively, for neurodevelopmental disorders: 61.5%, 83.2%, 95.8%, for anxiety/fear-related disorders: 38.1%, 51.8%, 73.3%, for obsessive-compulsive/related disorders: 24.6%, 45.1%, 64.0%, for feeding/eating disorders: 15.8%, 48.1%, 82.4%, for disorders specifically associated with stress: 16.9%, 27.6%, 43.1%, for substance use/addictive behaviour disorders: 2.9%, 15.2%, 48.8%, and for schizophrenia-spectrum/primary psychotic disorders: 3%, 12.3%, 47.8%, for personality disorders/traits: 1.9%, 9.6%, 47.7%, and for mood disorders: 2.5%, 11.5%, 34.5%.

**Fig. 2:**
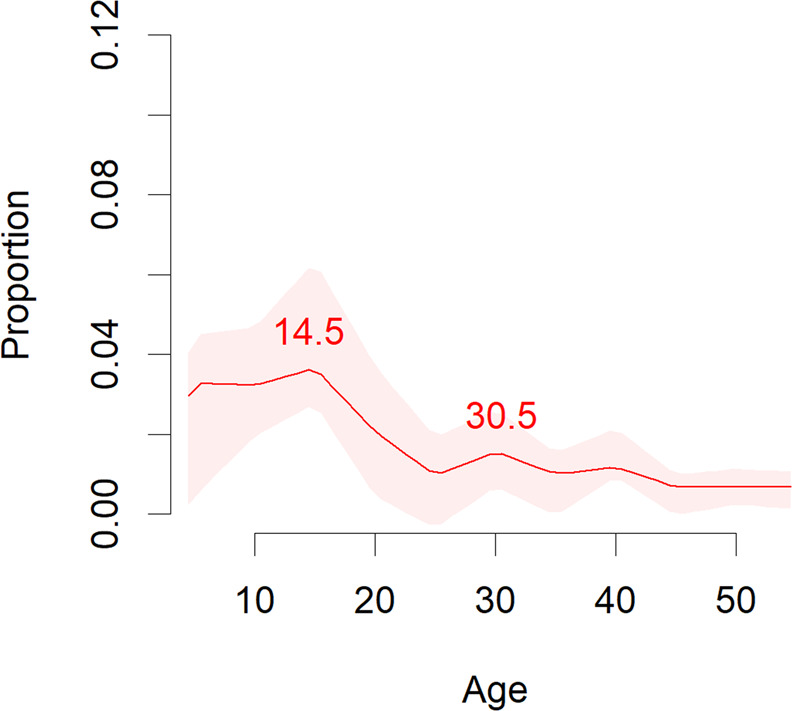
Meta-analytic distribution of age of onset for any mental disorders. Meta-analytic epidemiological proportion (*y*-axis) and peak age at onset (red line) for any mental disorders in the general population, with 95%CIs (pink shadows).

**Fig. 3:**
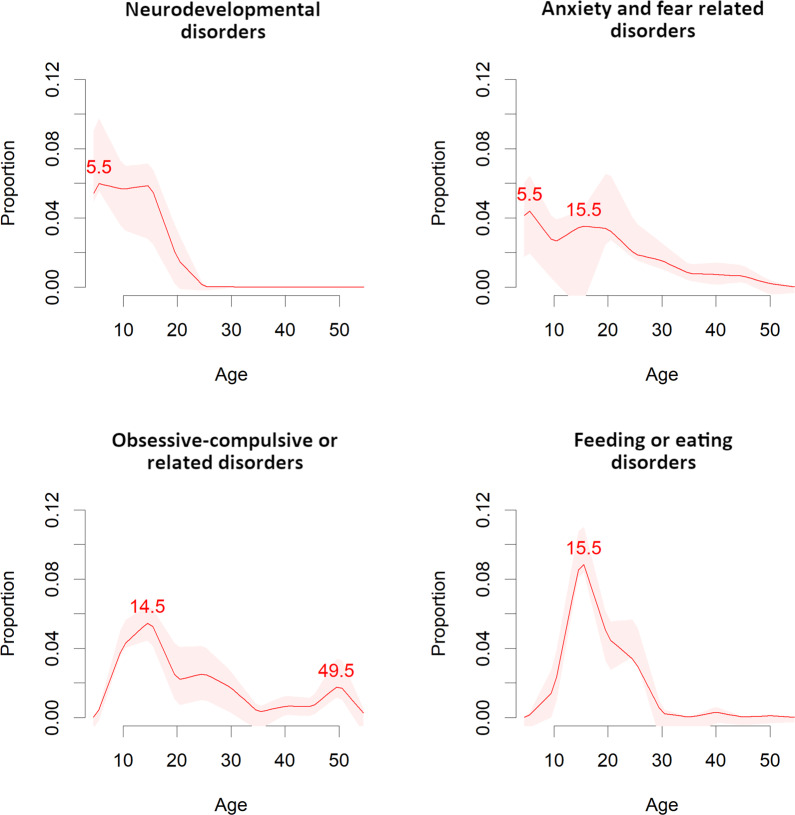
Meta-analytic distribution of age of onset for specific mental disorders blocks. Meta-analytic epidemiological proportion (*y*-axis) and peak age at onset (red line) for neurodevelopmental, anxiety and fear-related, obsessive-compulsive related, and feeding or eating disorders (ICD-11 blocks) in the general population, with 95%CIs (pink shadows).

**Fig. 4:**
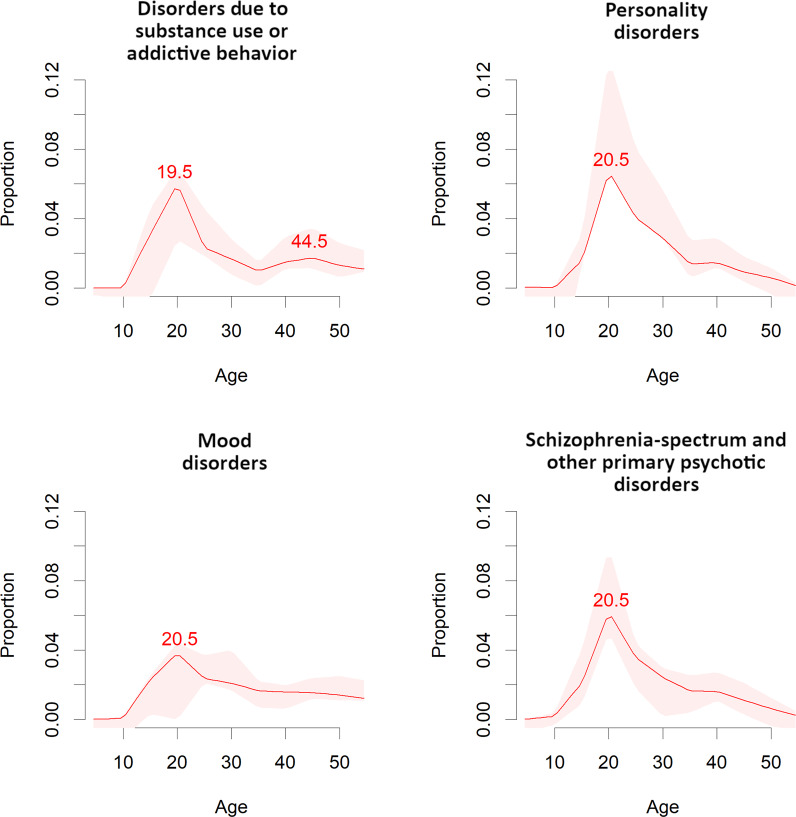
Meta-analytic distribution of age of onset for specific mental disorders blocks. Meta-analytic epidemiological proportion (*y*-axis) and peak age at onset (red line) for disorders due to substance use or addictive behaviour, personality, mood, and schizophrenia-spectrum and primary psychotic disorders (ICD-11 blocks) in the general population, with 95%CIs (pink shadows).

Curves representing the median, 25th, and 75th percentiles and peak age at onset for mental disorder spectra are reported in Figs. 1–4. The peak and median age at onset for any mental disorder were 14.5 years and 18 years. The earliest age peaks of disorder onset were: neurodevelopmental disorders (peak = 5.5/median = 12 years), anxiety/fear-related disorders (peak = 5.5/median = 17 years), obsessive-compulsive/related disorders (peak = 14.5/median = 18 years), feeding/eating disorders (peak = 15.5/median = 18 years), disorders specifically associated with stress (peak = 15.5/median = 30 years), substance use/addictive behaviour disorders (peak = 19.5/median = 25 years), schizophrenia-spectrum/primary psychotic and personality disorders/traits (peak = 20.5/median = 25 years), and mood disorders (peak = 20.5/median = 31 years). Second age peaks emerged for any mental disorder at (peak = 30.5 years), anxiety/fear-related disorders (peak = 15.5 years), obsessive-compulsive/related disorders (peak = 49.5 years), disorders specifically associated with stress (peaks = 30.5 and 49.5 years), and substance use/addictive behaviour disorders (peak = 44.5 years).

### Sensitivity analyses

The proportion of individuals with age at the onset before 14, 18 and 25 years of age for specific mental disorders are reported in Table 1. Overall, the proportion of patients developing disorders before age 25 is more than nine out of ten for ASD and ADHD; nine out of ten for social anxiety disorder; around eight out of ten for specific phobia/separation anxiety disorders and anorexia nervosa; seven to eight out of ten for binge eating disorders; six to seven out of ten for OCD and disorders due to use of cannabis; four to five out of ten for disorders due to use of alcohol, schizophrenia, PTSD, panic disorder, and personality disorders; three to four out of ten for GAD, bipolar/related disorders, and depressive disorders; and two out of ten for ATPD. The curves representing the peak age at onset for specific mental disorders are presented in e-Figs. 2–17.

As reported in Table 2, the age at onset was mostly similar between males and females, although there was a trend towards younger ages for males in disorders due to substance use or addictive behaviours (median = 4 years earlier), mood disorders (median = 2 years earlier) or schizophrenia-spectrum and primary psychotic disorders (median = 1 year earlier). Similarly, there were little differences in age at onset among studies where onset was defined according to first symptoms, first diagnosis or first hospitalisation, although there was a trend towards younger ages for first symptoms than for the first diagnosis in disorders due to substance use/addictive behaviours (median symptoms 9 years earlier), mood disorders (median 8 years earlier), anxiety and fear-related disorders (median 3 years earlier). For schizophrenia-spectrum and primary psychotic disorders, we observed a trend from symptoms to hospital admission (median a year later) to diagnosis (median another year later). There were other minor differences in disorders with fewer (<10) separate studies for sex or onset definition, for which we suggest caution as the estimations may be less accurate than others.

**Table 2:** Age at onset by onset definition and by sex for mental disorders (ICD-11 blocks highlighted in italics).

Disorder	Onset definition/sex	Samples	Peak age at onset (years)	Proportion onset by 14 years	Proportion onset by 18 years	Proportion onset by 25 years	p25	Median	p75
Any mental disorder	First symptoms	5	4.5	41.5%	50.0%	69.5%	8	18	27
Any mental disorder	First diagnosis	6	5.5	38.6%	54.1%	68.3%	9	16	33
*Neurodevelopmental disorders*	*First symptoms*	*8*	*5.5*	*40.4%*	*56.0%*	*62.9%*	*7*	*16*	*–*
*Neurodevelopmental disorders*	*First diagnosis*	*11*	*5.5*	*61.3%*	*82.6%*	*95.0%*	*7*	*12*	*16*
*Neurodevelopmental disorders*	*Female*	*5*	*9.5*	*59.6%*	*74.5%*	*82.2%*	*7*	*12*	*18*
*Neurodevelopmental disorders*	*Male*	*4*	*9.5*	*67.0%*	*81.2%*	*89.0%*	*7*	*11*	*15*
Autism spectrum disorder	First diagnosis	2	5.5	72.4%	89.8%	94.8%	5	9	14
Autism spectrum disorder	Female	2	5.5	80.1%	92.3%	96.0%	5	8	12
Autism spectrum disorder	Male	2	5.5	80.6%	92.4%	96.0%	5	8	12
Attention deficit hyperactivity disorder	First symptoms	2	4.5	99.8%	99.8%	100.0%	4	5	7
Attention deficit hyperactivity disorder	First diagnosis	9	9.5	56.2%	72.5%	91.8%	8	12	18
Attention deficit hyperactivity disorder	Female	3	9.5	65.0%	81.0%	95.2%	8	11	16
Attention deficit hyperactivity disorder	Male	3	9.5	67.5%	81.3%	94.9%	8	11	15
*Anxiety and fear-related disorders*	*First symptoms*	*31*	*4.5*	*45.1%*	*59.9%*	*71.9%*	*5*	*15*	*27*
*Anxiety and fear-related disorders*	*First diagnosis*	*18*	*5.5*	*35.5%*	*48.5%*	*71.0%*	*9*	*18*	*26*
*Anxiety and fear-related disorders*	*Female*	*6*	*19.5*	*11.6%*	*22.9%*	*45.8%*	*18*	*26*	*40*
*Anxiety and fear-related disorders*	*Male*	*7*	*19.5*	*21.8%*	*31.4%*	*52.4%*	*15*	*24*	*37*
Specific phobia/separation anxiety disorder	First symptoms	12	4.5	71.2%	77.6%	82.8%	5	7	16
Specific phobia/separation anxiety disorder	First diagnosis	9	5.5	72.1%	76.0%	85.0%	6	9	16
Specific phobia/separation anxiety disorder	Female	2	4.5	58.3%	60.6%	68.2%	5	8	30
Specific phobia/separation anxiety disorder	Male	2	5.5	50.0%	53.6%	70.2%	7	13	26
Social anxiety disorder	First symptoms	9	14.5	54.6%	75.4%	81.5%	7	13	17
Social anxiety disorder	First diagnosis	7	14.5	47.5%	77.1%	85.4%	10	14	17
Panic disorder	First symptoms	10	15.5	11.9%	28.9%	56.4%	17	22	32
Panic disorder	First diagnosis	10	39.5	9.3%	20.7%	43.8%	19	27	40
Panic disorder	Female	3	20.5	20.8%	29.0%	53.4%	16	24	31
Panic disorder	Male	3	15.5	16.1%	32.9%	56.9%	16	22	31
Generalised anxiety disorder	First symptoms	13	15.5	9.4%	21.3%	32.8%	19	39	52
Generalised anxiety disorder	First diagnosis	8	45.5	7.5%	15.2%	27.3%	23	34	48
*Obsessive-compulsive related disorder*	*First symptoms*	*6*	*14.5*	*23.4%*	*48.1%*	*71.6%*	*14*	*18*	*25*
*Obsessive-compulsive related disorder*	*First diagnosis*	*11*	*14.5*	*24.1%*	*44.1%*	*56.4%*	*14*	*20*	*35*
*Obsessive-compulsive related disorder*	*Female*	*4*	*15.5*	*16.9%*	*32.8%*	*55.5%*	*16*	*23*	*30*
*Obsessive-compulsive related disorder*	*Male*	*4*	*10.5*	*27.7%*	*42.6%*	*67.1%*	*13*	*20*	*27*
*Feeding or eating disorders*	*First symptoms*	*4*	*15.5*	*10.2%*	*40.1%*	*77.2%*	*16*	*19*	*24*
*Feeding or eating disorders*	*First diagnosis*	*7*	*15.5*	*16.0%*	*44.7%*	*74.2%*	*15*	*18*	*25*
*Feeding or eating disorders*	*Female*	*4*	*14.5*	*23.0%*	*54.8%*	*76.5%*	*14*	*17*	*23*
*Feeding or eating disorders*	*Male*	*4*	*14.5*	*37.6%*	*67.3%*	*86.9%*	*12*	*15*	*19*
Anorexia nervosa	First symptoms	3	15.5	20.4%	59.9%	89.0%	14	16	20
Anorexia nervosa	First diagnosis	5	15.5	10.6%	37.3%	54.6%	16	22	87
Binge eating disorder	First symptoms	2	19.5	15.5%	31.4%	70.4%	16	20	28
Binge eating disorder	First diagnosis	3	15.5	8.2%	34.5%	73.5%	16	20	25
*Trauma-related disorders*	*First symptoms*	*6*	*25.5*	*13.3%*	*23.4%*	*33.0%*	*18*	*33*	*48*
*Trauma-related disorders*	*First diagnosis*	*7*	*15.5*	*19.4%*	*31.3%*	*46.1%*	*15*	*29*	*46*
*Disorders due to substance use or addictive behaviours*	*First symptoms*	*34*	*15.5*	*8.2%*	*39.1%*	*78.4%*	*16*	*19*	*23*
*Disorders due to substance use or addictive behaviours*	*First diagnosis*	*21*	*20.5*	*1.6%*	*9.5%*	*41.3%*	*21*	*28*	*43*
*Disorders due to substance use or addictive behaviours*	*Female*	*11*	*20.5*	*0.8%*	*5.7%*	*34.3%*	*22*	*31*	*45*
*Disorders due to substance use or addictive behaviours*	*Male*	*12*	*20.5*	*2.5%*	*13.2%*	*41.8%*	*20*	*27*	*41*
Disorder due to use of cannabis	First symptoms	2	20.5	5.8%	8.5%	17.2%	30	44	63
Disorder due to use of cannabis	First diagnosis	5	20.5	3.0%	16.7%	66.0%	19	22	27
Disorder due to use of cannabis	Female	3	20.5	4.0%	15.9%	61.3%	19	23	28
Disorder due to use of cannabis	Male	3	20.5	3.3%	14.6%	61.5%	19	23	28
Disorder due to use of alcohol	First symptoms	25	15.5	8.4%	39.2%	71.9%	16	19	26
Disorder due to use of alcohol	First diagnosis	15	20.5	0.9%	4.5%	27.1%	24	36	47
Disorder due to use of alcohol	Female	9	44.5	0.1%	1.5%	16.2%	29	40	50
Disorder due to use of alcohol	Male	9	15.5	2.9%	13.8%	29.0%	22	36	47
*Schizophrenia-spectrum and primary psychotic disorders*	*First symptoms*	*11*	*24.5*	*6.0%*	*15.0%*	*51.1%*	*20*	*24*	*29*
*Schizophrenia-spectrum and primary psychotic disorders*	*First diagnosis*	*10*	*20.5*	*2.1%*	*11.2%*	*45.3%*	*20*	*26*	*39*
*Schizophrenia-spectrum and primary psychotic disorders*	*First hospitalisation*	*14*	*24.5*	*12.9%*	*17.8%*	*45.4%*	*20*	*25*	*32*
*Schizophrenia-spectrum and primary psychotic disorders*	*Female*	*18*	*20.5*	*2.4%*	*10.0%*	*45.4%*	*21*	*26*	*35*
*Schizophrenia-spectrum and primary psychotic disorders*	*Male*	*18*	*20.5*	*7.6%*	*15.9%*	*49.9%*	*20*	*25*	*32*
Schizophrenia	First symptoms	8	24.5	7.2%	18.0%	54.8%	19	24	28
Schizophrenia	First diagnosis	8	20.5	0.9%	6.5%	43.7%	21	26	39
Schizophrenia	First hospitalisation	10	25.5	30.2%	34.5%	52.0%	11	24	31
Schizophrenia	Female	16	20.5	2.5%	8.3%	45.7%	21	25	33
Schizophrenia	Male	16	20.5	6.0%	11.6%	48.7%	20	25	31
Acute and transient psychotic disorder	First diagnosis	2	19.5	1.8%	6.6%	20.6%	27	35	45
*Personality disorders*	*First symptoms*	*2*	*5.5*	*93.6%*	*96.3%*	*96.5%*	*5*	*7*	*10*
*Personality disorders*	*First diagnosis*	*4*	*20.5*	*2.2%*	*10.0%*	*49.2%*	*20*	*25*	*32*
*Personality disorders*	*Female*	*3*	*20.5*	*0.8%*	*5.5%*	*37.7%*	*22*	*27*	*37*
*Personality disorders*	*Male*	*3*	*20.5*	*14.4%*	*19.6%*	*47.7%*	*19*	*25*	*34*
*Mood disorders*	*First symptoms*	*32*	*19.5*	*12.2%*	*26.1%*	*51.7%*	*17*	*24*	*36*
*Mood disorders*	*First diagnosis*	*40*	*20.5*	*2.2%*	*10.7%*	*33.3%*	*22*	*32*	*48*
*Mood disorders*	*Female*	*22*	*20.5*	*1.0%*	*5.4%*	*22.0%*	*26*	*40*	*56*
*Mood disorders*	*Male*	*22*	*20.5*	*3.4%*	*9.0%*	*24.9%*	*25*	*38*	*55*
Depressive disorder	First symptoms	22	15.5	10.2%	25.9%	47.0%	17	26	34
Depressive disorder	First diagnosis	35	19.5	2.5%	12.0%	35.8%	21	31	46
Depressive disorder	Female	18	20.5	1.1%	5.9%	22.4%	26	40	55
Depressive disorder	Male	17	20.5	3.5%	9.0%	24.5%	25	38	54
A bipolar or related disorder	First symptoms	15	19.5	10.3%	30.9%	68.8%	16	21	26
A bipolar or related disorder	First diagnosis	24	20.5	0.6%	2.7%	18.8%	28	40	52
A bipolar or related disorder	Female	8	19.5	1.7%	7.8%	23.3%	26	39	52
A bipolar or related disorder	Male	7	49.5	4.4%	12.2%	24.5%	25	39	52

p25, p75, 25th and 75th percentile. Only the earliest peaks are shown. Curves were calculated when at least two studies were included for each ICD-11 block or disorder. Bootstrap comparisons were run when at least five studies were included for each disorder; no significant difference emerged by sex or onset definition.

Despite some differences shown in e-Table 5, the median age at onset of specific mental disorders maps on a continuum, with no clear clustering across different disorders. Descriptively, the earliest median age of onset was observed for phobias/separation anxiety, ASD, ADHD, social anxiety disorders (8-13 years), followed by anorexia nervosa, bulimia nervosa, OCD, binge eating, cannabis use disorders (17-22 years), later schizophrenia, personality and alcohol use disorders (25-27 years), and, finally, PTSD, depressive disorder, GAD, bipolar disorder, and ATPD (30-35 years).

## Discussion

This is the first large-scale epidemiological meta-analysis that includes all available general population birth, cross-sectional and incidence studies investigating the age at onset of any ICD/DSM-mental disorders. Overall, the global onset of the first mental disorder occurs before age 14 in one-third of individuals, age 18 in almost half (48.4%), and before age 25 in half (62.5%), with a peak/median age at onset of 14.5/18 years across all mental disorders. However, there was significant variability in global age at onset and peak age across mental disorders. These findings can inform the timing and resource allocation regarding early intervention and preventive approaches.

To our best knowledge, this study is the first fully epidemiological and largest [35–38] meta-analysis on age at onset of mental disorders globally. It also represents the most comprehensive approach, encompassing all ICD-11 diagnostic spectra for which we found eligible studies, allowing comparative transdiagnostic analyses across different categories of mental disorders [39, 40]. Furthermore, per protocol, high-quality population-level studies were included that are less likely to be affected by biases, meeting previous methodological recommendations [30]. Moreover, data from all continents of the world were available, providing global estimates on the age at onset of mental disorders. Importantly, the statistical approach of this meta-analysis provides an estimate of age at disorder onset distribution throughout the lifespan, going beyond mere centrality estimates.

The meta-epidemiologic results of this work show that mental disorders have onset when dramatic biological changes in the brain occur, from childhood, through adolescence, to adulthood, that involves grey-matter density, cerebral metabolic rate, synaptic density, white matter growth and myelination [41]. The in-depth, robust epidemiological evidence provided here has several clinical implications. Firstly, the onset of the first mental disorder before age 14, 18 and 25 in one third, half and 62.5% and peak/median age of 15.5/18 years demonstrate that adult mental disorders originate early during the neurodevelopmental phases of life and peak, in pooled mental disorders, by mid to late adolescence [42–44]. Given that mental disorders are one of the five most common ailments leading to morbidity, mortality and dysfunction among young people worldwide [45], the current findings are relevant to policymakers and healthcare providers. These results suggest that the next generation of mental health research should prioritise designing and funding global early interventions [5, 46] and indicated, selective and/or universal preventive interventions for mental disorders during mid/late adolescence and young adulthood that are currently lacking [8].

Secondly, this study provides disorder-specific estimates of age at disorder onset that impacts mental health service configuration and delivery. For about half of mental disorders (Table 2), disorder onset occurs well before age 18. Disorder median age at onset occurred during the neurodevelopment, within age 14 for the vast majority of phobias and separation anxiety, ASD, ADHD, and for more then half in social anxiety disorders. For these disorders, good mental health promotion, along with preventive and early intervention approaches need to target these neurodevelopmental periods, mainly during pre-school and primary school periods. Many of the risk and protective factors that impact the neurodevelopment of individuals with these disorders are known [23, 47–52], and some of them may even impact the pre or perinatal phases [53]. A recent meta-umbrella review (i.e., a synthesis of systematic reviews of meta-analyses) has summarised risk and protective factors (beyond genetics) in a comprehensive atlas [54]. Importantly, targeting the neurodevelopmental phase has the potential to accommodate multi-endpoint numerators across mental disorders that are essential to better justify the denominator of efforts and costs for preventive and early intervention [8]. Higher  median age at disorder onset during the transitional period across adolescence and young adulthood emerged for another larger group of mental disorders, i.e., anorexia nervosa, bulimia nervosa, OCD, binge eating, and cannabis use disorders. For these disorders, good mental health promotion, prevention and early intervention could be delivered in primary and secondary schools [55–57]. A third group with median age at onset in the early adulthood included, schizophrenia, personality, panic and alcohol use disorders, and a fourth group included the remaining disorders that have a median age at onset in the later adulthood, such as PTSD, GAD, depressive and bipolar disorders, and ATPD. Secondary schools and colleges could become the most important setting for mental health promotion, prevention and early intervention across these two groups of mental disorders. Importantly, the diagnosis of personality disorder is artifactually delayed by diagnostic criteria allowing diagnosis after age 18, and clinically relevant symptoms occur earlier.

For all disorders culturally and ethically- sensitive [8] promotion of good mental health (indicated, selective and/or universal) prevention and early intervention should ideally be delivered in an integrated fashion that encompasses schools/colleges, and paediatricians/general practitioners, emergency departments, mental health settings, as well as the general community [60]. Overall, this study shows that any lower age threshold limiting access to mental health promotion campaigns or preventive or early interventions mental health programmes is not supported by meta-epidemiological evidence. Conversely, lower age thresholds that divide the education and training of mental health specialists or clinical services deprive individuals with developmental disorders (or other disorders with early onset) of continuity of care. Divisions fragment pathways to care and continued treatment from childhood through adolescence into adulthood (see below) [58]. Our recommendation for mental health services of the future would allow soft entry points, set no lower age threshold, and loosen lower age intake criteria [59]. For example, an indicated prevention and early intervention model of care for mood disorders, and schizophrenia-spectrum/primary psychotic disorders should ideally integrate low-threshold entry points for individuals of age younger than 18, supplemented by systematic promotion of good mental health, proactive screening for risk of developing specific mental disorders according to peak and median age at onset identified above, and deliver needs-based care [19, 60, 61].

Thirdly, a further, broader clinical implication of these results is that it demonstrates that age 18 as an intake threshold for adult mental health services is artificial and not based on global epidemiological evidence (nor on biological evidence of age when major brain changes occur [41]). To the best of our knowledge, mental health specialist training and mental health services in most parts of the world, including North America, Australia, most European countries, including Italy, Germany, UK, are divided between child/adolescent psychiatry and adult psychiatry. Given that the vast majority of mental disorders diagnoses seen in adulthood show a peak of onset before age 18, such a bifurcated mental health system is not evidence-based and may disadvantage individuals with developmental disorders from accessing and receiving adequate and continuous care [62]. Moreover, most psychiatry training programmes fail to target the transition period from childhood and adolescence to adult psychiatry [63]. Although many mental health services have tried to address this discontinuity of care [63, 64], significant gaps remain. Future mental health reforms could leverage and refine clinical high-risk services for young people at risk of psychosis, which typically accepts referrals aged 14–35 years and therefore provide essential transitional care to this vulnerable young population [15, 17, 58].

This study has several limitations. First, data were too sparse to calculate country-specific estimates. However, the database included many countries worldwide, and, therefore, the estimates provided are largely representative of the global general population. Second, the 95% confidence interval for some estimates were broad since analyses for some disorders were based on few studies. Third, data were heterogeneous, making traditional meta-analytic techniques unfeasible. We, therefore, applied advanced meta-analytic techniques, including bootstrap methods that helped to address this shortcoming and provide unified outcomes across all mental disorders. Fourth, our quality assessment was non-standardised because appropriate validated measures were not available. However, we per-protocol included the highest-quality studies to study age at onset according to previous recommendations [30]. Fifth, the definition of age at disorder onset was heterogeneous; we addressed this issue via sensitivity analyses. Sixth, we were not able to account for and differentiate among comorbid and standalone diagnoses. Sixth, we were not able to account for differences across regions. Finally, caution is needed when comparing peaks (e.g., between symptoms and diagnosis), in particular when the peak curves are different, and when comparing median age at onset across disorders (e-Table 5).

## Conclusions

The meta-analytic, global, epidemiological evidence provided challenges the current mental health care system that artificially separates child/adolescent and adult mental health services, providing strong epidemiologic evidence for the global implementation of integrated models of mental health promotion and preventive/early intervention care for young people in the community, those at risk for and with manifest mental disorders.

## Supplementary information

Supplementary
