# Supplementary material for: Age at onset of mental disorders worldwide: large-scale meta-analysis of 192 epidemiological studies
Source: Mol Psychiatry. 2021 Jun 2;27(1):281–95. doi: 10.1038/s41380-021-01161-7 (PMC8960395; doi:10.1038/s41380-021-01161-7)
Supplement: Supplementary file 1 — Supplementary [file 41380_2021_1161_MOESM1_ESM.pdf]

Supplementary material

Age at onset of mental disorders worldwide: large-scale meta-analysis of 192 epidemiological studies

Marco Solmi[1,2,3]*, Joaquim Radua[3,4,5]*, Miriam Olivola[3], Enrico Croce[6], Livia Soardo[7], Gonzalo Salazar de Pablo[3,8,9], Jae Il Shin[10], James B. Kirkbride[11], Peter Jones[12,13], Jae Han Kim,[14] Jong Yeob Kim,[14] Andrè F. Carvalho,[15] Mary Seeman,[16] Christoph U. Correll[17,18,19,20], Paolo Fusar-Poli [3,7,21,22].

Affiliations:
1 Neurosciences Department, University of Padua, Padua, Italy
2 Neuroscience Centre, University of Padua, Padua, Italy
3 Early Psychosis: Interventions and Clinical-detection (EPIC) lab, Department of Psychosis Studies, Institute of Psychiatry, Psychology& Neuroscience, King's College London, London, UK
4 Imaging Mood- and Anxiety-Related Disorders (IMARD) group, Institut d'Investigacions Biomèdiques August Pi i Sunyer (IDIBAPS); Mental Health Research Networking Center (CIBERSAM); Barcelona, Spain
5 Centre for Psychiatric Research and Education, Department of Clinical Neuroscience, Karolinska Institutet;
6 Institute of Psychiatry, Department of Biomedical and Specialty Surgical Sciences, University of Ferrara, Ferrara, Italy
7 Department of Brain and Behavioral Sciences, University of Pavia, Pavia, Italy;
8 Department of Child and Adolescent Psychiatry, Institute of Psychiatry, Psychology & Neuroscience, King's College London, London, UK
9 Institute of Psychiatry and Mental Health. Department of Child and Adolescent Psychiatry, Hospital General Universitario Gregorio Marañón School of Medicine, Universidad Complutense, Instituto de Investigación Sanitaria Gregorio Marañón (IiSGM), CIBERSAM, Madrid, Spain
10 Department of Pediatrics, Yonsei University College of Medicine, Seoul, South Korea
11 Division of Psychiatry, University College London, London, UK
12 Department of Psychiatry, University of Cambridge, Cambridge, England
13 CAMEO Early Intervention Service, Cambridgeshire and Peterborough National Health Service Foundation Trust, Cambridge, England
14 Yonsei University College of Medicine, Seoul 03722, Korea
15 IMPACT (Innovation in Mental and Physical Health and Clinical Treatment) Strategic Research Centre, School of Medicine, Barwon Health, Deakin University, Geelong, VIC, Australia
16 Department of Psychiatry, University of Toronto, Toronto, Canada
17 Department of Psychiatry, Zucker Hillside Hospital, Glen Oaks, NY, USA;
18 Department of Psychiatry and Molecular Medicine, Donald and Barbara Zucker School of Medicine at Hofstra/Northwell, Hempstead, NY, USA;
19 Center for Psychiatric Neuroscience, Feinstein Institute for Medical Research, Manhasset, NY, USA;
20 Department of Child and Adolescent Psychiatry, Charité-Universitätsmedizin Berlin, Berlin, Germany
21 OASIS service, South London and Maudsley NHS Foundation Trust, London, UK;
22 National Institute for Health Research, Maudsley Biomedical Research Centre, London, UK.

Corresponding author

Paolo Fusar-Poli

Early Psychosis: Interventions and Clinical-detection (EPIC) lab, Department of Psychosis Studies, Institute of Psychiatry, Psychology& Neuroscience, King's College London, London, UK

paolo.fusar-poli@kcl.ac.uk

Supplementary material - Table of contents

e-methods. Method for estimating of the histograms of age at disorder onset for each individual study included in the current meta-analysis

e-figure 1. PRISMA flow chart

e- figures 2-17. Curves of age at onset of specific mental disorders.

e-table 1. PRISMA check-list

e-table 2. MOOSE check-list

e-table 3. Characteristics of included studies

e-table 4. Studies excluded after full-text assessment, with reason for exclusion

e-table 5. Comparison of age at onset across mental disorders

e-methods

Methods for estimating the histograms of age at disorder onset for each individual study included in the current meta-analysis were the following. For studies reporting the proportion of individuals whose age at disorder onset was in each age group, we calculated the sum of square errors (SSE) between the proportions reported (observed) in the studies, and the proportions derived (expected) from the histogram as:

$$SSE_{i,j} = w_i \cdot \left(\frac{p_{i,j,observed} - p_{i,j,expected}}{width_{i,j}}\right)^2$$

where w_i is the weight of the ith study, $p_{i,j,observed}$ and $p_{i,j,expected}$ are the observed and expected proportions of individuals in the jth age group of the ith study, and $width_{i,j}$ is the width (in years) of the jth group of the ith study. The weight of a study was the product of the number of included individuals and the squared age range of these individuals; in pilot analyses, we had found that this weight returned estimations similar to the estimations found when only including large (n>1,000) studies. The expected proportions were previously scaled, so that their sum was one.

We applied the same calculations to studies reporting percentiles, as the percentiles define groups of age at disorder onset, e.g., if percentile 75% is 16-years of age at disorder onset and percentile 90% is 25-years of age at disorder onset, the group of individuals whose age at disorder onset was 16-25-year represent 15% of the individuals.

For studies reporting incidences in different age groups at disorder onset, we first converted the incidences into proportions assuming a simplified population age pyramid with a constant number of individuals with a given age until 50-year-old and then a linear decrease until 100-year-old. Afterward, we applied the same calculations as for studies reporting proportions.

Finally, for studies reporting descriptive statistics, we calculated the *SSE* between the statistics reported (observed) in the studies and the statistics derived (expected) from the histogram as:

$$SSE_{i,j} = k \cdot w_i \cdot \left(y_{i,j,observed} - y_{i,j,expected}\right)^2$$

where k is a constant to scale the errors, and $y_{i,j,observed}$ and $y_{i,j,expected}$ are the observed and expected j^{th} statistics of the ith study. In preliminary analyses, we found that with $k = 6.5e^{-7}$, the $SSE_{i,j}$ of a median introduced as a statistic was similar to the $SSE_{i,j}$ of a median introduced as the percentile 50%. We censored the expected statistics to the maximum age of the corresponding study.

We used the method from Nelder and Mead[1] iteratively to find the histogram with the minimum overall SSE. In pilot analyses, we had observed that this estimation sometimes led to spurious histogram peaks without clear relationship to the data. To minimize these spurious peaks, we smoothed the histogram and repeated the estimation from the smoothed histogram 30 times.

We conducted all analyses in R[2].

e-figure 1. PRISMA flow chart of study selection

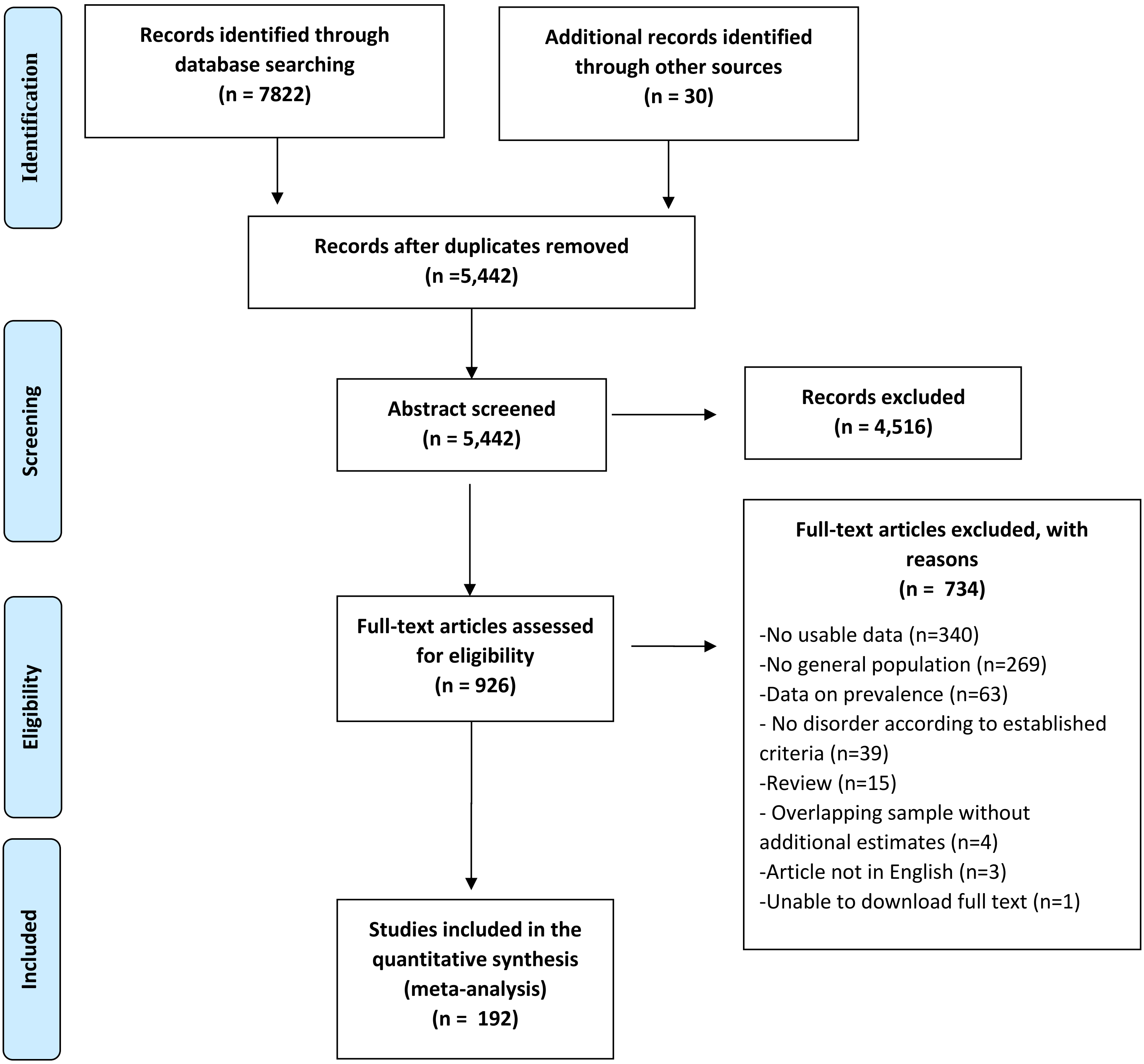

e-figures 2-17. Epidemiological proportion (y axis) and age peaks at onset (red line) of specific disorders in the general population, with 95%CIs (pink shadows).

e-figure 2. Curve of age at onset for attention deficit/hyperactivity disorder.

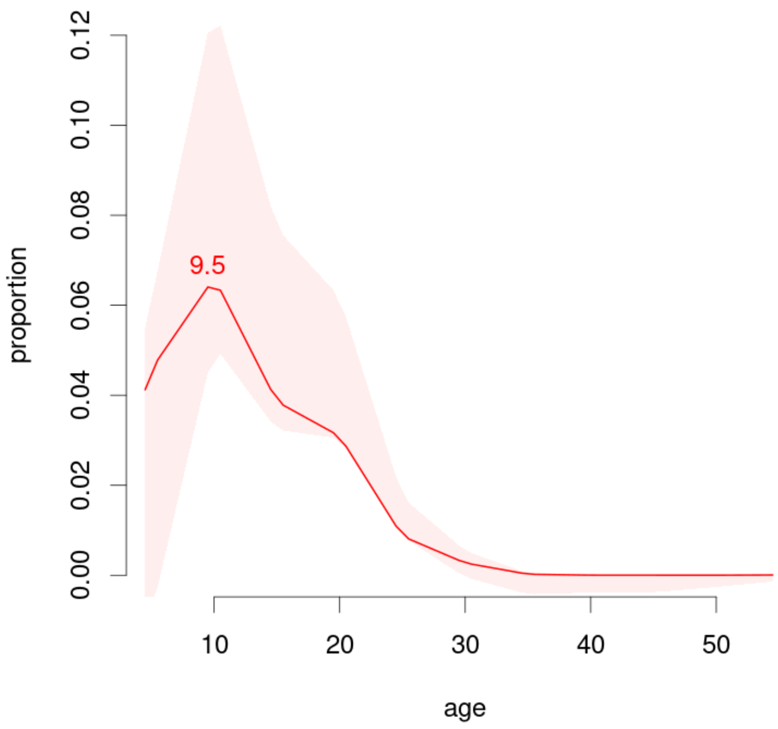

e-figure 3. Curve of age at onset for autism spectrum disorder.

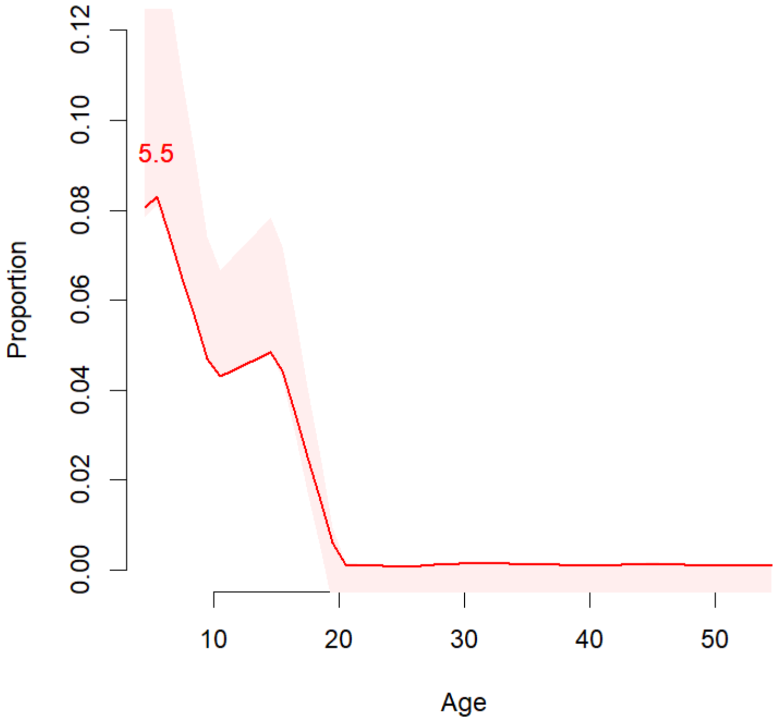

e-figure 4. Curve of age at onset for generalized anxiety disorder.

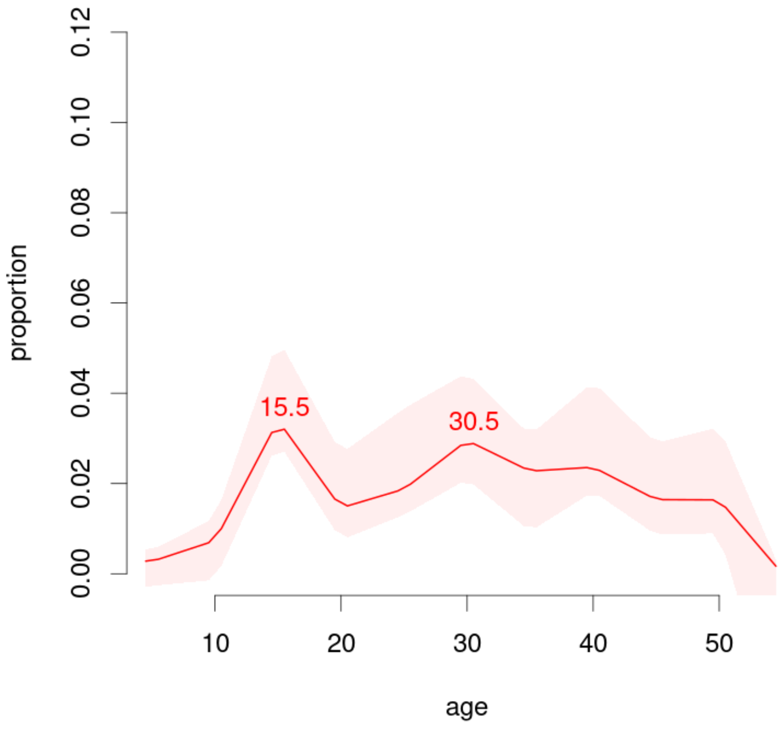

e-figure 5. Curve of age at onset for panic disorder.

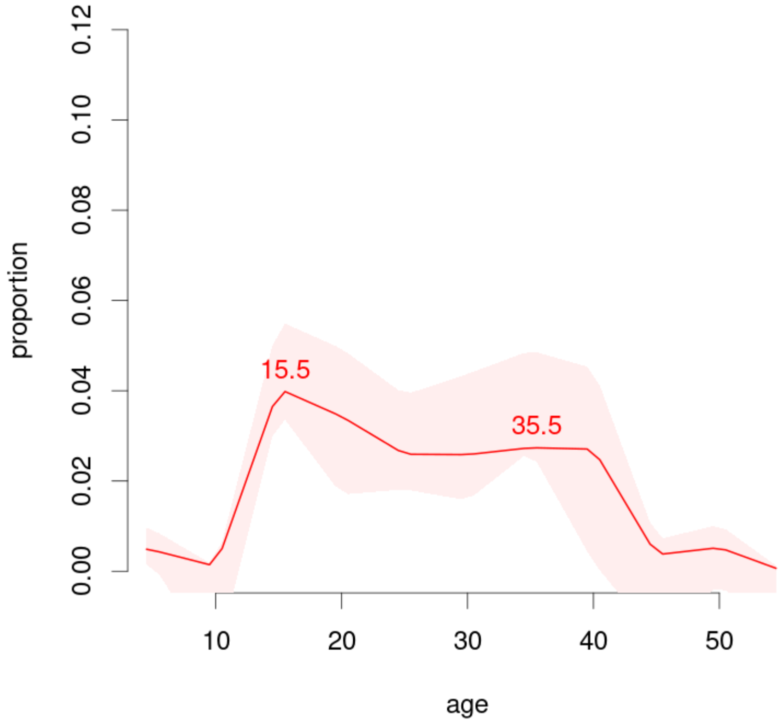

e-figure 6. Curve of age at onset for specific phobias and separation anxiety disorder.

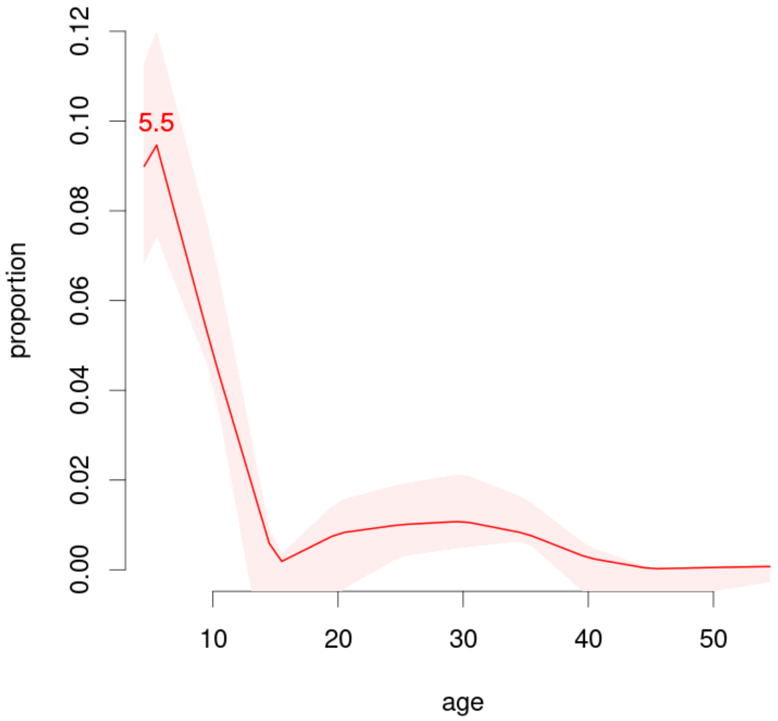

e-figure 7. Curve of age at onset for social anxiety disorder.

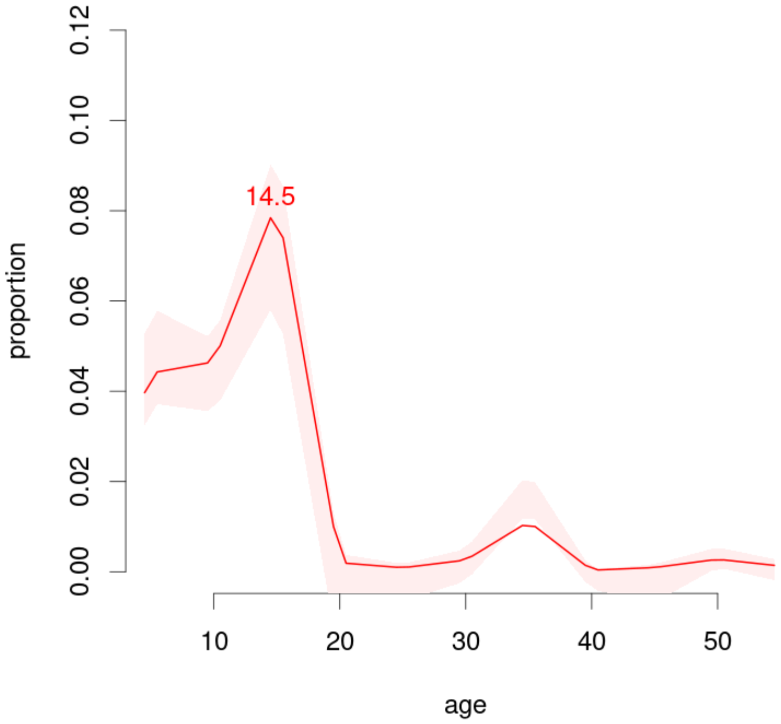

e-figure 8. Curve of age at onset for obsessive-compulsive disorder.

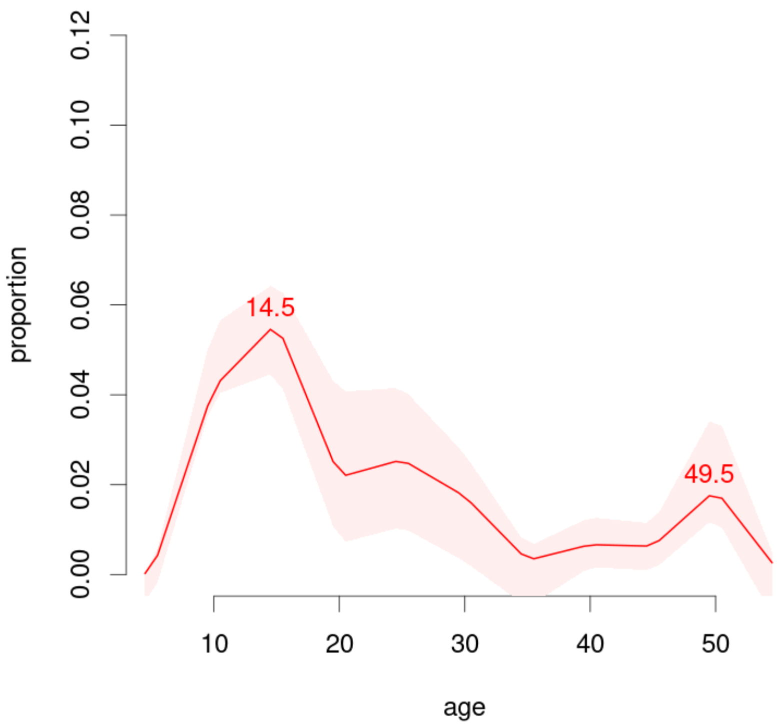

e-figure 9. Curve of age at onset for anorexia nervosa.

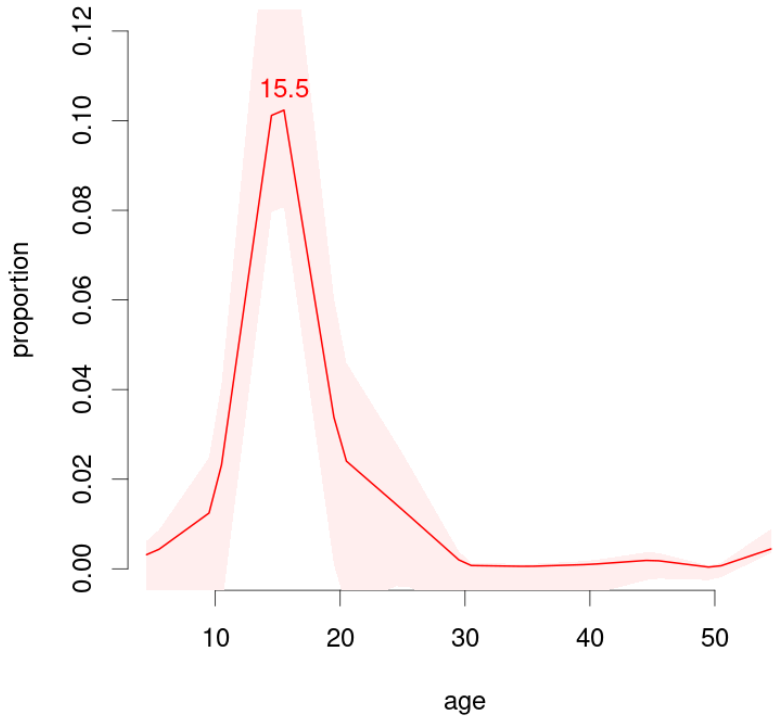

e-figure 10. Curve of age at onset for bulimia nervosa.

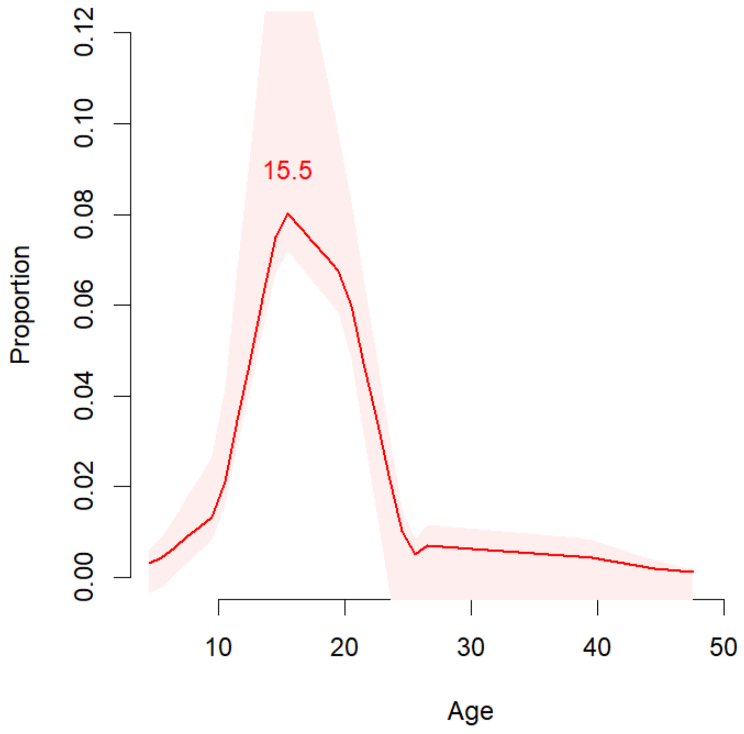

e-figure 11. Curve of age at onset for binge eating disorder.

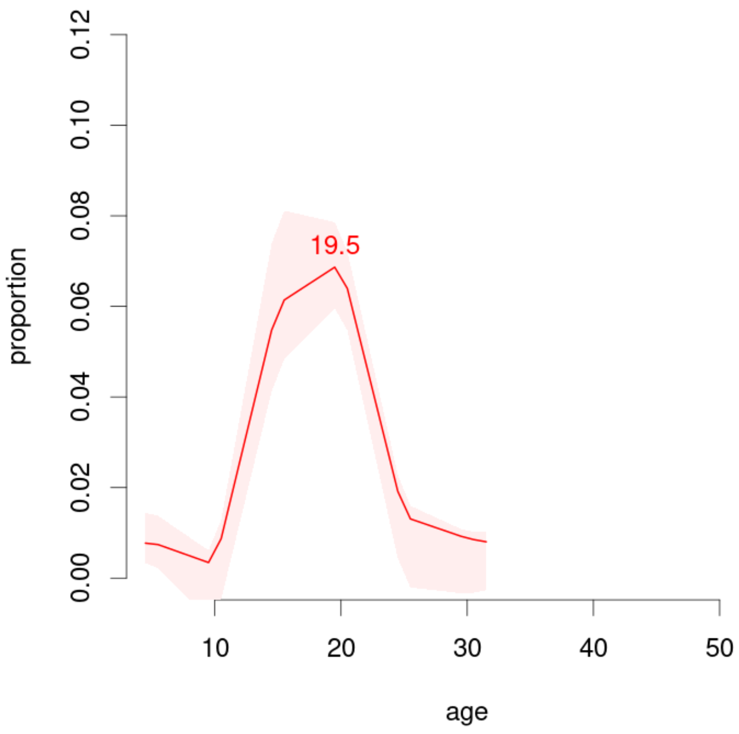

e-figure 12. Curve of age at onset for disorders due to use of alcohol.

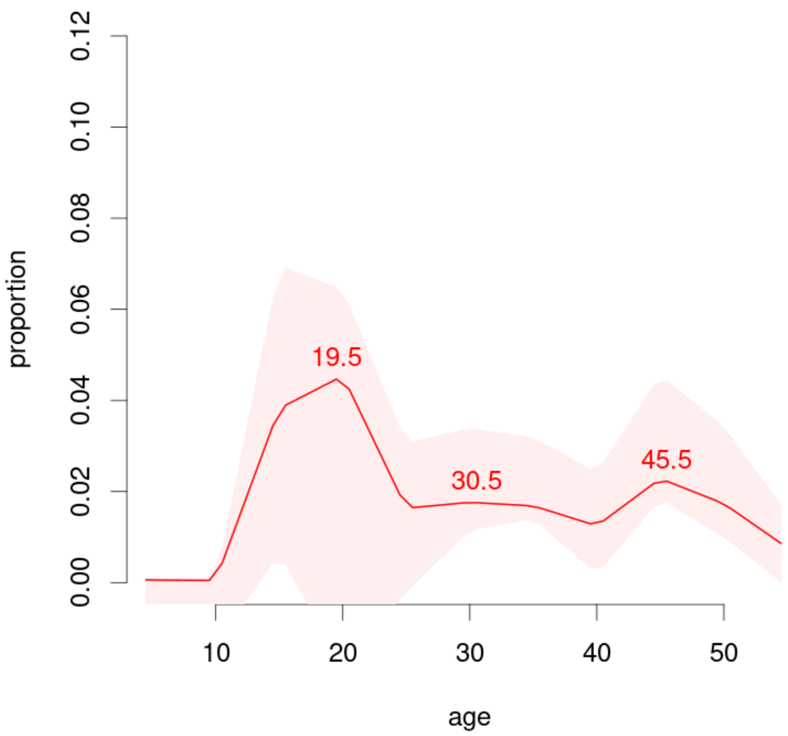

e-figure 13. Curve of age at onset for disorders due to use of cannabis.

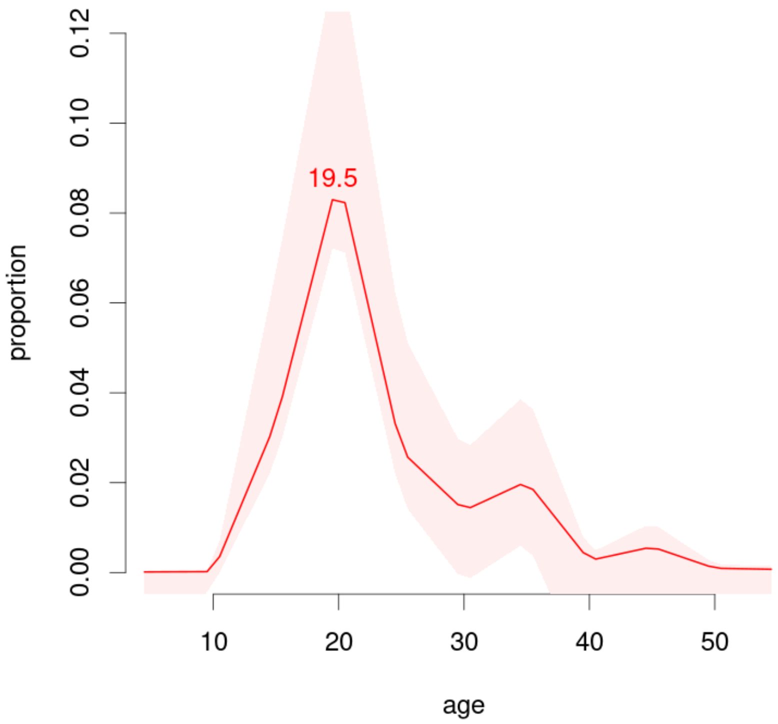

e-figure 14. Curve of age at onset for depressive disorder.

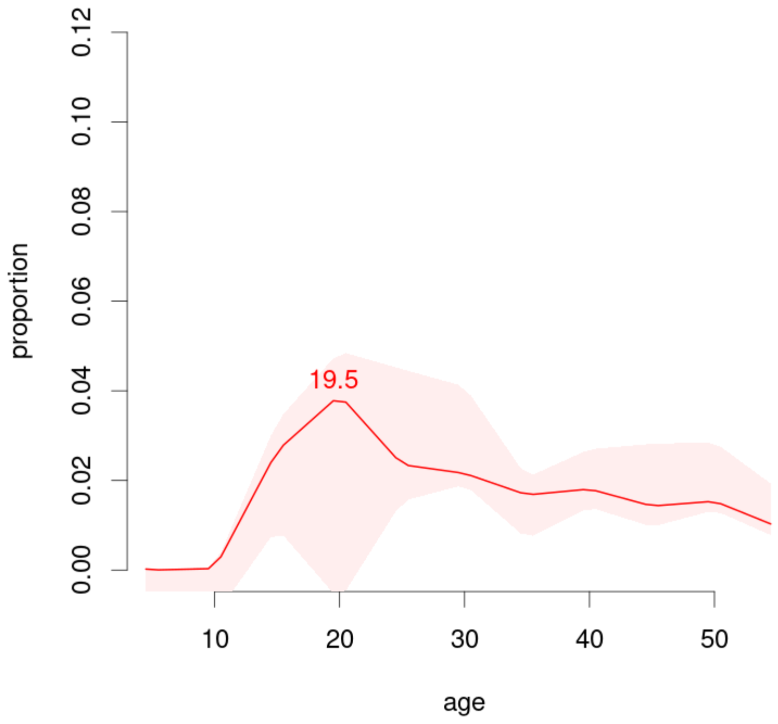

e-figure 15. Curve of age at onset for bipolar or related disorder.

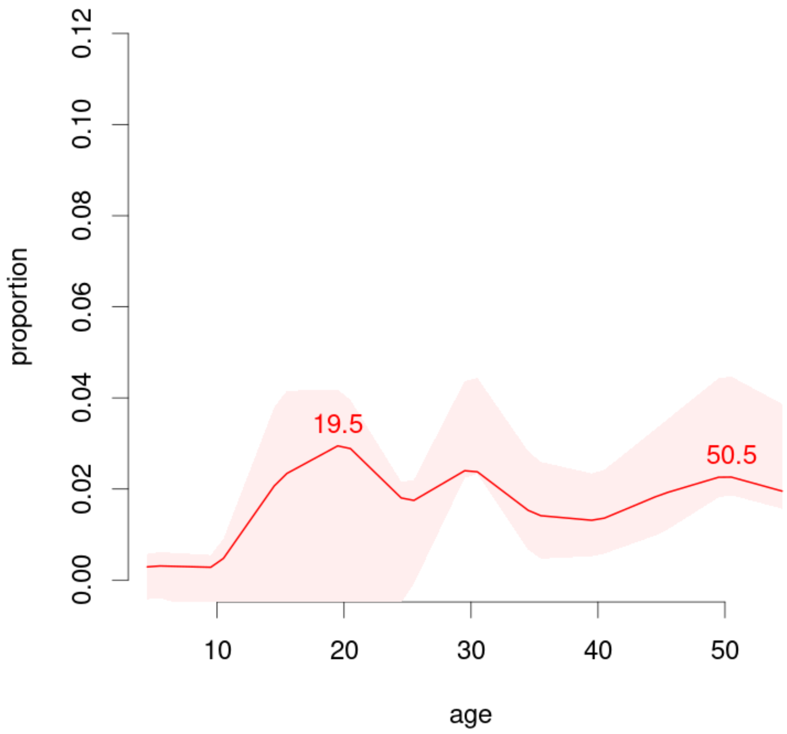

e-figure 16. Curve of age at onset for post-traumatic stress disorder.

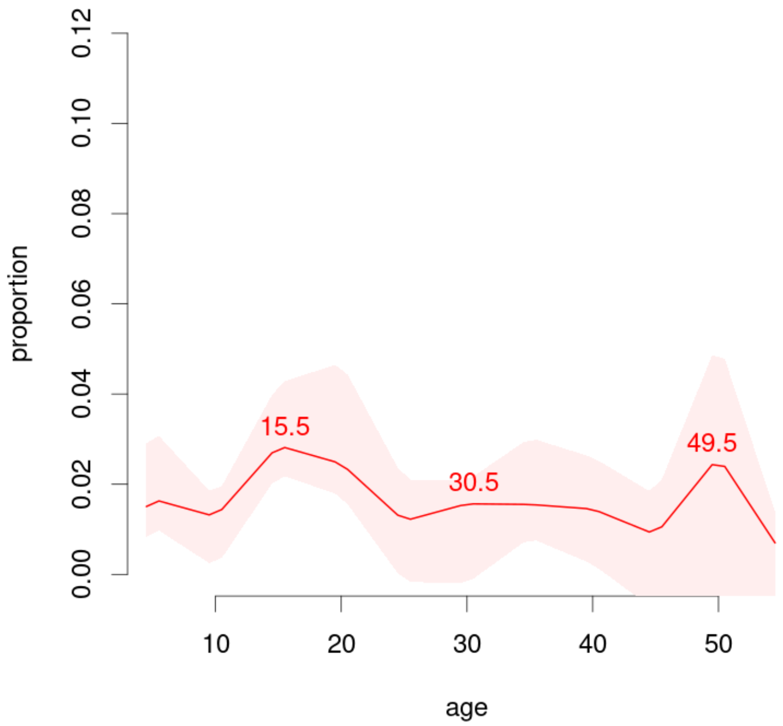

e-figure 17. Curve of age at onset for schizophrenia.

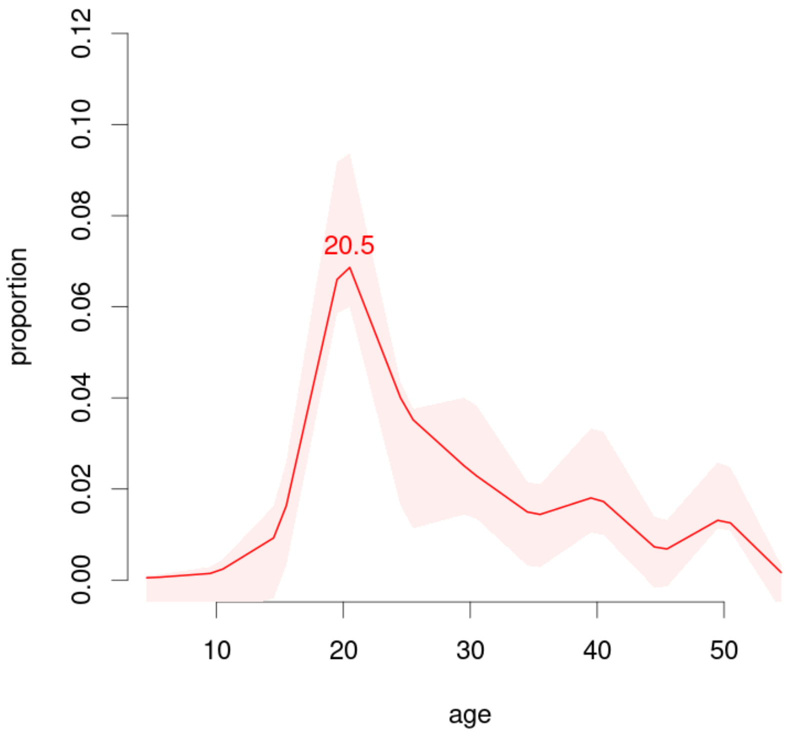

e-table 1. PRISMA check-list[3]

Section/topic	#	Checklist item	Reported on page #
**TITLE**			
Title	1	Identify the report as a systematic review, meta-analysis, or both.	1
**ABSTRACT**			
Structured summary	2	Provide a structured summary including, as applicable: background; objectives; data sources; study eligibility criteria, participants, and interventions; study appraisal and synthesis methods; results; limitations; conclusions and implications of key findings; systematic review registration number.	3
**INTRODUCTION**			
Rationale	3	Describe the rationale for the review in the context of what is already known.	4
Objectives	4	Provide an explicit statement of questions being addressed with reference to participants, interventions, comparisons, outcomes, and study design (PICOS).	4
**METHODS**			
Protocol and registration	5	Indicate if a review protocol exists, if and where it can be accessed (e.g., Web address), and, if available, provide registration information including registration number.	5
Eligibility criteria	6	Specify study characteristics (e.g., PICOS, length of follow-up) and report characteristics (e.g., years considered, language, publication status) used as criteria for eligibility, giving rationale.	5
Information sources	7	Describe all information sources (e.g., databases with dates of coverage, contact with study authors to identify additional studies) in the search and date last searched.	5
Search	8	Present full electronic search strategy for at least one database, including any limits used, such that it could be repeated.	5
Study selection	9	State the process for selecting studies (i.e., screening, eligibility, included in systematic review, and, if applicable, included in the meta-analysis).	5
Data collection process	10	Describe method of data extraction from reports (e.g., piloted forms, independently, in duplicate) and any processes for obtaining and confirming data from investigators.	5
Data items	11	List and define all variables for which data were sought (e.g., PICOS, funding sources) and any assumptions and simplifications made.	5

Risk of bias in individual studies	12	Describe methods used for assessing risk of bias of individual studies (including specification of whether this was done at the study or outcome level), and how this information is to be used in any data synthesis.	6
Summary measures	13	State the principal summary measures (e.g., risk ratio, difference in means).	6
Synthesis of results	14	Describe the methods of handling data and combining results of studies, if done, including measures of consistency (e.g., I^2) for each meta-analysis.	6
Risk of bias across studies	15	Specify any assessment of risk of bias that may affect the cumulative evidence (e.g., publication bias, selective reporting within studies).	6
Additional analyses	16	Describe methods of additional analyses (e.g., sensitivity or subgroup analyses, meta-regression), if done, indicating which were pre-specified.	6
**RESULTS**			
Study selection	17	Give numbers of studies screened, assessed for eligibility, and included in the review, with reasons for exclusions at each stage, ideally with a flow diagram.	7, figure 1
Study characteristics	18	For each study, present characteristics for which data were extracted (e.g., study size, PICOS, follow-up period) and provide the citations.	e-table 3
Risk of bias within studies	19	Present data on risk of bias of each study and, if available, any outcome level assessment (see item 12).	e-table 3
Results of individual studies	20	For all outcomes considered (benefits or harms), present, for each study: (a) simple summary data for each intervention group (b) effect estimates and confidence intervals, ideally with a forest plot.	-
Synthesis of results	21	Present results of each meta-analysis done, including confidence intervals and measures of consistency.	figures 2-4, table 1-2, e-figures 2-17
Risk of bias across studies	22	Present results of any assessment of risk of bias across studies (see Item 15).	e-table 3
Additional analysis	23	Give results of additional analyses, if done (e.g., sensitivity or subgroup analyses, meta-regression [see Item 16]).	8, table 2, e-table 5
**DISCUSSION**			
Summary of evidence	24	Summarize the main findings including the strength of evidence for each main outcome; consider their relevance to key groups (e.g., healthcare providers, users, and policy makers).	8
Limitations	25	Discuss limitations at study and outcome level (e.g., risk of bias), and at review-level (e.g., incomplete retrieval of identified research, reporting bias).	10
Conclusions	26	Provide a general interpretation of the results in the context of other evidence, and implications for future research.	10

**FUNDING**			
Funding	27	Describe sources of funding for the systematic review and other support (e.g., supply of data); role of funders for the systematic review.	10

e-table 2. MOOSE Checklist for Meta-analyses of Observational Studies [4]

Item No	Recommendation	Reported on Page No
Reporting of background should include		
1	Problem definition	4
2	Hypothesis statement	4
3	Description of study outcome(s)	7
4	Type of exposure or intervention used	5
5	Type of study designs used	5
6	Study population	5
Reporting of search strategy should include		
7	Qualifications of searchers (eg, librarians and investigators)	5
8	Search strategy, including time period included in the synthesis and key words	5
9	Effort to include all available studies, including contact with authors	-
10	Databases and registries searched	5
11	Search software used, name and version, including special features used (eg, explosion)	5
12	Use of hand searching (eg, reference lists of obtained articles)	5
13	List of citations located and those excluded, including justification	e-table 3, e-table 4
14	Method of addressing articles published in languages other than English	-
15	Method of handling abstracts and unpublished studies	-
16	Description of any contact with authors	5
Reporting of methods should include		
17	Description of relevance or appropriateness of studies assembled for assessing the hypothesis to be tested	4-5
18	Rationale for the selection and coding of data (eg, sound clinical principles or convenience)	6
19	Documentation of how data were classified and coded (eg, multiple raters, blinding and interrater reliability)	5

20	Assessment of confounding (eg, comparability of cases and controls in studies where appropriate)	6
21	Assessment of study quality, including blinding of quality assessors, stratification or regression on possible predictors of study results	6
22	Assessment of heterogeneity	6
23	Description of statistical methods (eg, complete description of fixed or random effects models, justification of whether the chosen models account for predictors of study results, dose-response models, or cumulative meta-analysis) in sufficient detail to be replicated	6
24	Provision of appropriate tables and graphics	Tables 1-2, e-tables 5, Figures 2-4, e-figures 2-17
Reporting of results should include		
25	Graphic summarizing individual study estimates and overall estimate	Figures 2-4
26	Table giving descriptive information for each study included	e-table 3
27	Results of sensitivity testing (eg, subgroup analysis)	Table 2, e-table 5
28	Indication of statistical uncertainty of findings	Figures 2-4, e-figures 2-17
Reporting of discussion should include		
29	Quantitative assessment of bias (eg, publication bias)	-
30	Justification for exclusion (eg, exclusion of non-English language citations)	-
31	Assessment of quality of included studies	6, e-table 3
Reporting of conclusions should include		
32	Consideration of alternative explanations for observed results	-
33	Generalization of the conclusions (ie, appropriate for the data presented and within the domain of the literature review)	10
34	Guidelines for future research	10
35	Disclosure of funding source	10

e-table 3. Characteristics of included studies

Author	Country	Design	Study	Age	Female %	Population	Individuals	Disorders	Diagnostic criteria	Onset definition
Abajobir[5]	Australia	BC	Mater Hospital-University of Queensland Study of Pregnancy	21+	ns	2,526	1,296	Cannabis abuse disorder, cannabis dependence	DSM-IV, ICD-10	First symptom
Al- Hamzawi [6]	Iraq	CS	Iraqi Mental Health Survey	18+	50	4,332	74	Intermittent explosive disorder	DSM-IV	First symptom
Alem [7]	Ethiopia	PC	Rural community	15 to 49	ns	68,378	295	Schizophrenia	ICD-10	First symptom
Al-Hamzawi [8]	Iraq	CS	Iraqi Mental Health Survey	18+	50	4,332	187	Intermittent explosive disorder, major depressive disorder	DSM-IV	First symptom
Angst [9]	U.S.	CS	National Comorbidity Survey Replication	13+	Ns	5,692	1,093	Bipolar disorder, major depressive disorder	DSM-IV	First symptom
Angst [10]	Switzerland	PC	The Zurich Study	20 to 40	ns	4,547	591	Generalized anxiety disorder	ICD-10, DSM-III	First symptom
Angst[11]	Switzerland	PC	The Zurich Study	20 to 30		4,547	591	Major depressive disorder	DSM-III	First diagnosis
Bantjes [12]	South Africa	CS	University students	0-21	55	1,402	540	Alcohol use disorder, bipolar disorder, generalized anxiety disorder, major depressive disorder, substance use disorder.	ICD-10, DSM-IV	First symptom
Becker [13]	Germany	PC	Dresden Study of Mental Health	18 to 24	ns	2,064	107	Specific phobia for animals, heights, environments, storm, water, blood, situational, flights, lift, driving, physical, doctors, vomiting, infections, others.	DSM-IV	First symptom
Bienvenu[14]	U.S.	CS	Baltimore ECA Follow-up Study	18+	ns	1,724	60	Specific phobia	DSM-III	First symptom
Birrell [15]	Australia	CS	Australian National Survey of Mental Health and Wellbeing	16 to 85	ns	8,841	225	Bipolar disorder, major depressive disorder, dysthymia	DSM-IV	First diagnosis
Blanco [16]	U.S.	CS	National Epidemiologic Survey on Alcohol and Related Conditions	18+	Ns	43,093	7,124	Major depressive episode	DSM-IV	First diagnosis
Bland [17]	Canada	CS	Edmonton survey	18+	ns	3,258	198	Alcohol abuse, alcohol dependence, any substance use disorder, antisocial personality disorder, major depressive disorder, manic episode, panic disorder, obsessive-compulsive disorder, phobia,,schizophrenia.	DSM-III	First symptom
Bogren[18]	Sweden	CS	The Lundby Cohort	0 to 92	48.70	3,505	432	Bipolar depression, dysthymic disorder, depressive disorder NOS, major depressive disorder, melancholic mood disorder, non-melancholic mood disorder, other mood disorders	DSM-IV	First diagnosis
Bonnewyn [19]	Belgium	CS	European Study on the Epidemiology of Mental Disorders	18+	52	2,419	367	Alcohol use disorder, bipolar disorder, dysthymia, generalized anxiety disorder, major depressive disorder, panic disorder, post-traumatic stress disorder, social phobia, specific phobia.	DSM-IV	First symptom
Borgers[20]	Mexico	CS	Mexican National Comorbidity Survey (2001-2002)	18 to 65	ns	5,782	1,588	Alcohol abuse, alcohol dependence, bipolar disorder, dysthymia, generalized anxiety disorder, major depressive disorder, panic disorder, social phobia, specific phobia, substance abuse, substance dependence	DSM-IV	First symptom
Bromet [21]	Belgium, Brazil, China, Colombia, France, Germany, India, Israel, Italy, Japan, Lebanon, Mexico, Netherlands, New Zealand, South Africa, Spain, Ukraine, USA	CS	World Mental Health Survey	18+	66.6	89,037	11,525	Major depressive episode	DSM-IV	First diagnosis
Bromet [22]	Ukraine	CS	ns	18+	ns	4,725	ns	Agoraphobia, alcohol dependence, bipolar disorder, dysthymia, generalized anxiety disorder, major depressive disorder, obsessive-compulsive disorder, panic disorder, post-traumatic stress disorder, social phobia, specific phobia, intermittent explosive disorder	DSM-IV	First symptom
Buckner [23]	U.S.	CS	National Epidemiological Survey on	18+	ns	43,093	1,643	Alcohol dependence, social anxiety disorder	DSM-IV	First diagnosis

Author	Country	Design	Study	Age	Female %	Population	Individuals	Disorders	Diagnostic criteria	Onset definition
			Alcohol AND Related Conditions							
Burke [24]	U.S.	CS	National Institute of Mental Health Epidemiologic Catchment Area Program,	18+	ns	20,861	9,127	Alcohol dependence, bipolar disorder, depressive disorder, obsessive-compulsive disorder, panic disorder, specific phobia, substance dependence	DSM-III	First diagnosis
Burnhams [25]	South Africa	CS	World Mental Health Survey	18+	ns	4,315	380	Alcohol abuse, alcohol dependence	DSM-IV	First diagnosis
Burns[26]	South Africa	PC	Cohort from the Province of KwaZulu-Natal	16+	ns	ns	54	Schizophrenia spectrum disorders	DSM-IV-TR	First symptom
Burstein [27]	U.S.	CS	National Comorbidity Survey-Adolescent Supplement	13-18	51	10,123	1,742	Social phobia	DSM-IV	First symptom
Burstein [28]	U.S.	CS	National Comorbidity Survey-Adolescent Supplement	13-18	51	10,123	3,826	Specific phobia	DSM-IV	First symptom
Caraveo-Anduaga[29]	Mexico	CS	Mexico City Cohort	18 to 65	ns	1,932	27	Obsessive-compulsive disorder	ICD-10	ns
Castagnini [30]	Denmark	CS	ns	15 to 64	ns	3,565,833	3,350	Acute and transient psychotic disorder, bipolar disorder, schizophrenia	ICD-10	First diagnosis
Chang [31]	Hong Kong	PC	Early assessment service for young people with psychosis	15 to 25	ns	ns	461	Psychotic mood disorder, schizophrenia,	ICD-10	First symptom
Chapman [32]	Australia	CS	Australian National Survey of Mental Health and Wellbeing	16-85	Ns	8,841	ns	Post-traumatic stress disorder	DSM-IV	ns
Cheng[33]	Taiwan	CS	The Taiwan Aboriginal Study Project	15+	ns	993	478	Alcohol dependence	DSM-III-R, ICD-10	Ns
Cho [34]	South Korea	CS	Korean Epidemiologic Catchment Area Study Replication	18 to 64	60.4	6,510	1,601	Alcohol abuse, alcohol dependence, dysthymia, generalized anxiety disorder, major depressive disorder, obsessive-compulsive disorder, post-traumatic stress disorder, specific phobia	DMS IV	First symptom
Chong [35]	Singapore	CS	Singapore Mental Health Study	18+	ns	6,616	417	Major depressive disorder	ICD-10, DSM-IV	First symptom
Christie[36]	U.S.	CS	NIHM Epidemiologic Catchment Area	18+	ns	18,572	5,202	Alcohol abuse, alcohol dependence, anxiety disorders, bipolar disorder, major depressive disorder, substance abuse, substance dependence	DSM-III	First symptom
Cìa [37]	Argentina	CS	Argentinean Study of Mental Health Epidemiology	18 to 75	ns	3,927	1,032	Alcohol abuse, alcohol dependence, attention deficit/hyperactivity disorder, bipolar disorder, dysthymia, generalized anxiety disorder, major depressive disorder, obsessive-compulsive disorder, oppositional-defiant disorder, panic disorder, post-traumatic stress disorder, separation anxiety disorder, social phobia, specific phobia, substance abuse, substance dependence	DSM-IV	First diagnosis
Cillicilli[38]	Turkey	CS	Konya Cohort	18+	52.9	3,012	89	Obsessive-compulsive disorder	DSM-IV	ns
Cooper [39]	Croatia	PC	Croatian Psychiatry Case Register	0-31	ns	80,445	464	Schizophrenia	Hospital record	First hospitalization
Dakwar [40]	U.S.	CS	National Epidemiologic Survey on Alcohol and Related Conditions	0-18	ns	34,653	616	Attention deficit/hyperactivity disorder	DSM-IV	First diagnosis
Dalsgaard [41]	Denmark	BC	Danish registry	0-18	ns	1,300,000	99,926	Acute and transient psychosis, alcohol abuse, anorexia nervosa, anxiety disorder, attachment disorder, attention deficit/hyperactivity disorder, Asperger syndrome, autism-spectrum disorders, bipolar disorder, bulimia nervosa, cannabis abuse, depression, eating disorder, intellectual disability, mood disorder, obsessive-compulsive disorder, other developmental disorders, personality disorder, schizophrenia-spectrum disorders, substance use disorder, tic disorder.	ICD-10	First diagnosis
De Jonge [42]	Australia, Belgium, Brazil, Bulgaria, China, Colombia, France,	CS	World Mental Health Survey	18+	ns	142,949	2,430	Panic disorder	DSM-V	First diagnosis

Author	Country	Design	Study	Age	Female %	Population	Individuals	Disorders	Diagnostic criteria	Onset definition
	Germany, Iraq, Israel, Italy, Japan, Lebanon, Mexico, New Zealand, Nigeria, Northern Ireland, Peru, Poland, Portugal, Romania, Spain, The Netherlands, Ukraine, U.S.									
De Vries[43]	The Netherlands	CS	Netherlands Mental Health Survey and Incidence Study-2 (NEMESIS-2)	18 to 64	49.58	5,302	2,236	Any mental disorder	DSM-IV	ns
Degenhardt [44]	Australia	CS	Australian National Survey of Mental Health and Wellbeing	18 to 65	50.4	8,463	1,582	Alcohol abuse, alcohol dependence, cannabis abuse, cannabis dependence	DSM-IV	ns
Deutsch [45]	Australia	CS	Australian Twin Registry Cohort II and Cohort III	24 to 40	38.04	5,946	ns	Alcohol use disorder	Assessment for the Genetics of Alcoholism	First symptom
Dussault [46]	Canada	BC	Adolescents' Self-Report of Gambling	14-18	36	ns	297	Gambling	DSM-IV	First symptom
Esan [47]	Nigeria	CS	The Nigerian national survey of mental health and well-being	18+	51.0	6,752	ns	Alcohol abuse, alcohol dependence	DSM-IV	First symptom
Ezpeleta[48]	Spain	CS	Barcelona Preschool Cohort	3 to 9	50	622	156	Oppositional defiant disorder	DSM-IV	Fisrt symptom
Falk [49]	U.S.	CS	National Epidemiological Survey on Alcohol AND Related Conditions	18+	ns	43,093	6,427	Alcohol abuse, alcohol dependence, bipolar disorder, dysthymia, generalized anxiety disorder, major depressive disorder, panic disorder, social phobia, specific phobia.	DSM-IV	First diagnosis
Faravelli [50]	Italy	CS	National Health system from Sesto Fiorentino	0+	ns	23,435	555	Social phobia	DSM-IV	First symptom
Farmer [51]	U.S.	CS	Oregon Adolescent Depression Project	16 to 30	59	816	156	Cannabis use disorder	DSM-IV	First diagnosis
Fava [52]	U.S.	CS	National Comorbidity Survey Replication	18+	Ns	9,282	977	Major depressive episode	DSM-IV	First symptom
Fernandez-Pujals[53]	UK	CS	Scottish Family Health Study	18+	59	20,198	2,726	Major depressive disorder	DSM-IV	na
Fogarty [54]	Canada	CS	ns	18+	ns	3,258	22	Bipolar disorder	DSM-III	First symptom
Gabilondo [55]	Spain	CS	Community survey: ESEMeD-Spain	18+	ns	5,473	167	Major depressive disorder	ICD-10, DSM-IV	First symptom
Garfinkel [56]	Canada	CS	Mental Health Supplement to the Ontario Health Survey	15 to 64	ns	8,116	62	Bulimia nervosa	DSM-III-R	First diagnosis
Gilder [57]	U.S.	CS	Community survey on American Indian	18 to 70	ns	580	254	Alcohol abuse, alcohol dependence	DSM-III-R	First diagnosis
Gilder [58]	U.S.	CS	Community survey on American Indian	18+	ns	777	284	Alcohol use disorder, cannabis use disorder, stimulant use disorder, substance use disorder	DSM-V	First diagnosis
Gilman [59]	U.S.	PC	Providence, Rhode Island cohort of the National Collaborative Perinatal Project	0+	47.4	1,089	272	Major depressive disorder	DSM-III/IV	First diagnosis
Glantz [60]	Belgium, Brazil, Bulgaria, China, Colombia, France, Germany, India, Israel, Italy, Japan, Lebanon, Mexico, Netherlands, New Zealand, Nigeria, Romania, South Africa, Spain, Ukraine, U.S.	CS	Alcohol Abuse in the WMH Surveys	18+	ns	51,773	1,967	Alcohol abuse	DSM-IV	First symptom
Goncalves [61]	Australia	CS	National Survey of Mental Health and Well-Being	55-85	Ns	3,178	311	Generalized anxiety disorder	DSM-IV	First symptom
Gonzalez [62]	U.S.	CS	Collaborative Psychiatric Epidemiology Surveys	18+	Ns	17,967	4,797	Major depressive disorder	DSM-IV	First symptom
Griesler [63]	U.S.	PC	Ns	11-16	52.9	814	700	Anxiety disorders, disruptive behavior disorder, mood disorders, nicotine dependence	DSM-IV	First symptom
Gureje [64]	Nigeria	CS	Nigerian Survey of Mental Health and Wellbeing	18+	50.9	6,752	74	Major depressive episode	DSM-IV	First symptom
Gureje, [65]	Nigeria	CS	Nigerian Survey of Mental Health and	18+	41.5	6,752	440	Anxiety disorders, any disorder, mood disorders,	DSM-IV	First diagnosis

Author	Country	Design	Study	Age	Female %	Population	Individuals	Disorders	Diagnostic criteria	Onset definition
			Wellbeing					substance use disorders		
Hafner [66]	Denmark, Germany	BC	Danish and the Mannheim registers	12 to 59	Ns	5,065,000	527	Schizophrenia spectrum, schizophrenia	ICD-8	First hospitalization
Hafner [67]	Germany	CS	ABC study	12 to 59	52.4	1,500,000	267	Schizophrenia	ICD-9	First symptom or hospitalization
Hahm [68]	South Korea	CS	Korean Community	18 to 64	40,7	1,059	59	Alcohol use disorder	DSM-IV	First symptom
Hardeveld [69]	Netherlands	PC	The Netherlands Mental Health Survey and Incidence Study	18-64	68.0	ns	687	Major depressive disorder	DSM-IIIR	ns
Hines [70]	Australia	CS	Australian twins and siblings Registry	12+	ns	3,798	371	Cannabis dependence	DSM-IV	First symptom
Hoertel[71]	U.S.	CS	National Epidemiologic Survey on Alcohol and Related Conditions (NESARC)	18+	ns	34,653	2,587	Major depressive disorder	DSM-IV	First diagnosis
Hofmeijer-Sevink [72]	Netherlands	PC	Netherlands Study of Depression and Anxiety	18 to 73	ns	2,981	1,004	Agoraphobia, generalized anxiety disorder, obsessive-compulsive disorder, panic disorder, post-traumatic stress disorder, social phobia, specific phobia	DSM-IV	First symptom
Hsu [73]	Taiwan	CS	Taiwan's National Health Insurance Database	0-18	ns	ns	401	Schizophrenia	ICD-9	First diagnosis
Hudson [74]	U.S.	CS	National Representative Survey of the U.S. NCS-R	18+	ns	9,282	23	Anorexia nervosa, binge eating disorder, bulimia nervosa	DSM-IV	First symptom
Isohanni[75]	Finland	CS	Northern Finland 1966 Birth Cohort	16 to 29	ns	10,581	409	Schizophrenia spectrum, other than psychotic disorders	DSM-III	Firs hospitalization
Joinson [76]	UK	BC	Avon Longitudinal Study of Parents and Children	10 to 20	ns	9,193	2,633	Major depressive disorder	ICD-10	First diagnosis
Jones [77]	UK	BC	Medical Research Council National Survey of Health and Development	16 to 43	48	4,746	30	Schizophrenia	DSM-III-R	First diagnosis
Karam [78]	Belgium, Brazil, Bulgaria, China, Colombia, France, Germany, Italy, Israel, Japan, Lebanon, Mexico, Netherlands, New Zealand, Northern Ireland, Romania, South Africa, Spain, Ukraine, U.S.	CS	World Mental Health Survey	18+		51,295	1,042	Post-traumatic stress disorder	DSM-IV	First diagnosis
Karam [79]	Lebanon	CS	Lebanese Evaluation of the Burden of Ailments and Needs Of the Nation	18+	54.6	2,857	491	Alcohol abuse, bipolar disorder, dysthymia, generalized anxiety disorder, intermittent explosive disorder, major depressive disorder, post-traumatic stress disorder, separation anxiety disorder, social phobia, specific phobia	DSM-IV	ns
Kasch [80]	U.S.	CS	The Epidemiologic Catchment Area study	18+	ns	18,571	180	Alcohol abuse, alcohol dependence, any substance use disorder, antisocial personality disorder, bipolar disorder, dysthymia, major depressive disorder, phobia, obsessive-compulsive disorder, panic disorder, schizophrenia, somatoform disorder	DSM-III	First symptom
Kathleen [81]	Brazil, Bulgaria, China, Colmbia, India, Japan, Lebanon, Mexico, New Zealand, Romania, U.S.	CS	World Mental Health Survey	18+	ns	61,392	721	Bipolar disorder	DSM-IV	First symptom
Kebede [82]	Ethiopia	CS	Community survey in Butajira	15 to 49	ns	2,285	322	Schizophrenia	ICD-10	First symptom
Keenan [83]	U.S.	PC	Pittsburgh Girls Study	7-15	100	2,393	560	Conduct disorder	DSM-IV	First symptom
Kendell [84]	Ireland, Scotland	PC	Edinburgh psychiatric case register, Common services agency of the Scottish health service	15 to 39	ns	5,670,000	6,119	Schizophrenia, mood disorders	ICD-9	First hospitalization
Kessler [85]	U.S.	CS	National Comorbidity Survey-Adolescent Supplement	13-18	Ns	6,483	ns	Agoraphobia with/without panic, alcohol abuse, alcohol dependence, attention deficit hyperactivity	DSM-IV	First symptom

Author	Country	Design	Study	Age	Female %	Population	Individuals	Disorders	Diagnostic criteria	Onset definition
								disorder, bipolar disorder, conduct disorder, eating disorder, generalized anxiety disorder, major depressive episode, oppositional defiant disorder, panic disorder, post-traumatic stress disorder, separation anxiety disorder, social phobia, specific phobia, substance abuse, substance dependence		
Kessler [86]	U.S.	CS	National Representative Survey of the U.S.	18+	ns	5,692	ns	Agoraphobia, alcohol dependence, alcohol abuse, attention deficit/hyperactivity disorder, bipolar disorder, conduct disorder, dysthymia, generalized anxiety disorder, intermittent explosive disorder, major depressive, obsessive-compulsive disorder, oppositional-defiant disorder, panic disorder, post-traumatic stress disorder, separation anxiety disorder, specific phobia, substance abuse, substance dependence	DSM-IV	First symptom
Kessler [87]	U.S.	CS	National Comorbidity Survey	15-54	ns	8,098	1,993	Alcohol abuse, alcohol dependence	DSM-IV	First diagnosis
Kessler [88]	Belgium, Brazil, Colombia, France, Germany, Italy, Mexico, Netherlands, New Zealand, Northern Ireland, Portugal, Romania, Spain, U.S.	CS	World Mental Health Survey	18+	ns	24,124	ns	Bulimia nervosa, binge eating disorder	DSM-IV	First diagnosis
Kessler [89]	Belgium, China, Colombia, France, Germany, Israel, Italy, Japan, Lebanon, Mexico, New Zealand, South Africa, Spain, The Netherlands, Ukraine, U.S.	CS	World Mental Health Survey	18+	ns	85,052	24,449	Anxiety disorders, impulse control disorders, mood disorder, substance use disorder	DSM-IV	First diagnosis
Kessler [90]	Belgium, Brazil, Colombia, Italy, Mexico, Netherlands, New Zealand, Northern Ireland, Portugal, Romania, Spain, U.S.	CS	World Mental Health Survey	18+	ns	23,653	656	Binge eating disorder, bulimia nervosa	DSM-IV	First diagnosis
Kessler[91]	U.S.	CS	National Comorbidity Survey Replication (NCS -R)	18+	NA	9,282	2006	Panic disorder	DSM-IV	First symptom
Kim [92]	South Korea	PC	CRESCEND	16+	ns	1,183	723	Dysthymia, major depressive disorde	DSM-IV	First symptom
Kim-Cohen [93]	New Zealand	BC	Dunedin Multidisciplinary Health and Development Study	0 to 26	48	1,037	253	Agoraphobia, alcohol dependence, anorexia nervosa, antisocial personality disorder, bipolar disorder, bulimia nervosa, cannabis dependence, dysthymia, eating disorder, generalized anxiety disorder, major depressive disorder, obsessive-compulsive disorder, panic disorder, post-traumatic stress disorder, social phobia, specific phobia, schizophreniform disorder substance abuse, substance dependence	DSM -III/-R	First diagnosis
Kirkbride [94]	UK	PC	Aetiology and ethnicity in Schizophrenia and other Psychoses	16 to 64	ns	1,631,442	568	Affective psychoses, non-affective psychoses, substance induced psychoses, schizophrenia	DSM-IV	First contact with the treatment service
Knappe [95]	Germany	BC	Early Developmental Stages of Psychopathology Study	14-34	49.3	3,021	628	Social phobia	DSM-IV	First symptom
Kohler [96]	Scotland	PC	Psychiatric service of Dumfries and Galloway	14+	ns	147,000	463	Early onset psychosis, late onset psychosis, very late onset psychosis.	ICD-9/10	First contact with the treatment service
Korten [97]	Netherlands	PC	Netherlands Study of Depression and Anxiety	18 to 73	ns	2,981	1,104	Major depressive disorder	DSM-IV	First symptom
Lahey [98]	U.S.	CS	National Institute of Mental Health Methods for the Epidemiology of Child and Adolescent Mental Disorders	9 to 17	ns	1,285	74	Conduct disorder	DSM-III-R	First symptom
Lahti [99]	Finland	BC	Helsinki Birth Cohort Study	0 to 76	47.9	13,243	1,682	Agoraphobia, bipolar disorder, dysthymia, generalized anxiety disorder, major depressive	ICD-8/9/10	First hospitalization

Author	Country	Design	Study	Age	Female %	Population	Individuals	Disorders	Diagnostic criteria	Onset definition
								disorder, non-psychotic substance use disorder, obsessive-compulsive disorder, panic disorder, personality disorders, post-traumatic stress disorder, psychotic mood disorder, psychotic substance use disorder, non-psychotic mood disorder, schizophrenia spectrum, separation anxiety disorder, specific phobia, substance use disorder		
Le Strat [100]	U.S.	CS	National Epidemiologic Survey on Alcohol and Related Conditions	18+	Ns	43,093	4,696	Alcohol dependence	DSM-IV	First diagnosis
Lee [101]	Brazil, Bulgaria, Colombia, India, Lebanon, Mexico, New Zealand, Romania, USA	CS	World Mental Health Survey	18+	Ns	28,988	ns	Bipolar disorder	DSM-IV	First symptom
Lee [102]	China	CS	Survey from Beijing and Shanghai	19 to 70	ns	5,201	ns	Alcohol abuse, alcohol dependence, bipolar disorder, dysthymia, generalized anxiety disorder, intermittent explosive disorder, obsessive-compulsive disorder, panic disorder, post-traumatic stress disorder, specific phobia	ICD-10	First symptom
Lee [103]	China	CS	Survey from Beijing and Shanghai	18+	ns	5,201	43	Major depressive disorder	ICD-10, DSM-IV	First symptom
Lee[104]	Belgium, France, Germany, Israel, Italy, Japan, the Netherlands, New Zealand, Spain, U.S., China (Beijing, Shanghai), Columbia, Lebanon, Mexico, Nigeria, South Africa, Ukraine	CS	World Mental Health Survey	16+	ns	85,052	4,842	Generalized anxiety disorder	DSM-IV	First symptom
Lepine[105]	France	CS	French Cohort	18+	ns	1,787	60	Social phobia	DSM-III	ns
Lepine[106]	France	CS	French Cohort	18+	ns	1,787	52	Agoraphobia	DSM-III	ns
Levine [107]	Israel	PC	The Israeli National Psychiatric Case Registry	18+	ns	12,071	8,419	Schizophrenia	ICD-9	First hospitalization
Levinson [108]	Israel	CS	National population register from Israeli survey	21+	ns	4,859	860	Bipolar disorder, dysthymia, generalized anxiety disorder, major depressive disorder, panic disorder, post-traumatic stress disorder	ICD-10, DSM-IV	First symptom
Lewis [109]	U.S.	CS	National Institute of Mental Health Epidemiologic Catchment Area study	18+	ns	9,868	1,020	Alcohol abuse, alcohol dependence	DSM-III	First symptoms
Luoma [110]	Finland	BC	Northern Finland 1966 Birth Cohort	0-31	34.7	98	98	Schizophrenia	DSM-III-R	First symptom
Manetti [111]	U.S.	CS	National Epidemiologic Survey on Alcohol and Related Conditions	0 to 99	ns	43,093	3,199	Major depressive disorder	DSM-IV	First diagnosis
Mattisson [112]	Sweden	CS	Lundby Cohort	12+	ns	3,563	348	Alcohol use disorder	DSM-IV	First symptom
McEvoy [113]	Australia	CS	Australian National Survey of Mental Health and Wellbeing	16 to 85	ns	8,841	1,045	Agoraphobia, generalized anxiety disorder, obsessive-compulsive disorder, panic disorder, post-traumatic stress disorder, social phobia, substance use disorder	DSM-IV	First diagnosis
McLaughlin [114]	U.S.	CS	National Comorbidity Survey-Adolescent Supplement	13-18	Ns	6,483	787	Intermittent explosive disorder	DSM-IV	First symptom
Medina-Mora [115]	Mexico	CS	World Mental Health Survey	18 to 65	ns	2,362	1,148	Agoraphobias, alcohol abuse, alcohol dependence, attention deficit hyperactivity disorder, bipolar disorder, conduct disorder, dysthymia, generalized anxiety disorder, major depressive disorder, oppositional defiant disorder, panic disorder, post-traumatic stress disorder, separation anxiety disorder, social phobia, specific phobias, substance	DSM-IV	First diagnosis

Author	Country	Design	Study	Age	Female %	Population	Individuals	Disorders	Diagnostic criteria	Onset definition
								abuse		
Merikangas [116]	U.S.	CS	National Comorbidity Survey-Adolescent Supplement	13-18	51	10,123	7,815	Anxiety disorders, behavior disorder, mood disorder, substance use disorder	DSM-IV	Ns
Merikangas[117]	U.S.	CS	National Comorbidity Survey Replication	18+	ns	9,282	826	Bipolar disorder	DSM-IV	First symptom
Mojtabai [118]	U.S.	CS	National Epidemiologic Survey on Alcohol and Related Conditions	18+	58	34,653	3,006	Major depressive episode	DSM-IV	First diagnosis
Mojtabai[119]	U.S.	CS	National Comorbidity Survey	15 to 54	ns	5,877	995	Major depressive disorder	DSM-III	First diagnosis
Murphy [120]	Australia	CS	Australian National Survey of Mental Health and Wellbeing	16-85	Ns	8,841	1,365	Major depressive disorder	DSM-IV	ns
Navarro-Mateu [121]	Spain	CS	PEGASUS	18+	49,5	2,621	115	Bipolar disorder, generalized anxiety disorder, major depressive disorder, post-traumatic stress disorder, social phobia, specific phobia, substance abuse	DSM-IV	First symptom
Negash [122]	Ethiopia	CS	Survey from Butajira	15 to 49	ns	2,880	292	Bipolar disorder	DSM-IV	First symptom
Nelson [123]	U.S.	CS	U.S. National Comorbidity Survey	15 to 54	ns	4,011	1,091	Alcohol dependence	DSM-III-R	First symptom
Nesvag [124]	Norway	CS	Norwegian Patient Registry and the Norwegian Prescription Database	0 to 18	49	1,179,368	443	Bipolar disorder, major depressive disorder with psychotic symptoms, schizophrenia spectrum disorder	ICD-10	First contact with the treatment service
Nordström [125]	Finland	BC	Northern Finland Birth Cohort	0 to 15	ns	9,432	135	Attention deficit/hyperactivity disorder, disruptive behaviour disorder	ICD-9/10	First hospitalization
Oakley Browne [126]	New Zeland	CS	The New Zealand Mental Health Survey	16+	ns	4,831	ns	Agoraphobia, alcohol dependence, alcohol abuse, anorexia nervosa, bipolar disorder, bulimia nervosa, dysthymia, generalized anxiety disorder, major depressive disorder, obsessive-compulsive disorder, panic disorder, post-traumatic stress disorder, social phobia, specific phobia, substance abuse, substance dependence	DSM-IV	First symptom
Ormel [127]	Netherlands	CS	The Tracking Adolescents' Individual Lives Survey	0 to 19	ns	1,584	1,048	Agoraphobia, attention deficit/hyperactivity disorder, alcohol abuse, alcohol dependence, bipolar disorder, conduct disorder, dysthymia, generalized anxiety disorder, major depressive disorder, obsessive-compulsive disorder, oppositional-defiant disorder, panic disorder, specific phobia, separation anxiety disorder, substance abuse, substance dependence	DSM-IV	First symptom
Orvaschel [128]	U.S.	CS	Oregon Adolescent Depression Project	14 to 18	53.7	1,701	46	Agoraphobia, alcohol use disorder, disruptive behavior disorder, dysthymia, generalized anxiety disorder, major depressive disorder, obsessive-compulsive disorder, panic disorder, post-traumatic stress disorder, separation anxiety disorder, social phobia, specific phobia, substance use disorder	DSM-III	First diagnosis
Pedersen [129]	Denmark	PC	Danish registries	0+	ns	5,600,000	320,543	Alcohol use, anorexia nervosa, bipolar disorder, cannabis use, childhood autism, depressive disorder, hyperkinetic disorder, obsessive-compulsive disorder, mental retardation, personality disorders, pervasive developmental disorders, schizoaffective disorder, schizophrenia spectrum disorders, somatoform disorders, stress-related disorder	ICD-10	First diagnosis
Perala [130]	Finland	CS	Health 2000 Health Examination Survey	30+	Ns	6,005	482	Alcohol dependence	DSM-IV	First symptom
Peralta [131]	Spain	PC	Treatment of first-episode drug-naïve patients.	15 to 65	ns	ns	122	Schizophrenia spectrum disorders	DSM-IV	First symptom,
Peyre [132]	U.S.	CS	National Epidemiologic Survey on Alcohol and Related Conditions	0 to 18	ns	34,653	764	Attention deficit/Hyperactivity disorder	DSM-IV	First diagnosis

Author	Country	Design	Study	Age	Female %	Population	Individuals	Disorders	Diagnostic criteria	Onset definition
Polo [133]	U.S.	CS	National Latino and Asian American Study, National Comorbidity Survey Replication	18+	56.5	6,601	1,601	Social Anxiety Disorder	DSM-IV	ns
Rabinowitz [134]	Israel	PC	National Psychiatric Hospitalization Case Registry	15+	40.4	10,756	10,756	Schizophrenia	ICD-9	First hospitalization
Ramage-Morin[135]	Canada		Canadian Community Health Survey: Mental Health and Well-Being	15+	ns	36,984	1,397	Panic disorder	DSM-IV	First diagnosis
Ran [136]	China	CS	Survey from Xinjin County Chengdu	15+	ns	149,231	510	Schizophrenia	ICD-10	First symptom
Ran[137]	China	CS	Xinjin County Survey	15+	ns	123,572	911	Unspecified non organic psychosis	ICD-10	First symptom
Rasanen[138]	Finland	BC	Northern Finland 1966 Birth Cohort	16 to 28	48.8	11,017	89	Schizophrenia	DSM-III	First symptom
Rastam [139]	Sweden	CS	ns	14,5 to 15,5	ns	4,291	20	Anorexia nervosa	DSM-III	First diagnosis
Rautio [140]	Finland	CS	NFBC 1966 Study	16 to 45	ns	10,277	161	Schizophrenia spectrum	ICD-8/9/10	First diagnosis
Reardon[141]	U.S.	CS	Colorado Social Health Survey (CSHS)	18+	57	4,730	785	Alcohol abuse, alcohol dependence, antisocial personality disorder, bipolar disorder, dysthymia, general anxiety disorder, major depressive disorder, obsessive-compulsive disorder, panic disorder, phobias, schizophrenia, substance abuse, substance dependence	DSM-III	First symptom
Reed [142]	Germany	CS	Early Developmental Stages of Psychopathology	14-24	50.6	ns	3,021	Panic disorder	DSM-IV	First diagnosis
Rey [143]	U.S.	CS	Epidemiologic Catchment Area	18+	ns	19,182	6,138	Alcohol abuse, alcohol dependence, antisocial personality disorder, bipolar disorder, generalised anxiety disorder, obsessive-compulsive disorder, phobias, somatisation, substance abuse, substance dependence	DSM-III	First diagnosis
Riala[144]	Finland	CS	Northern Finland 1966 Birth Cohort	16+	47.4	10,934	470	Adjustment disorders, atypical psychoses, bipolar disorder, brief psychotic episode, delusional disorders, depression with psychotic symptoms, nonpsychotic disorder, personality disorders, schizoaffective disorder, schizophrenia, schizophreniform disorders, substance use disorders	DSM-III	First diagnosis
Ritter [145]	Germany	PC	Early Developmental Stages of Psychopathology Study	14 to 34	ns	1,943	41	Bipolar disorder, major depressive disorder	ICD-10	ns
Rodgers [146]	Switzerland	CS	Zurich study Sample	13 to 66	ns	591	68	Obsessive-compulsive disorder	DSM-IV	First diagnosis
Roest [147]	Multi-country	CS	World Mental Health Survey	18+	ns	136,357	2,045	Agoraphobia	DSM-V	First diagnosis
Ruscio [148]	Australia, Belgium, Brazil, Bulgaria, China, Colombia, France, Germany, Iraq, Israel, Italy, Japan, Lebanon, Mexico, New Zeland, Nigeria, Northern Ireland, Peru, Poland, Portugal, Romania, South Africa, The Netherlands, Ukraine, U.S.	CS	World Mental Health Survey	18+	ns	147,261	5,888	Generalized anxiety disorder	DSM-V	First diagnosis
Sala [149]	U.S.	CS	National Epidemiologic Survey on Alcohol and Related Conditions	18+	ns	34,653	1,528	Bipolar disorder	DSM-IV	First diagnosis
Schaffer [150]	Canada	CS	Canadian Community Health Survey: Mental Health and Well-Being	15+	ns	36,984	852	Bipolar disorder	DSM-IV	First diagnosis
Schneier [151]	U.S.	CS	National Epidemiological Survey on Alcohol AND Related Conditions	18+	57	43,093	2,970	Alcohol dependence, social anxiety disorder	DSM-IV	First diagnosis
Schneier[152]	U.S.	CS	Epidemiologic Catchment Area Study ECA	18+	ns	18,572	97	Social phobia	DSM-III	First diagnosis
Schuckit [153]	U.S.	CS	San Diego Prospective Study	18+	0	373	140	Alcohol abuse, alcohol dependence	DSM-III/-R	First diagnosis

Author	Country	Design	Study	Age	Female %	Population	Individuals	Disorders	Diagnostic criteria	Onset definition
Scott [154]	Brazil, Bulgaria, China, Colombia, Japan, Lebanon, Nigeria, Northern Ireland, Peru, Poland, Portugal, Romania, South Africa, Ukraine, U.S.	CS	World Mental Health Survey	18+	ns	88,063	705	Intermittent explosive disorder	DSM-IV	First diagnosis
Silove [155]	Belgium, France, Brazil, Bulgaria, China, Colombia, Germany, Italy, Lebanon, Mexico, Nigeria, Northern Ireland, Peru, Portugal, Romania, The Netherlands, Spain, U.S.	CS	World Mental Health Survey	18+	56.74	38,993	1,883	Separation anxiety disorder	DSM-IV	First diagnosis
Singh [156]	UK	CS	Conversion disoder in Nottingham survey	26 to 74	ns	37,000	18	Conversion disorder	DSM-III-R	First diagnosis
Slutske [157]	Australia	CS	Australian Twin Registry (ATR) Cohort II	0 to 43	57.08	4,663	128	Gambling disorder	DSM-IV	First symptom,
Sorenson [158]	U.S.	CS	Los Angeles Epidemiologic Catchment Area project	18 to 97	52.7	3,131	183	Major depressive disorder	DSM-III	First diagnosis
Stefansson [159]	Iceland	CS	Iceland Cohort	55 to 57	49	862	233	Alcohol abuse, alcohol dependence	DSM-III	First symptom
Stein [160]	Australia, Belgium, Brazil, Bulgaria, Colombia-Medellin, France, Germany,, Iraq, Italy, Japan, Lebanon, Mexico, New Zealand, Nigeria, Northern Ireland, People's Republic of China, Peru, Poland, Portugal, Romania, South Africa, Spain, The Netherlands, USA,, Ukraine	CS	World Health Organization World Mental Health Surveys initiative	18+	Ns	142,405	5,696	Social anxiety disorder	DSM-IV	ns
Stein[161]	South Africa	CS	SASH (South Africa Stress and Health Study)	18+	ns	4,351	1,290	Agoraphobia with or without panic, alcohol abuse, alcohol dependence, any anxiety disorder, generalized anxiety disorder, major depressive disorder, panic disorder, post-traumatic stress disorder, social phobia, substance abuse, substance dependence	DSM-IV, ICD-10	ns
Steinhausen [162]	Denmark	BC	Danish registry	0-18	48	68,982	7,893	Anxiety disorders, attention deficit/hyperactivity disorder, autism-spectrum disorders, conduct disorder, depressive disorder, eating disorder, obsessive-compulsive disorder, schizophrenia, substance use disorder, tic disorder	ICD-10	First diagnosis
Stinson, 2007 [163]	U.S.	CS	United States' National Epidemiologic Survey on Alcohol and Related Conditions	18+	ns	43,093	4,030	Specific phobias	DSM-IV	First diagnosis
Suliman [164]	South Africa	CS	South African Stress and Health	18+	Ns	4,351	609	Alcohol abuse, alcohol dependence	DSM-IV	First symptom
Suvisaari [165]	Finland	PC	Finnish Population Register	16-26	ns	5,645	5,645	Schizophrenia	ICD-8/9	First hospitalization
Suvisaari [166]	Finland	PC	Finnish patients from the health care registers	0+	49	35,720	15,892	Schizophrenia spectrum disorders	ICD-8/9, DSM-III-TR	First hospitalization
Svensson [167]	Sweden	BC	The Swedish Multi-Generation Register	0+	Ns	395,055	3,138	Schizophrenia	ICD-8/9/10	First hospitalization
Swendsen[168]	U.S.	CS	National Comorbidity Survey-Adolescent Supplement	0-18	Ns	10,123	1,541	Alcohol abuse, alcohol dependence, substance abuse, substance dependence	DSM-IV	First diagnosis
Tai [169]	Taiwan	CS	National Health Insurance Research Database	3-25	20.4	1,000,000	2,385	Attention deficit/Hyperactivity disorder	ICD-9	First diagnosis
Taylor [170]	U.S.	CS	National Survey of American Life	18+	ns	5,191	7	Anorexia nervosa, bulimia nervosa, binge eating disorder	DSM-IV-TR	First symptom
Thorup [171]	Denmark	PC	OPUS STUDY	18+	ns	ns	578	Schizophrenia spectrum disorders.	ICD-10	First symptom or First contact with the treatment service
Tibi[172]	The Netherlands	CS	Netherlands Study of Depression and Anxiety (NESDA)	18 to 65	69	2,981	507	Agoraphobia	DSM-IV	First symptom

Author	Country	Design	Study	Age	Female %	Population	Individuals	Disorders	Diagnostic criteria	Onset definition
Tolin [173]	U.S.	CS	Database of Individual who have contacted the researchers for information about compulsive hoarding	0+	ns	2,271	751	Compulsive disorder	DSM-IV	First contact with the treatment service
Udo [174]	U.S.	CS	National Epidemiologic Survey on Alcohol and Related Conditions-III (NESARC-III)	18+	ns	36,309	686	Anorexia nervosa, bulimia nervosa, binge eating disorder	DSM-V	First diagnosis
Ullman [175]	Israel	CS	Israel National Psychiatric Hospitalization Case Registry	18+	17.4	21,499	235	Affective disorders, schizophrenia	ICD-10	First hospitalization
Vaingankar [176]	Singapore	CS	Singapore mental health study	18+	51.5	6,616	865	Alcohol abuse, alcohol dependence, bipolar disorder, dysthymia, major depressive disorder, generalized anxiety disorder, obsessive-compulsive disorder	ICD-10	First symptom
Vande Voort[177]	U.S.	CS	National Health and Nutrition Examination Survey	12 to 15	Ns	1,894	163	Attention deficit/hyperactivity disorder	DSM-5	First diagnosis
Verdura Vizcaino [178]	U.S.	CS	United States' National Epidemiologic Survey on Alcohol and Related Conditions	18+	ns	43,093	166	Gambling disorder	DSM-IV	First diagnosis
Viana [179]	Brazil	CS	São Paulo Megacity Mental Health Survey	18+	ns	2,942	1,318	Agoraphobia, alcohol abuse, alcohol dependence, attention deficit/hyperactivity disorder, bipolar disorder, conduct disorder, sthymia, generalized anxiety disorder, intermittent explosive disorder, major depressive disorder, obsessive-compulsive disorder, oppositional-defiant disorder, panic disorder, post-traumatic stress disorder, separation anxiety disorder, social phobia, specific phobia, substance abuse, substance dependence	DSM-IV	First diagnosis
Vila- Rodriguez [180]	Spain	CS	ME2-MHT	18 to 65	ns	440,000	231	Schizophrenia	DSM-IV	First contact with the treatment service
Wardenaar [181]	Belgium, Brazil, Bulgaria, China, Colombia, France, Germany, Iraq, Italy, Japan, Lebanon, Mexico, New Zealand, Nigeria, Northern Ireland, Peru, Poland, Portugal, Romania, Spain, The Netherlands, U.S.	CS	World Mental Health Survey	18+	ns	124,902	9,243	Specific phobias	DSM-IV	First diagnosis
Weissman [182]	U.S.	CS	Epidemiologic catchment Area Study ECA	18+	ns	18,572	232	Bipolar disorder, major depressive disorder	DSM-III	First diagnosis
Weissman [183]	Canada, France, Italy, Korea, Lebanon, New Zealand, Puerto Rico, Taiwan, U.S., Germany	CS	Cross-National Collaborative Group	18-64	ns	44,877	2,670	Bipolar disorder, major depressive disorder	DSM-III	First diagnosis
Weissman [184]	Canada, Germany, New Zealand, U.S.	PC	National Comorbidity Survey/Munich Follow-up Study	18+	59.3	23,239	1,727	Major depressive disorder	DSM-III	First diagnosis
Weissman[185]	Puerto Rico, South Korea	CS	The Puerto Rico Study of Psychiatric Disorders; The Korean Epidemiologic Study of Mental Disorders	18+	53.6	6,613	817	Social Phobia	DSM-III	First symptom
Wells [186]	New Zeland	CS	New Zealand Mental Health Survey	16+	ns	1,320	189	Bipolar disorder	DSM-IV	First diagnosis
WHO [187]	Brazil, Canada, Germany, Mexico, Netherlands, Turkey, U.S.	CS	Cross-national comparisons of the prevalences and correlates of mental disorders. WHO International Consortium in Psychiatric Epidemiology.	18+	ns	29,644	10,110	Anxiety disorders, mood disorders, substance use disorders	DSM-III-R/IV	First diagnosis
Wiborg [188]	Germany	CS	Psychenet: the Hamburg Network for Mental Health	18+	ns	1,645	136	Somatoform disorder	ICD-10	First symptom
Williams[189]	Australia	CS	Geelong Osteoporosis Study (GOS)	24 to 98	0	1,540	961	Anxiety disorder, mood disorder, substance use disorder	DSM-IV	First symptom
Wittchen [190]	Germany	PC	Early Developmental Stages of Psychopathology Study	14-24	ns	3,021	538	Agoraphobia, panic attack, panic disorder	DSM-IV	First diagnosis
Wittchen[191]	Germany	BC	Early Developmental Stages of Psychopathology Study (EDSP)	14 to 24	ns	3,021	220	Generalized social phobia, non-generalized social phobia	DSM-IV	

Author	Country	Design	Study	Age	Female %	Population	Individuals	Disorders	Diagnostic criteria	Onset definition
Woo[192]	South Korea	CS	Korean Epidemiologic Catchment Area study (KECA), Korean Epidemiologic Catchment Area study replication (KECA-R), 2011 Korean Epidemiologic Catchment Area study (KECA-2011).	18+	ns	18,807	1,533	Major depressive disorder, panic disorder	DSM-IV	ns
Yin [193]	China	CS	Tianjin Mental Health Survey	18+	46.6	11,748	439	Agoraphobia, bipolar disorder, generalized anxiety disorder, major depressive disorder, obsessive-compulsive disorder, panic disorder, post-traumatic stress disorder, schizophrenia spectrum disorders, specific phobia, substance use disorder	DSM-IV	ns
Yoshimasu [194]	Japan	CS	World Mental Health Survey	18+	ns	4,134	80	Intermittent explosive disorder	DSM-IV	First diagnosis
Young [195]	U.S.	CS	Recruit Assessment Program	18+	0	65,178	6,128	Alcohol abuse	DSM-IV	First symptom
Zvolensky [196]	U.S.	CS	Colorado Social Health Survey	25+	52	4,744	ns	Cannabis dependence, panic disorder	DSM-III	First symptom

Legend. BC, birth cohort; CS, cross-sectional; DSM, diagnostic and statistical manual; ICD, international classification of diseases; PC, prospective cohort;

e-table 4. Studies excluded after full-text assessment, with reason for exclusion (k=734)

Author, year	Reason for exclusion
Azagba, 2019 [197]	No disorder defined according to established criteria
Baggio, 2013 [198]	No disorder defined according to established criteria
Best, 2001 [199]	No disorder defined according to established criteria
Cheng, 2011 [200]	No disorder defined according to established criteria
Cheng, 2018 [201]	No disorder defined according to established criteria
Ciairano, 2009 [202]	No disorder defined according to established criteria
De Graaf, 2010 [203]	No disorder defined according to established criteria
Demant, 2018 [204]	No disorder defined according to established criteria
Diekstra, 1993 [205]	No disorder defined according to established criteria
Duke, 2009 [206]	No disorder defined according to established criteria
Gonzalez-Chica, 2019 [207]	No disorder defined according to established criteria
Kaestle, 2015 [208]	No disorder defined according to established criteria
Kandel, 1992 [209]	No disorder defined according to established criteria
Karam, 2014 [210]	No disorder defined according to established criteria
Kessler, 2012 [211]	No disorder defined according to established criteria
Lahey, 1999 [212]	No disorder defined according to established criteria
Lintonen, 2000 [213]	No disorder defined according to established criteria
Lo, 2000 [214]	No disorder defined according to established criteria
Manna, 2010 [215]	No disorder defined according to established criteria
Martins-Oliveira, 2018 [216]	No disorder defined according to established criteria
McGrath, 2016 [217]	No disorder defined according to established criteria
Monshouwer, 2005 [218]	No disorder defined according to established criteria
Mutumba, 2019 [219]	No disorder defined according to established criteria
Najman, 2019 [220]	No disorder defined according to established criteria
Nigg, 2013 [221]	No disorder defined according to established criteria
Pacek, 2013 [222]	No disorder defined according to established criteria
Parra, 2003 [223]	No disorder defined according to established criteria
Reingle Gonzalez, 2016 [224]	No disorder defined according to established criteria
Resnick, 1997 [225]	No disorder defined according to established criteria
Roderick, 2018 [226]	No disorder defined according to established criteria
Soueif, 1998 [227]	No disorder defined according to established criteria
Staff, 2015 [228]	No disorder defined according to established criteria
Storr, 2004 [229]	No disorder defined according to established criteria
Strunin, 2017 [230]	No disorder defined according to established criteria
Uppal, 1977 [231]	No disorder defined according to established criteria
Van Der Vorst [232]	No disorder defined according to established criteria
Vieira, 2007 [233]	No disorder defined according to established criteria
Wallinius, 2016 [234]	No disorder defined according to established criteria
Wilson, 1994 [235]	No disorder defined according to established criteria
Fiestas, 2014 [236]	no English
Haro, 2006 [237]	no English
Paixao, 2009 [238]	no English
Abdin, 2013 [239]	no usable age at onset estimate
Aberg, 2016 [240]	no usable age at onset estimate
Acarturk, 2009[241]	no usable age at onset estimate
Afzali, 2017[242]	no usable age at onset estimate
Agosti, 2008 [243]	no usable age at onset estimate
Ahmed, 2010 [244]	no usable age at onset estimate
Alaräisänen, 2006 [245]	no usable age at onset estimate
Albor, 2017 [246]	no usable age at onset estimate
Alghzawi, 2018 [247]	no usable age at onset estimate
Allen, 2014 [248]	no usable age at onset estimate
Alonso, 2014 [249]	no usable age at onset estimate
Andrade, 1996 [250]	no usable age at onset estimate
Andreasen, 2005 [251]	no usable age at onset estimate
Angst, 1998[252]	no usable age at onset estimate
Angst, 2009 [253]	no usable age at onset estimate
Angst, 2012 [254]	no usable age at onset estimate
Angst, 2015 [255]	no usable age at onset estimate
Bacon, 2009 [256]	no usable age at onset estimate
Baldwin, 2014 [257]	no usable age at onset estimate
Barker, 2008 [258]	no usable age at onset estimate

Author, year	Reason for exclusion
Bauer, 2017 [259]	no usable age at onset estimate
Bauermeister, 2011 [260]	no usable age at onset estimate
Baumeister, 2005 [261]	no usable age at onset estimate
Behrendt, 2009 [262]	no usable age at onset estimate
Behrendt, 2012 [263]	no usable age at onset estimate
Belik, 2008 [264]	no usable age at onset estimate
Bernstein, 2006 [265]	no usable age at onset estimate
Bhugra, 2002 [266]	no usable age at onset estimate
Bilevicius, 2019 [267]	no usable age at onset estimate
Birrell, 2015 [268]	no usable age at onset estimate
Blobaum, 2006 [269]	no usable age at onset estimate
Bogren, 2007 [270]	no usable age at onset estimate
Bogren, 2010 [271]	no usable age at onset estimate
Borga, 1992 [272]	no usable age at onset estimate
Borges, 2011 [273]	no usable age at onset estimate
Boschloo, 2011 [274]	no usable age at onset estimate
Bourdon, 1992 [275]	no usable age at onset estimate
Breslau, 2004 [276]	no usable age at onset estimate
Breslau, 2007 [277]	no usable age at onset estimate
Breslau, 2009 [278]	no usable age at onset estimate
Brezo, 2007 [279]	no usable age at onset estimate
Bromet, 2007 [280]	no usable age at onset estimate
Brower, 2010 [281]	no usable age at onset estimate
Browne, 2006 [282]	no usable age at onset estimate
Bruffaerts, 2007 [283]	no usable age at onset estimate
Bruffaerts, 2017 [284]	no usable age at onset estimate
Bulayeva, 2006 [285]	no usable age at onset estimate
Buller, 1991 [286]	no usable age at onset estimate
Burke, 1991 [287]	no usable age at onset estimate
Burke, 1994 [288]	no usable age at onset estimate
Burns, 1980 [289]	no usable age at onset estimate
Burt, 2010 [290]	no usable age at onset estimate
Byers, 2014 [291]	no usable age at onset estimate
Cantor-Graae, 2007 [292]	no usable age at onset estimate
Capone, 2008 [293]	no usable age at onset estimate
Castagnini, 2013 [294]	no usable age at onset estimate
Chan, 2010 [295]	no usable age at onset estimate
Chang, 2010 [296]	no usable age at onset estimate
Chapa, 2018 [297]	no usable age at onset estimate
Chapman, 2015[298]	no usable age at onset estimate
Chartier, 2003[299]	no usable age at onset estimate
Chartier, 2011 [300]	no usable age at onset estimate
Chen, 2009 [301]	no usable age at onset estimate
Cheng, 2018 [302]	no usable age at onset estimate
Chong, 2012 [303]	no usable age at onset estimate
Clark, 2007 [304]	no usable age at onset estimate
Clemmensen, 2016 [305]	no usable age at onset estimate
Cohen, 2016 [306]	no usable age at onset estimate
Colman, 2007 [307]	no usable age at onset estimate
Crum, 2004 [308]	no usable age at onset estimate
Cuijpers, 2007 [309]	no usable age at onset estimate
David,1997 [310]	no usable age at onset estimate
Dayal, 2017 [311]	no usable age at onset estimate
de Graaf, 2011 [312]	no usable age at onset estimate
de Heer, 2017 [313]	no usable age at onset estimate
Degenhardt, 2003 [314]	no usable age at onset estimate
Degenhardt, 2009 [315]	no usable age at onset estimate
Degonda, 1993 [316]	no usable age at onset estimate
Demallie, 1995 [317]	no usable age at onset estimate
Devanand, 2014 [318]	no usable age at onset estimate
Dietrich, 2009 [319]	no usable age at onset estimate
Ding, 2009 [320]	no usable age at onset estimate
Doherty, 2008 [321]	no usable age at onset estimate
Dube, 2006 [322]	no usable age at onset estimate

**Author, year**	**Reason for exclusion**
Ehlers, 2006 [323]	no usable age at onset estimate
Essau, 2010 [324]	no usable age at onset estimate
Evans, 2012 [325]	no usable age at onset estimate
Fairman, 2019 [326]	no usable age at onset estimate
Fallu, 2014 [327]	no usable age at onset estimate
Farrer, 1989 [328]	no usable age at onset estimate
Fergusson, 2013 [329]	no usable age at onset estimate
Fergusson, 2014 [330]	no usable age at onset estimate
Fischer, 2015 [331]	no usable age at onset estimate
Fleming, 1989 [332]	no usable age at onset estimate
Fontenelle, 2011 [333]	no usable age at onset estimate
French, 2009 [334]	no usable age at onset estimate
Gaysina, 2011 [335]	no usable age at onset estimate
Gilder, 2014 [336]	no usable age at onset estimate
Gissler, 2013 [337]	no usable age at onset estimate
Glantz, 2009 [338]	no usable age at onset estimate
Glazebrook, 1997 [339]	no usable age at onset estimate
Glenn, 2017 [340]	no usable age at onset estimate
Godart, 2012 [341]	no usable age at onset estimate
Goldstein, 2006 [342]	no usable age at onset estimate
Gonzalez-Blanch, 2008 [343]	no usable age at onset estimate
Goodwin, 2001 [344]	no usable age at onset estimate
Grant, 2006 [345]	no usable age at onset estimate
Gratzer, 2004 [346]	no usable age at onset estimate
Greenfield, 2010 [347]	no usable age at onset estimate
Gregory, 2009 [348]	no usable age at onset estimate
Grucza, 2008 [349]	no usable age at onset estimate
Gureje, 2011 [350]	no usable age at onset estimate
Hafner, 1991 [351]	no usable age at onset estimate
Hafner, 1999 [352]	no usable age at onset estimate
Hamzawi, 2012 [353]	no usable age at onset estimate
Hanna, 1999 [354]	no usable age at onset estimate
Hanna, 2001 [355]	no usable age at onset estimate
Hansen, 2009 [356]	no usable age at onset estimate
Harford, 2001 [357]	no usable age at onset estimate
Harford, 2006 [358]	no usable age at onset estimate
Hart, 2012 [359]	no usable age at onset estimate
Hastrup, 2018 [360]	no usable age at onset estimate
Hayatbakhsh, 2013 [361]	no usable age at onset estimate
Hayatbakhsh, 2014 [362]	no usable age at onset estimate
Helenius, 2012 [363]	no usable age at onset estimate
Hermos, 2008 [364]	no usable age at onset estimate
Hill, 2000 [365]	no usable age at onset estimate
Hingson, 2009 [366]	no usable age at onset estimate
Holdcraft, 2004 [367]	no usable age at onset estimate
Hong, 2004 [368]	no usable age at onset estimate
Horwath, 2000 [369]	no usable age at onset estimate
Horwath, 1992[370]	no usable age at onset estimate
Horwath, 1993 [371]	no usable age at onset estimate
Hwu, 1996 [372]	no usable age at onset estimate
Hwu, 2005 [373]	no usable age at onset estimate
Janzing, 2009 [374]	no usable age at onset estimate
Jenkins, 2011 [375]	no usable age at onset estimate
Joinson, 2013 [376]	no usable age at onset estimate
Kaelber, 2018 [377]	no usable age at onset estimate
Kaltiala-Heino, 2003 [378]	no usable age at onset estimate
Karam, 2012 [379]	no usable age at onset estimate
Keenan, 2010 [380]	no usable age at onset estimate
Keers, 2013 [381]	no usable age at onset estimate
Keller, 1992 [382]	no usable age at onset estimate
Kelly, 2012 [383]	no usable age at onset estimate
Kerr, 2015 [384]	no usable age at onset estimate
Kessing, 1998 [385]	no usable age at onset estimate
Kessler, 1993 [386]	no usable age at onset estimate

**Author, year**	**Reason for exclusion**
Kessler, 1995 [387]	no usable age at onset estimate
Kessler, 1998 [388]	no usable age at onset estimate
Kessler, 1998 [389]	no usable age at onset estimate
Kessler, 1999 [390]	no usable age at onset estimate
Kessler, 2001 [391]	no usable age at onset estimate
Kessler, 2008 [392]	no usable age at onset estimate
Nock, 2009 [393]	no usable age at onset estimate
Kessler, 2015 [394]	no usable age at onset estimate
Keyes, 2016 [395]	no usable age at onset estimate
Khalifa, 2005 [396]	no usable age at onset estimate
Kim, 2016 [397]	no usable age at onset estimate
Kinnunen, 2002 [398]	no usable age at onset estimate
Kinnunen, 2018 [399]	no usable age at onset estimate
Kirkpatrick, 2000 [400]	no usable age at onset estimate
Knappe, 2009 [401]	no usable age at onset estimate
Kollins, 2005 [402]	no usable age at onset estimate
Konings, 2007 [403]	no usable age at onset estimate
Kratzer, 1997 [404]	no usable age at onset estimate
Kraus, 2007 [405]	no usable age at onset estimate
Krueger, 1996 [406]	no usable age at onset estimate
Kunert, 2010 [407]	no usable age at onset estimate
Laursen, 2011[408]	no usable age at onset estimate
Le Strat, 2015 [409]	no usable age at onset estimate
Leach, 2012 [410]	no usable age at onset estimate
Leao, 2005 [411]	no usable age at onset estimate
Leatherdale, 2008 [412]	no usable age at onset estimate
Lee, 2010 [413]	no usable age at onset estimate
Legleye, 2010 [414]	no usable age at onset estimate
Leung, 2013 [415]	no usable age at onset estimate
Lever-van Milligen, 2014 [416]	no usable age at onset estimate
Levinson, 2009 [417]	no usable age at onset estimate
Lewinsohn, 1986 [418]	no usable age at onset estimate
Lewinsohn, 1993 [419]	no usable age at onset estimate
Lieb, 2002 [420]	no usable age at onset estimate
Locatelli, 2017 [421]	no usable age at onset estimate
Loffler, 2009 [422]	no usable age at onset estimate
Lyness, 1994 [423]	no usable age at onset estimate
Lyvers, 2012 [424]	no usable age at onset estimate
Malmberg, 2010 [425]	no usable age at onset estimate
Marcelis, 1998 [426]	no usable age at onset estimate
Marmorstein, 2011 [427]	no usable age at onset estimate
Matza, 2003 [428]	no usable age at onset estimate
McCabe, 2006 [429]	no usable age at onset estimate
McCabe, 2016 [430]	no usable age at onset estimate
McIntyre, 2006 [431]	no usable age at onset estimate
McKeganey, 2005 [432]	no usable age at onset estimate
McLaughlin, 2010 [433]	no usable age at onset estimate
McLean, 2011 [434]	no usable age at onset estimate
Meier, 2013 [435]	no usable age at onset estimate
Melchior, 2007 [436]	no usable age at onset estimate
Mikkelsen, 2014 [437]	no usable age at onset estimate
Miller, 2002 [438]	no usable age at onset estimate
Milne, 2009 [439]	no usable age at onset estimate
Mok, 2016 [440]	no usable age at onset estimate
Monshouwer, 2012 [441]	no usable age at onset estimate
Montgomery, 1999 [442]	no usable age at onset estimate
Morean, 2014 [443]	no usable age at onset estimate
Moss, 2010 [444]	no usable age at onset estimate
Mota, 2010 [445]	no usable age at onset estimate
Munkholm, 2016 [446]	no usable age at onset estimate
Mustelin, 2015 [447]	no usable age at onset estimate
Mustonen, 2018 [448]	no usable age at onset estimate
Myles-Worsley, 2007 [449]	no usable age at onset estimate
Neale, 1989 [450]	no usable age at onset estimate

Author, year	Reason for exclusion
Nesvaag, 2017 [451]	no usable age at onset estimate
Nettelbladt, 2009 [452]	no usable age at onset estimate
Newman, 1988 [453]	no usable age at onset estimate
Nierenberg, 2011 [454]	no usable age at onset estimate
Nishiura, 2017 [455]	no usable age at onset estimate
Nixon, 2008 [456]	no usable age at onset estimate
Nock, 2006 [457]	no usable age at onset estimate
Nock, 2013 [458]	no usable age at onset estimate
Nolen, 2013 [459]	no usable age at onset estimate
Oakley Browne, 2009 [460]	no usable age at onset estimate
Odgers, 2007 [461]	no usable age at onset estimate
Omer, 2013 [462]	no usable age at onset estimate
O'Neil, 2016 [463]	no usable age at onset estimate
Onyike, 2013 [464]	no usable age at onset estimate
Osler, 2008 [465]	no usable age at onset estimate
Paananen, 2013 [466]	no usable age at onset estimate
Pacheco, 2010 [467]	no usable age at onset estimate
Pardo, 2007 [468]	no usable age at onset estimate
Parker, 2004 [469]	no usable age at onset estimate
Pasco, 2010 [470]	no usable age at onset estimate
Paykel, 2006[471]	no usable age at onset estimate
Pedersen, 2013 [472]	no usable age at onset estimate
Perroud, 2010 [473]	no usable age at onset estimate
Pfister, 2007[474]	no usable age at onset estimate
Pilatti, 2014 [475]	no usable age at onset estimate
Pitkanen, 2005 [476]	no usable age at onset estimate
Pitkanen, 2008 [477]	no usable age at onset estimate
Platt, 2019 [478]	no usable age at onset estimate
Polanczyk, 2010 [479]	no usable age at onset estimate
Pope, 2014 [480]	no usable age at onset estimate
Posada, 2009 [481]	no usable age at onset estimate
Power, 2013 [482]	no usable age at onset estimate
Prelipceanu, 2012 [483]	no usable age at onset estimate
Rabinowitz, 2006 [484]	no usable age at onset estimate
Ran, 2003 [485]	no usable age at onset estimate
Rasanen, 1998 [486]	no usable age at onset estimate
Ratnasingham, 2013 [487]	no usable age at onset estimate
Razavi, 2015 [488]	no usable age at onset estimate
Reichenberg, 2006 [489]	no usable age at onset estimate
Resnick, 1997 [490]	no usable age at onset estimate
Reuter, 2007 [491]	no usable age at onset estimate
Ridenour, 2011 [492]	no usable age at onset estimate
Roberts, 2008 [493]	no usable age at onset estimate
Rossler, 2012 [494]	no usable age at onset estimate
Rubio, 2014 [495]	no usable age at onset estimate
Sanchez, 2013 [496]	no usable age at onset estimate
Savolainen, 2012 [497]	no usable age at onset estimate
Schneider, 2016 [498]	no usable age at onset estimate
Schoeyen, 2011 [499]	no usable age at onset estimate
Scocco, 2008 [500]	no usable age at onset estimate
Scott, 2008 [501]	no usable age at onset estimate
Scott, 2011 [502]	no usable age at onset estimate
Scott, 2013 [503]	no usable age at onset estimate
Seedat, 2009 [504]	no usable age at onset estimate
Shaffer, 1996 [505]	no usable age at onset estimate
Shang, 2017 [506]	no usable age at onset estimate
Shillington, 2000 [507]	no usable age at onset estimate
Shillington, 2011 [508]	no usable age at onset estimate
Silberg, 2015 [509]	no usable age at onset estimate
Silver, 2005 [510]	no usable age at onset estimate
Simon, 1992 [511]	no usable age at onset estimate
Skodol, 2011 [512]	no usable age at onset estimate
Skodol, 2014 [513]	no usable age at onset estimate
Smart, 2007 [514]	no usable age at onset estimate

Author, year	Reason for exclusion
Smyth, 2011 [515]	no usable age at onset estimate
Sorensen, 2016[516]	no usable age at onset estimate
Sourander, 2007[517]	no usable age at onset estimate
Spaner, 1994 [518]	no usable age at onset estimate
Spauwen, 2003 [519]	no usable age at onset estimate
Spiers, 2011 [520]	no usable age at onset estimate
Stein, 2008 [521]	no usable age at onset estimate
Stein, 2010 [522]	no usable age at onset estimate
Stevens, 2012 [523]	no usable age at onset estimate
Stice, 1998 [524]	no usable age at onset estimate
Strunin, 2007 [525]	no usable age at onset estimate
Strunin, 2013 [526]	no usable age at onset estimate
Swartz, 2005 [527]	no usable age at onset estimate
Tanskanen,2008 [528]	no usable age at onset estimate
Tantoh, 2016 [529]	no usable age at onset estimate
Tebeka, 2018 [530]	no usable age at onset estimate
Tegethoff, 2016 [531]	no usable age at onset estimate
Ten Have, 2002 [532]	no usable age at onset estimate
Ten Have, 2002 [533]	no usable age at onset estimate
Ten Have, 2013 [534]	no usable age at onset estimate
Ten Have, 2013 [535]	no usable age at onset estimate
Ten Have, 2013[536]	no usable age at onset estimate
Thapar, 2013 [537]	no usable age at onset estimate
Thomas, 2000 [538]	no usable age at onset estimate
Thompson, 2012 [539]	no usable age at onset estimate
Riekki, 2019 [540]	no usable age at onset estimate
Trinkoff, 1990 [541]	no usable age at onset estimate
Trumpf, 2010 [542]	no usable age at onset estimate
Udo, 2019 [543]	no usable age at onset estimate
Vaillant, 1994 [544]	no usable age at onset estimate
van Balkom, 2017 [545]	no usable age at onset estimate
van Beek, 2010 [546]	no usable age at onset estimate
van Lang, 2007 [547]	no usable age at onset estimate
van Os, 2002 [548]	no usable age at onset estimate
van Ours, 2006 [549]	no usable age at onset estimate
Vega, 2002 [550]	no usable age at onset estimate
Verhagen, 2008 [551]	no usable age at onset estimate
Verster, 2009 [552]	no usable age at onset estimate
Viana, 2018[553]	no usable age at onset estimate
Vilalta-Franch, 2013 [554]	no usable age at onset estimate
Vilsaint, 2019 [555]	no usable age at onset estimate
Vitola, 2017 [556]	no usable age at onset estimate
Vitousek, 1994 [557]	no usable age at onset estimate
Korff, 1985 [558]	no usable age at onset estimate
Walker, 2002 [559]	no usable age at onset estimate
Wang, 2005 [560]	no usable age at onset estimate
Wang, 2016 [561]	no usable age at onset estimate
Warner, 2007 [562]	no usable age at onset estimate
Weitzman, 2005 [563]	no usable age at onset estimate
Wells, 2008 [564]	no usable age at onset estimate
Wells, 2009 [565]	no usable age at onset estimate
Wessely,1994 [566]	no usable age at onset estimate
Willoughby, 2009 [567]	no usable age at onset estimate
Wilson, 1994 [568]	no usable age at onset estimate
Wilson, 2014 [569]	no usable age at onset estimate
Wisniewski, 2006 [570]	no usable age at onset estimate
Wittchen, 1992 [571]	no usable age at onset estimate
Wittchen, 2008 [572]	no usable age at onset estimate
Wright, 2009 [573]	no usable age at onset estimate
Wrobel, 1992 [574]	no usable age at onset estimate
Wu, 2014 [575]	no usable age at onset estimate
Yates, 2010 [576]	no usable age at onset estimate
Yucun, 1998 [577]	no usable age at onset estimate
Zakrajsek, 2006 [578]	no usable age at onset estimate

Author, year	Reason for exclusion
Abbott, 1998 [579]	Not general population
Agrawal, 2014 [580]	Not general population
Akvardar, 2004 [581]	Not general population
Alda, 1996 [582]	Not general population
Allegri, 2013 [583]	Not general population
Almeida, 2002 [584]	Not general population
Anand, 2015 [585]	Not general population
Arunpongpaisal, 2013 [586]	Not general population
Baldessarini, 2010 [587]	Not general population
Bar, 2003 [588]	Not general population
Barnes, 2011 [589]	Not general population
Bauer, 2012 [590]	Not general population
Bauer, 2015 [591]	Not general population
Beekman, 2004 [592]	Not general population
Benazzi, 2008 [593]	Not general population
Borga, 1991 [594]	Not general population
Borga, 1992 [595]	Not general population
Bourne, 2015 [596]	Not general population
Brakoulias, 2017[597]	Not general population
Bueno, 2014 [598]	Not general population
Bulayeva, 2000 [599]	Not general population
Buoli, 2016 [600]	Not general population
Bureau, 2013 [601]	Not general population
Butwicka,, 2010 [602]	Not general population
Caamano-Isorna, 2008 [603]	Not general population
Cadenhead, 2009 [604]	Not general population
Capella, 2015 [605]	Not general population
Carter, 2011[606]	Not general population
Castle, 1993 [607]	Not general population
Chen, 2014 [608]	Not general population
Chen, 2018 [609]	Not general population
Chengappa, 2003 [610]	Not general population
Chou, 2013 [611]	Not general population
Cieslak, 2016 [612]	Not general population
Col, 2014 [613]	Not general population
Cong, 2012 [614]	Not general population
Corruble, 2008 [615]	Not general population
Coryell, 2013 [616]	Not general population
Cotton, 2013 [617]	Not general population
Cuttler, 2017 [618]	Not general population
Cuypere, 2015 [619]	Not general population
Dakanalis, 2016 [620]	Not general population
Das, Praveen, 2018 [621]	Not general population
De la Torre, 2012 [622]	Not general population
Del Pino-Gutierrez, 2015 [623]	Not general population
Dell'Osso, 2013 [624]	Not general population
Delucchi, 2011 [625]	Not general population
Dierkhising, 2013 [626]	Not general population
Docherty, 2017 [627]	Not general population
Dominguez, 2013 [628]	Not general population
Duivis,2012 [629]	Not general population
Eaton, 1992 [630]	Not general population
Ebejer, 2012 [631]	Not general population
Egeland, 1987 [632]	Not general population
Ehmann, 2014 [633]	Not general population
El Wasify, 2018 [634]	Not general population
El-Hadidy, 2014 [635]	Not general population
Elkins, 2007 [636]	Not general population
Enander, 2018 [637]	Not general population
Eriksson, 2011 [638]	Not general population
Evenson, 1993 [639]	Not general population
Fang, 2013 [640]	Not general population
Faraone, 1994 [641]	Not general population
Faravelli, 2008 [642]	Not general population

Author, year	Reason for exclusion
Fichter, 2016 [643]	Not general population
Fiedorowicz, 2009 [644]	Not general population
Fiedorowicz, 2012 [645]	Not general population
Fink, 2016 [646]	Not general population
Fontenelle, 2017 [647]	Not general population
Furberg, 2008 [648]	Not general population
Gadermann, 2012 [649]	Not general population
Gaebel, 2012 [650]	Not general population
Gallagher, 2010 [651]	Not general population
Gallagher, 2017 [652]	Not general population
Geske, 2015 [653]	Not general population
Ghisleni, 2015[654]	Not general population
Ghosh, 2016 [655]	Not general population
Giltay, 2011 [656]	Not general population
Glahn, 2009 [657]	Not general population
Glasheen, 2013 [658]	Not general population
Goes, 2012 [659]	Not general population
Goldstein, 1990 [660]	Not general population
Goodwin, 2002 [661]	Not general population
Gradus, 2014 [662]	Not general population
Grant, 1998 [663]	Not general population
Grant, 2017 [664]	Not general population
Green, 2012 [665]	Not general population
Grigoroiu-Serbanescu, 2015 [666]	Not general population
Grove, 2012 [667]	Not general population
Gundogdu, 2013 [668]	Not general population
Gupta, 2017 [669]	Not general population
Gur, 2016 [670]	Not general population
Gureje, 1991 [671]	Not general population
Hafner, 1991 [672]	Not general population
Hafner, 1993 [673]	Not general population
Hakko, 2003 [674]	Not general population
Hall, 1991 [675]	Not general population
Hambrecht, 1992 [676]	Not general population
Hasler, 2016 [677]	Not general population
Hayatbakhsh, 2008 [678]	Not general population
Heinrich, 2013 [679]	Not general population
Hickling, 2018 [680]	Not general population
Hirneth, 2015 [681]	Not general population
Hoffmann, 2003 [682]	Not general population
Hsu, 2015 [683]	Not general population
Hulkko, 2017 [684]	Not general population
Hybels, 2012 [685]	Not general population
Ifabumuyi, 1985 [686]	Not general population
Iga, 2015 [687]	Not general population
Jaaskelainen, 2016 [688]	Not general population
Javaras, 2008 [689]	Not general population
Jenkins, 2018 [690]	Not general population
Jhanda, 2018 [691]	Not general population
Jia, 2015 [692]	Not general population
Jimenez-Murcia, 2010 [693]	Not general population
Jimenez-Murcia, 2016 [694]	Not general population
Jimenez-Murcia, 2017 [695]	Not general population
Johnson, 1985 [696]	Not general population
Joshi, 2001 [697]	Not general population
Kamali, 2009 [698]	Not general population
Katerberg, 2010 [699]	Not general population
Kecmanovic, 2010 [700]	Not general population
Kelly, 2013 [701]	Not general population
Kenardy, 1990 [702]	Not general population
Kendler, 1992 [703]	Not general population
Kendler, 2005 [704]	Not general population
Kendler, 2007 [705]	Not general population
Kendler, 2008 [706]	Not general population

Author, year	Reason for exclusion
Kendler, 2008 [707]	Not general population
Kendler, 2013 [708]	Not general population
Kessler, 2014 [709]	Not general population
Ketter, 2015 [710]	Not general population
Kienzle, 2009 [711]	Not general population
Kiezebrink, 2009 [712]	Not general population
Kinasz, 2016 [713]	Not general population
Kovacs, 1984 [714]	Not general population
Krausz, 1993 [715]	Not general population
Kuo, 2006 [716]	Not general population
Kuo, 2014 [717]	Not general population
Larson, 1974 [718]	Not general population
Lavori, 1987 [719]	Not general population
Lavori, 1993 [720]	Not general population
Lee, 2012 [721]	Not general population
Lesch, 2013 [722]	Not general population
Lewitzka, 2010 [723]	Not general population
Li, 2015 [724]	Not general population
Li, 2015[725]	Not general population
Li, 2017 [726]	Not general population
Lieb, 2007 [727]	Not general population
Lin, 2001 [728]	Not general population
Lin, 2013 [729]	Not general population
Liu, 2004 [730]	Not general population
Liu, 2015 [731]	Not general population
Lydecker, 2018 [732]	Not general population
Lynskey, 2007 [733]	Not general population
Lynskey, 2012 [734]	Not general population
Macciardi, 2007 [735]	Not general population
Maki, 2014 [736]	Not general population
Mäkikyrö, 1997 [737]	Not general population
Mann, 2013 [738]	Not general population
Marel, 2019 [739]	Not general population
Marie, 2008 [740]	Not general population
Markkula, 2017 [741]	Not general population
Masmoudi, 2016 [742]	Not general population
Mechri, 2013 [743]	Not general population
Menezes, 1993 [744]	Not general population
Moore, 2012 [745]	Not general population
Mork, 2013[746]	Not general population
Moss, 2008 [747]	Not general population
Mustelin, 2016 [748]	Not general population
Myles-Worsley, 1999 [749]	Not general population
Nair, 2013 [750]	Not general population
Naji, 2017 [751]	Not general population
Neuman, 2005 [752]	Not general population
Newman, 1998 [753]	Not general population
Niles, 2012 [754]	Not general population
Ohaeri, 1992 [755]	Not general population
Oncel, 2014 [756]	Not general population
Oostervink, 2015 [757]	Not general population
Ortiz-Garcia de la Foz, 2016 [758]	Not general population
Orvaschel, 1982 [759]	Not general population
Oude Voshaar, 2011 [760]	Not general population
Park, 2011 [761]	Not general population
Park, 2014 [762]	Not general population
Parker, 2013 [763]	Not general population
Paruk, 2015 [764]	Not general population
Pawlak, 2018 [765]	Not general population
Pedersen, 2001 [766]	Not general population
Penninx, 2017 [767]	Not general population
Petruzzelli, 2018 [768]	Not general population
Pickens, 1991 [769]	Not general population
Poon, 2017 [770]	Not general population

Author, year	Reason for exclusion
Post, 2015 [771]	Not general population
Post, 2016 [772]	Not general population
Poudel, 2017 [773]	Not general population
Prata, 2013 [774]	Not general population
Preisig, 2016 [775]	Not general population
Pulver, 1990 [776]	Not general population
Qian, 2016 [777]	Not general population
Queirazza, 2014 [778]	Not general population
Ramirez, 2015 [779]	Not general population
Rasgon, 2016 [780]	Not general population
Ridley, 1990 [781]	Not general population
Ritsner, 2010 [782]	Not general population
Ritter, 2014 [783]	Not general population
Robison, 2009 [784]	Not general population
Rognli, 2014 [785]	Not general population
Rubio-Abadal, 2015 [786]	Not general population
Saewyc, 1998 [787]	Not general population
Sartor, 2016 [788]	Not general population
Sathyan, 2014 [789]	Not general population
Schandrin, 2016 [790]	Not general population
Schimmelmann, 2007 [791]	Not general population
Segarra, 2012 [792]	Not general population
Seo, 2011 [793]	Not general population
Serretti, 2013 [794]	Not general population
Sharp, 1994 [795]	Not general population
Shaw, 2012 [796]	Not general population
Shi, 2015 [797]	Not general population
Shim, 2015 [798]	Not general population
Shimshoni, 2011 [799]	Not general population
Sibisi, 1990 [800]	Not general population
Silveira, 2011 [801]	Not general population
Skodol, 2005 [802]	Not general population
Sorensen, 2016 [803]	Not general population
Southwick, 2014 [804]	Not general population
Stassen, 1987 [805]	Not general population
Statham, 2014 [806]	Not general population
Stefanis, 2013 [807]	Not general population
Stegenga, 2012 [808]	Not general population
Steinberg, 1999 [809]	Not general population
Subramaniam, 2012 [810]	Not general population
Suchanek, 2012 [811]	Not general population
Sugranyes, 2009 [812]	Not general population
Sylvia, 2015 [813]	Not general population
Szerman, 2013 [814]	Not general population
Tabares-Seisdedos, 2016 [815]	Not general population
Tamas, 2006 [816]	Not general population
Tanskanen, 2009 [817]	Not general population
Timberlake, 2007 [818]	Not general population
Todd, 2008 [819]	Not general population
Tondo, 2010 [820]	Not general population
Tondo, 2014 [821]	Not general population
Torres, 2016 [822]	Not general population
Trim, 2009 [823]	Not general population
Truong, 2013 [824]	Not general population
van der Wee, 2011 [825]	Not general population
van der Wee, 2013 [826]	Not general population
Vandeleur, 2015 [827]	Not general population
Venisse, 2016 [828]	Not general population
Verhaak, 2015 [829]	Not general population
Viviani, 2003 [830]	Not general population
Vyas, 2007 [831]	Not general population
Wang, 2012 [832]	Not general population
Welham, 2003 [833]	Not general population
Wessely, 1998 [834]	Not general population

Author, year	Reason for exclusion
Wichow Icz, 2016 [835]	Not general population
Wilhelmsen, 2011 [836]	Not general population
Wilson, 2015 [837]	Not general population
Wolfl, 2009 [838]	Not general population
Woo, 2014 [839]	Not general population
Woodside, 1992 [840]	Not general population
Wu, 2008 [841]	Not general population
Yao, 2010 [842]	Not general population
Zaninotto, 2014 [843]	Not general population
Zhang, 2013 [844]	Not general population
Zhang, 2014 [845]	Not general population
Zhong, 2017 [846]	Not general population
Zhu, 2012 [847]	Not general population
Gilman, 2002 [848]	Overlapping population without additional estimates
Hafner, 1998 [849]	Overlapping population without additional estimates
Tian, 2017 [850]	Overlapping population without additional estimates
Wittchen, 1992 [851]	Overlapping population without additional estimates
Alegria, 2007 [852]	Prevalence
Andrade, 2003 [853]	Prevalence
Atladottir, 2014 [854]	Prevalence
Auerbach, 2017 [855]	Prevalence
Beesdo, 2007 [856]	Prevalence
Bland, 1988 [857]	Prevalence
Borges, 2017 [858]	Prevalence
Bourdon, 1988 [859]	Prevalence
Brennan, 2000 [860]	Prevalence
Carraro, 2015 [861]	Prevalence
Caye, 2016 [862]	Prevalence
Colman, 2008 [863]	Prevalence
Colman, 2009 [864]	Prevalence
de Graaf, 2016 [865]	Prevalence
Dickerson, 2012 [866]	Prevalence
Eaton, 1989 [867]	Prevalence
Gerstenberg, 2015 [868]	Prevalence
Grant, 1993 [869]	Prevalence
Grant, 1998 [870]	Prevalence
Grant, 1998 [871]	Prevalence
Harford, 2010 [872]	Prevalence
Harvey, 2009 [873]	Prevalence
Harvey, 2018 [874]	Prevalence
Hatch, 2009 [875]	Prevalence
Hatch, 2010 [876]	Prevalence
Heimberg, 2000 [877]	Prevalence
Helzer, 1990 [878]	Prevalence
Hennig, 2017 [879]	Prevalence
Isohanni, 2009 [880]	Prevalence
Kalaydjian, 2009 [881]	Prevalence
Karno, 2009 [882]	Prevalence
Kessler, 1998 [883]	Prevalence
Koenen, 2017 [884]	Prevalence
Kraus, 2000 [885]	Prevalence
Lai-Ming Hui C, 2015 [886]	Prevalence
Lee, 2009 [887]	Prevalence
Lev-Ran, 2013 [888]	Prevalence
Louie, 2018 [889]	Prevalence
Lukat, 2017 [890]	Prevalence
Marshall, 1990 [891]	Prevalence
Moffitt, 2005 [892]	Prevalence
Nagl, 2016 [893]	Prevalence
Nierenberg, 2010 [894]	Prevalence
Nock, 2007 [895]	Prevalence
Ouk Park, 2015 [896]	Prevalence
Paksarian, 2018 [897]	Prevalence
Patton, 2014 [898]	Prevalence

Author, year	Reason for exclusion
Perkonigg, 2000 [899]	Prevalence
Perkonigg, 2006 [900]	Prevalence
Ravens-Sieberer, 2015 [901]	Prevalence
Rees, 2014 [902]	Prevalence
Ruggeri, 2013 [903]	Prevalence
Sandanger, 1999 [904]	Prevalence
Shetye, 2007 [905]	Prevalence
Simon, 1992 [906]	Prevalence
Stice, 2010 [907]	Prevalence
Sung, 2004 [908]	Prevalence
Van Milligen, 2011 [909]	Prevalence
Vilalta-Franch, 2012 [910]	Prevalence
Wang, 2003 [911]	Prevalence
Weissman, 1984 [912]	Prevalence
White, 2015 [913]	Prevalence
Wittchen, 2008 [914]	Prevalence
Alvarado, 2012 [915]	Review
Angermeyer, 1988 [916]	Review
Bucholz, 1999 [917]	Review
Burcusa, 2007 [918]	Review
Burns, 2011 [919]	Review
Clarizio, 1989 [920]	Review
De Lijster, 2017 [921]	Review
Flor-Henry, 1985 [922]	Review
Gold, 1984 [923]	Review
Hardy, 2018 [924]	Review
Kessler, 2013 [925]	Review
Loeber, 1991 [926]	Review
Spinhoven, 2013[927]	Review
Winter, 2011 [928]	Review
Zoccolillo, 1999 [929]	Review
Welch, 2010 [930]	Unable to find the article

e-table 5. Comparison of age at onset across mental disorders

	Median	Phobias/Separation anxiety	ASD	ADHD	Social anxiety	Anorexia nervosa	Bulimia nervosa	OCD	Binge eating	Cannabis use disorder	PD	Schizophrenia	Panic disorder	Alcohol use disorder	PTSD	Depressive disorder	GAD	Bipolar disorder	ATPD
**Phobias/Separation anxiety**	**8**		0.260	0.073	<0.001	<0.001	<0.001	<0.001	<0.001	<0.001	0.192	<0.001	<0.001	<0.001	<0.001	<0.001	<0.001	<0.001	<0.001
**ASD**	**9**	0.263		0.341	0.327	0.309	0.314	0.327	0.327	0.267	0.490	0.308	0.243	0.209	0.039	0.091	<0.001	0.132	<0.001
**ADHD**	**12**	0.075	0.340		0.403	0.157	0.122	0.122	0.122	0.122	0.382	0.122	0.122	0.122	0.122	0.122	0.122	0.123	0.122
**Social anxiety**	**13**	<0.001	0.327	0.408		0.080	<0.001	<0.001	<0.001	<0.001	0.286	<0.001	<0.001	<0.001	<0.001	<0.001	<0.001	<0.001	<0.001
**Anorexia nervosa**	**17**	<0.001	0.307	0.155	0.080		0.269	0.318	0.210	0.150	0.441	0.121	0.114	0.139	0.086	0.100	0.082	0.110	0.082
**Bulimia nervosa**	**18**	<0.001	0.313	0.122	<0.001	0.268		0.520	0.541	0.176	0.466	0.091	0.088	0.196	0.079	0.098	0.034	0.085	0.032
**OCD**	**19**	<0.001	0.327	0.122	<0.001	0.320	0.515		0.167	0.040	0.374	0.001	0.001	0.048	<0.001	0.005	<0.001	0.015	<0.001
**Binge eating**	**20**	<0.001	0.327	0.122	<0.001	0.211	0.538	0.164		0.040	0.412	<0.001	<0.001	0.102	<0.001	<0.001	<0.001	0.040	<0.001
**Cannabis use disorder**	**22**	<0.001	0.269	0.122	<0.001	0.150	0.177	0.038	0.041		0.811	0.427	0.387	0.771	0.299	0.408	0.166	0.357	0.125
**Personality disorder**	**25**	0.190	0.493	0.381	0.286	0.437	0.465	0.373	0.412	0.810		0.703	0.440	0.880	0.233	0.459	0.197	0.468	0.193
**Schizophrenia**	**25**	<0.001	0.308	0.123	<0.001	0.121	0.091	0.001	<0.001	0.429	0.704		0.403	0.831	0.031	0.381	<0.001	0.479	<0.001
**Panic disorder**	**26**	<0.001	0.245	0.122	<0.001	0.114	0.088	0.001	<0.001	0.388	0.440	0.402		0.745	0.250	0.572	0.034	0.642	0.030
**Alcohol use disorder**	**27**	<0.001	0.209	0.122	<0.001	0.139	0.192	0.044	0.102	0.768	0.876	0.830	0.747		0.540	0.659	0.230	0.549	0.174
**PTSD**	**30**	<0.001	0.043	0.122	<0.001	0.086	0.080	<0.001	<0.001	0.298	0.231	0.031	0.249	0.545		0.811	0.157	0.933	0.154
**Depressive disorder**	**30**	<0.001	0.098	0.122	<0.001	0.099	0.100	0.005	<0.001	0.413	0.454	0.381	0.574	0.656	0.818		0.035	0.742	<0.001
**GAD**	**32**	<0.001	<0.001	0.122	<0.001	0.082	0.033	<0.001	<0.001	0.167	0.196	<0.001	0.034	0.228	0.156	0.039		0.611	0.591
**Bipolar disorder**	**33**	<0.001	0.137	0.122	<0.001	0.111	0.085	0.018	0.039	0.355	0.467	0.477	0.640	0.551	0.933	0.747	0.611		0.485
**ATPD**	**35**	<0.001	<0.001	0.122	<0.001	0.082	0.033	<0.001	<0.001	0.124	0.194	<0.001	0.031	0.172	0.152	<0.001	0.592	0.484	

Legend. ADHD, attention deficit/hyperactivity disorder; ASD, autism spectrum disorder; ATPD, acute and transient psychotic disorder; Binge eating, binge eating disorder; Bipolar disorder, bipolar or related disorders; GAD, generalised anxiety disorder; OCD, obsessive-compulsive disorder; Phobias/Separation anxiety, specific phobias / separation anxiety disorder; PTSD, post-traumatic stress disorder; Social anxiety, social anxiety disorder. Cells contain p value of pairwise comparisons.

Supplementary references

1. Nelder JA, Mead R. A Simplex Method for Function Minimization. Comput J. 1965;7:308–313.
2. R Foundation for Statistical Computing, Vienna AU https://www. R org/. R Core Team (2019). R: A language and environment for statistical computing.
3. Moher D, Liberati A, Tetzlaff J, Altman DG. Preferred reporting items for systematic reviews and meta-analyses: the PRISMA statement. J Clin Epidemiol. 2009;62:1006–1012.
4. Stroup DF, Berlin JA, Morton SC, Olkin I, Williamson GD, Rennie D, et al. Meta-analysis of observational studies in epidemiology: A proposal for reporting. J Am Med Assoc. 2000;283:2008–2012.
5. Abajobir AA, Najman JM, Williams G, Strathearn L, Clavarino A, Kisely S. Substantiated childhood maltreatment and young adulthood cannabis use disorders: A pre-birth cohort study. Psychiatry Res. 2017;256:21–31.
6. Al-Hamzawi A, Al-Diwan JK, Al-Hasnawi SM, Taib NI, Chatterji S, Hwang I, et al. The prevalence and correlates of intermittent explosive disorder in Iraq. Acta Psychiatr Scand. 2012;126:219–228.
7. Alem A, Kebede D, Shibre T, Fekadu A, Kullgren G, Medhin G, et al. Clinical Course and Outcome of Schizophrenia in a Predominantly Treatment-Naive Cohort in Rural Ethiopia. Schizophr Bull. 2008;35:646–654.
8. Al-Hamzawi AO, Bruffaerts R, Bromet EJ, AlKhafaji AM, Kessler RC. The Epidemiology of Major Depressive Episode in the Iraqi General Population. PLoS One. 2015;10:e0131937.
9. Angst J, Cui L, Swendsen J, Rothen S, Cravchik A, Kessler RC, et al. Major depressive disorder with subthreshold bipolarity in the national comorbidity survey replication. Am J Psychiatry. 2010. 2010. https://doi.org/10.1176/appi.ajp.2010.09071011.
10. Angst J, Gamma A, Baldwin DS, Ajdacic-Gross V, Rössler W. The generalized anxiety spectrum: prevalence, onset, course and outcome. Eur Arch Psychiatry Clin Neurosci. 2009;259:37—45.
11. Angst JI, Merikangas K. Recurrent brief depression : a new subtype of affective disorder. J Affect Disord 19(2)87-98, 1990 Jun. 1990;19:87–98.
12. Bantjes J, Lochner C, Saal W, Roos J, Taljaard L, Page D, et al. Prevalence and sociodemographic correlates of common mental disorders among first-year university students in post-apartheid South Africa: implications for a public mental health approach to student wellness. BMC Public Health. 2019;19:922.
13. Becker ES, Rinck M, Türke V, Kause P, Goodwin R, Neumer S, et al. Epidemiology of specific phobia subtypes: findings from the Dresden Mental Health Study. Eur Psychiatry. 2007;22:69–74.
14. Bienvenu OJ, Eaton WW. The epidemiology of blood-injection-injury phobia. Psychol Med. 1998;28:1129–1136.
15. Birrell L, Newton NC, Teesson M, Slade T. Early onset mood disorders and first alcohol use in the general population. J Affect Disord. 2016;200:243–249.
16. Blanco C, Vesga-López O, Stewart JW, Liu SM, Grant BF, Hasin DS. Epidemiology of major depression with atypical features: Results from the National Epidemiologic Survey on Alcohol and Related Conditions (NESARC). J Clin Psychiatry. 2012. 2012. https://doi.org/10.4088/JCP.10m06227.
17. Bland RC, Newman SC, Orn H. Age of Onset of Psychiatric Disorders. Acta Psychiatr Scand. 1988;77:43–49.
18. Bogren M, Brådvik L, Holmstrand C, Nöbbelin L, Mattisson C. Gender differences in subtypes of depression by first incidence and age of onset: a follow-up of the Lundby population. Eur Arch

Psychiatry Clin Neurosci. 2018;268:179—189.

19. Bonnewyn A, Bruffaerts R, Vilagut G, Almansa J, Demyttenaere K. Lifetime risk and age-of-onset of mental disorders in the Belgian general population. Soc Psychiatry Psychiatr Epidemiol. 2007;42:522–529.

20. Borges G, Wang PS, Medina-Mora ME, Lara C, Chiu WT. Delay of first treatment of mental and substance use disorders in Mexico. Am J Public Health. 2007. 2007. https://doi.org/10.2105/AJPH.2006.090985.

21. Bromet E, Andrade LH, Hwang I, Sampson NA, Alonso J, de Girolamo G, et al. Cross-national epidemiology of DSM-IV major depressive episode. BMC Med. 2011;9.

22. Bromet EJ, Gluzman SF, Paniotto VI, Webb CPM, Tintle NL, Zakhozha V, et al. Epidemiology of psychiatric and alcohol disorders in Ukraine: Findings from the Ukraine World Mental Health Survey. Soc Psychiatry Psychiatr Epidemiol. 2005;40:681–690.

23. Buckner JD, Heimberg RG, Schneier FR, Liu SM, Wang S, Blanco C. The relationship between cannabis use disorders and social anxiety disorder in the National Epidemiological Study of Alcohol and Related Conditions (NESARC). Drug Alcohol Depend. 2012;124:128–134.

24. Burke KC, Burke Jr JD, Regier DA, Rae DS. Age at Onset of Selected Mental Disorders in Five Community Populations. Arch Gen Psychiatry. 1990;47:511–518.

25. Harker Burnhams N, Bharat C, Williams DR, Stein DJ, Myers B. Transitions between lifetime alcohol use, regular use and remission: Results from the 2004 South African Stress and Health Survey. S Afr Med J. 2018;109:40–46.

26. Burns JK, Jhazbhay K, Emsley RA. Causal attributions, pathway to care and clinical features of first-episode psychosis: A South African perspective. Int J Soc Psychiatry. 2011;57:538–545.

27. Burstein M, He JP, Kattan G, Albano AM, Avenevoli S, Merikangas KR. Social phobia and subtypes in the National Comorbidity Survey-Adolescent Supplement: Prevalence, correlates, and comorbidity. J Am Acad Child Adolesc Psychiatry. 2011. 2011. https://doi.org/10.1016/j.jaac.2011.06.005.

28. Burstein M, Georgiades K, He JP, Schmitz A, Feig E, Khazanov GK, et al. Specific phobia among U.S. adolescents: Phenomenology and typology. Depress Anxiety. 2012. 2012. https://doi.org/10.1002/da.22008.

29. Caraveo-Anduaga JJ, Colmenares Bermúdez E. The epidemiology of obsessive-compulsive disorder in Mexico City. Salud Ment. 2004. 2004.

30. Castagnini A, Foldager L. Variations in incidence and age of onset of acute and transient psychotic disorders. Soc Psychiatry Psychiatr Epidemiol. 2013;48:1917–1922.

31. Chang WC, Tang JYM, Hui CLM, Chiu CPY, Lam MML, Wong GHY, et al. Gender differences in patients presenting with first-episode psychosis in Hong Kong: A three-year follow up study. Aust N Z J Psychiatry. 2011;45:199–205.

32. Chapman C, Mills K, Slade T, McFarlane AC, Bryant RA, Creamer M, et al. Remission from post-traumatic stress disorder in the general population. Psychol Med. 2012;42:1695–1703.

33. Cheng AT, Chen WJ. Alcoholism among four aboriginal groups in Taiwan: high prevalences and their implications. Alcohol Clin Exp Res. 1995;19:81—91.

34. Cho MJ, Chang SM, Hahm B-J, Chung I-W, Bae A, Lee YM, et al. Lifetime risk and age of onset distributions of psychiatric disorders: analysis of national sample survey in South Korea. Soc Psychiatry Psychiatr Epidemiol. 2012;47:671–681.

35. Chong SA, Vaingankar J, Abdin E, Subramaniam M. The prevalence and impact of major depressive disorder among Chinese, Malays and Indians in an Asian multi-racial population. J Affect Disord. 2012;138:128–136.

36. Christie KA, Burke JD, Regier DA, Rae DS, Boyd JH, Locke BZ. Epidemiologic evidence for early onset of mental disorders and higher risk of drug abuse in young adults. Am J Psychiatry. 1988;145:971—975.

37. Vommaro H, Cía AH, Loera G, Kessler RC, Medina-Mora ME, Stagnaro JC, et al. Lifetime prevalence and age-of-onset of mental disorders in adults from the Argentinean Study of Mental Health Epidemiology. Soc Psychiatry Psychiatr Epidemiol. 2018;53:341–350.

38. Cilliçilli AS, Telcioglu M, Aşkin R, Kaya N, Bodur S, Kucur R. Twelve-month prevalence of obsessive-compulsive disorder in Konya, Turkey. Compr Psychiatry. 2004;45:367—374.

39. Cooper B, Eagles JM. 'Schizophrenia in Croatia: Inter-regional differences in prevalence and comment on constant incidence': Comment. Br J Psychiatry. 1994;164:97–100.

40. Dakwar E, Levin FR, Olfson M, Wang S, Kerridge B, Blanco C. First treatment contact for ADHD: predictors of and gender differences in treatment seeking. Psychiatr Serv. 2014;65:1465—1473.

41. Dalsgaard S, Thorsteinsson E, Trabjerg BB, Schullehner J, Plana-Ripoll O, Brikell I, et al. Incidence Rates and Cumulative Incidences of the Full Spectrum of Diagnosed Mental Disorders in Childhood and Adolescence. JAMA Psychiatry. 2019. November 2019. https://doi.org/10.1001/jamapsychiatry.2019.3523.

42. Browne MO, Moskalewicz J, Posada-Villa J, Levinson D, Haro JM, Gureje O, et al. Cross-national epidemiology of panic disorder and panic attacks in the world mental health surveys. Depress Anxiety. 2016;33:1155–1177.

43. de Vries YA, Ten Have M, de Graaf R, van Dorsselaer S, de Ruiter NMP, de Jonge P. The relationship between mental disorders and actual and desired subjective social status. Epidemiol Psychiatr Sci. 2019;29:e83.

44. Kessler RC, Bharat C, Peacock A, Lago L, Sampson N, Degenhardt L, et al. The impact of cohort substance use upon likelihood of transitioning through stages of alcohol and cannabis use and use disorder: Findings from the Australian National Survey on Mental Health and Wellbeing. Drug Alcohol Rev. 2018;61:546–556.

45. Deutsch AR, Slutske WS, Lynskey MT, Bucholz KK, Madden PAF, Heath AC, et al. From alcohol initiation to tolerance to problems: Discordant twin modeling of a developmental process. Dev Psychopathol. 2017;29:845–861.

46. Dussault F, Dufour M, Brunelle N, Tremblay J, Rousseau M, Leclerc D, et al. Consistency of Adolescents' Self-Report of Gambling Age of Onset: A Longitudinal Study. J Gambl Stud. 2019;35:533–544.

47. Esan O, Makanjuola V, Oladeji B, Gureje O. Determinants of transition across the spectrum of alcohol use and misuse in Nigeria. Alcohol. 2013;47:249–255.

48. Ezpeleta L, Navarro JB, de la Osa N, Penelo E, Domènech JM. First incidence, age of onset outcomes and risk factors of onset of DSM-5 oppositional defiant disorder: a cohort study of Spanish children from ages 3 to 9. BMJ Open. 2019;9:e022493.

49. Falk DE, Yi H, Hilton ME. NIH Public Access. Alcohol. 2009;94:234–245.

50. Faravelli C, Zucchi T, Viviani B, Salmoria R, Perone A, Paionni A, et al. Epidemiology of social phobia: A clinical approach. Eur Psychiatry. 2000;15:17–24.

51. Farmer RF, Kosty DB, Seeley JR, Duncan SC, Lynskey MT, Rohde P, et al. Natural course of cannabis use disorders. Psychol Med. 2015;45:63–72.

52. Fava M, Hwang I, Rush AJ, Sampson N, Walters EE, Kessler RC. The importance of irritability as a symptom of major depressive disorder: Results from the national comorbidity survey replication. Mol Psychiatry. 2010. 2010. https://doi.org/10.1038/mp.2009.20.

53. Fernandez-Pujals AM, Adams MJ, Thomson P, McKechanie AG, Blackwood DHR, Smith BH, et al. Epidemiology and heritability of major depressive disorder, stratified by age of onset, sex, and illness course in generation Scotland: Scottish family health study (GS: SFHS). PLoS One. 2015;10:1–18.

54. Fogarty F, Russell JM, Newman SC, Bland RC. Mania. Acta Psychiatr Scand. 1994;89:16–23.

55. Gabilondo A, Rojas-Farreras S, Vilagut G, Haro JM, Fernández A, Pinto-Meza A, et al. Epidemiology of major depressive episode in a southern European country: Results from the ESEMeD-Spain project. J Affect Disord. 2010;120:76–85.

56. Garfinkel PE, Lin E, Goering P, Spegg C, Goldbloom DS, Kennedy S, et al. Purging and nonpurging forms of bulimia nervosa in a community sample. Int J Eat Disord. 1996;20:231–238.

57. Gilder DA, Lau P, Corey L, Ehlers CL. Factors Associated With Remission From Alcohol Dependence in an American Indian Community Group. Am J Psychiatry. 2008;165:1172–1178.

58. Gilder DA, Stouffer GM, Lau P, Ehlers CL. Clinical characteristics of alcohol combined with other substance use disorders in an American Indian community sample. Drug Alcohol Depend. 2016;161:222–229.

59. GILMAN SE, KAWACHI I, FITZMAURICE GM, BUKA SL. Socio-economic status, family disruption and residential stability in childhood: relation to onset, recurrence and remission of major depression. Psychol Med. 2003;33:1341–1355.

60. Glantz MD, Medina-Mora ME, Petukhova M, Andrade LH, Anthony JC, De Girolamo G, et al. Alcohol abuse in developed and developing countries in the World Mental Health Surveys: Socially defined consequences or psychiatric disorder? Am J Addict. 2014;23:145–155.

61. Gonçalves DC, Byrne GJ. Sooner or later: Age at onset of generalized anxiety disorder in older adults. Depress Anxiety. 2012. 2012. https://doi.org/10.1002/da.20881.

62. González HM, Tarraf W, Whitfield KE, Vega WA. The epidemiology of major depression and ethnicity in the United States. J Psychiatr Res. 2010;44:1043–1051.

63. Griesler PC, Hu MC, Schaffran C, Kandel DB. Comorbid psychiatric disorders and nicotine dependence in adolescence. Addiction. 2011. 2011. https://doi.org/10.1111/j.1360-0443.2011.03403.x.

64. Gureje O, Uwakwe R, Oladeji B, Makanjuola VO, Esan O. Depression in adult Nigerians: Results from the Nigerian Survey of Mental Health and Well-being. J Affect Disord. 2010;120:158–164.

65. Gureje O, Uwakwe R, Udofia O, Wakil M, Adeyemi O, Enyidah N. Common psychiatric disorders over a lifetime: age of onset, risk and treatment contact in the Nigerian survey of mental health and wellbeing. Afr J Med Med Sci. 2008;37:207–217.

66. Häfner H, Riecher A, Maurer K, Löffler W, Munk-Jørgensen P, Strömgren E. How does gender influence age at first hospitalization for schizophrenia? Psychol Med. 1989;19:903–918.

67. Hafner H, Maurer K, Loffler W, Fatkenheuer B, An der Heiden W, Riecher- Rossler A, et al. The epidemiology of early schizophrenia. Influence of age and gender on onset and early course. Br. J. Psychiatry, vol. 164, 1994. p. 29–38.

68. Hahm BJ, Cho MJ. Prevalence of alcohol use disorder in a South Korean community. Changes in the pattern of prevalence over the past 15 years. Soc Psychiatry Psychiatr Epidemiol. 2005;40:114–119.

69. Hardeveld F, Spijker J, De Graaf R, Nolen WA, Beekman ATF. Recurrence of major depressive disorder and its predictors in the general population: Results from the Netherlands Mental Health Survey and Incidence Study (NEMESIS). Psychol Med. 2013. 2013. https://doi.org/10.1017/S0033291712002395.

70. Hines LA, Morley KI, Strang J, Agrawal A, Nelson EC, Statham D, et al. Onset of opportunity to use cannabis and progression from opportunity to dependence: Are influences consistent across

transitions? Drug Alcohol Depend. 2016;160:57–64.

71. Hoertel N, Blanco C, Oquendo MA, Wall MM, Olfson M, Falissard B, et al. A comprehensive model of predictors of persistence and recurrence in adults with major depression: Results from a national 3-year prospective study. J Psychiatr Res. 2017;95:19––27.

72. Klein Hofmeijer-Sevink M, Batelaan NM, Van Megen HJGM, Penninx BW, Cath DC, Van Den Hout MA, et al. Clinical relevance of comorbidity in anxiety disorders: A report from the Netherlands Study of Depression and Anxiety (NESDA). J Affect Disord. 2012;137:106–112.

73. Hsu C-W, Lee S-Y, Wang L-J. Gender differences in the prevalence, comorbidities and antipsychotic prescription of early-onset schizophrenia: a nationwide population-based study in Taiwan. Eur Child Adolesc Psychiatry. 2019;28:759–767.

74. Hudson JI, Hiripi E, Pope HG, Kessler RC. The Prevalence and Correlates of Eating Disorders in the National Comorbidity Survey Replication. Biol Psychiatry. 2007;61:348–358.

75. Isohanni I, Jones PB, Järvelin MR, Nieminen P, Rantakallio P, Jokelainen J, et al. Educational consequences of mental disorders treated in hospital. A 31-year follow-up of the Northern Finland 1966 Birth Cohort. Psychol Med. 2001;31:339––349.

76. Joinson C, Kounali D, Lewis G. Family socioeconomic position in early life and onset of depressive symptoms and depression: a prospective cohort study. Soc Psychiatry Psychiatr Epidemiol. 2017;52:95–103.

77. Jones P, Murray R, Jones P, Rodgers B, Marmot M. Child developmental risk factors for adult schizophrenia in the British 1946 birth cohort. Lancet. 1994;344:1398–1402.

78. Karam EG, Friedman MJ, Hill ED, Kessler RC, McLaughlin KA, Petukhova M, et al. Cumulative traumas and risk thresholds: 12-month ptsd in the world mental health (WMH) surveys. Depress Anxiety. 2014;31:130–142.

79. Karam EG, Mneimneh ZN, Dimassi H, Fayyad JA, Karam AN, Nasser SC, et al. Lifetime prevalence of mental disorders in Lebanon: First onset, treatment, and exposure to war. PLoS Med. 2008;5:0579–0586.

80. Kasch KL, Klein DN. The relationship between age at onset and comorbidity in psychiatric disorders. J Nerv Ment Dis. 1996;184:703–707.

81. Merikangas KR, Jin R, He JP, Kessler RC, Lee S, Sampson NA, et al. Prevalence and correlates of bipolar spectrum disorder in the World Mental Health Survey Initiative. Arch Gen Psychiatry. 2011;68:241–251.

82. Kebede D, Alem A, Shibre T, Negash A, Fekadu A, Fekadu D, et al. Onset and clinical course of schizophrenia in Butajira-Ethiopia--a community-based study. Soc Psychiatry Psychiatr Epidemiol. 2003;38:625–631.

83. Keenan K, Wroblewski K, Hipwell A, Loeber R, Stouthamer-Loeber M. Age of onset, symptom threshold, and expansion of the nosology of conduct disorder for girls. J Abnorm Psychol. 2010. 2010. https://doi.org/10.1037/a0019346.

84. Kendell R, Kemp I. Winter-Born v Summer-Born Schizophrenics. Br J Psychiatry 151499-505, 1987 Oct. 1987:499–505.

85. Kessler RC, Avenevoli S, McLaughlin KA, Green JG, Lakoma MD, Petukhova M, et al. Lifetime co-morbidity of DSM-IV disorders in the US National Comorbidity Survey Replication Adolescent Supplement (NCS-A). Psychol Med. 2012. 2012. https://doi.org/10.1017/S0033291712000025.

86. Kessler RC, Berglund P, Demler O, Jin R, Merikangas KR, Walters EE. Lifetime prevalence and age-of-onset distributions of DSM-IV disorders in the national comorbidity survey replication. Arch Gen Psychiatry. 2005.

87. Kessler RC, Crum RM, Warner LA, Nelson CB, Schulenberg J, Anthony JC. Lifetime Co-occurrence of DSM-III-R Alcohol Abuse and Dependence With Other Psychiatric Disorders in the National Comorbidity Survey. Arch Gen Psychiatry. 1997;54:313–321.

88. Kessler RC, Berglund PA, Chiu WT, Deitz AC, Hudson JI, Shahly V, et al. The prevalence and correlates of binge eating disorder in the World Health Organization World Mental Health Surveys. Biol Psychiatry. 2013;73:904–914.

89. Kessler RC, Angermeyer M, Anthony JC, DE Graaf R, Demyttenaere K, Gasquet I, et al. Lifetime prevalence and age-of-onset distributions of mental disorders in the World Health Organization's World Mental Health Survey Initiative. World Psychiatry. 2007. 2007.

90. Kessler RC, Shahly V, Hudson JI, Supina D, Berglund PA, Chiu WT, et al. A comparative analysis of role attainment and impairment in binge-eating disorder and bulimia nervosa: results from the WHO World Mental Health Surveys. Epidemiol Psychiatr Sci. 2014;23:27–41.

91. Kessler RC, Chiu WT, Jin R, Ruscio AM, Shear K, Walters EE. The epidemiology of panic attacks, panic disorder, and agoraphobia in the National Comorbidity Survey Replication. Arch Gen Psychiatry. 2006;63:415—424.

92. Kim SW, Stewart R, Kim JM, Shin IS, Yoon JS, Jung SW, et al. Relationship between a history of a suicide attempt and treatment outcomes in patients with depression. J Clin Psychopharmacol. 2011;31:449–456.

93. Kim-Cohen J, Caspi A, Moffitt TE, Harrington HL, Milne BJ, Poulton R. Prior juvenile diagnoses in adults with mental disorder: Developmental follow-back of a prospective-longitudinal cohort. Arch Gen Psychiatry. 2003;60:709–717.

94. Kirkbride JB, Fearon P, Morgan C, Dazzan P, Morgan K, Tarrant J, et al. Heterogeneity in incidence rates of schizophrenia and other psychotic syndromes: findings from the 3-center AeSOP study. Arch Gen Psychiatry. 2006;63:250–258.

95. Knappe S, Beesdo-Baum K, Fehm L, Stein MB, Lieb R, Wittchen HU. Social fear and social phobia types among community youth: Differential clinical features and vulnerability factors. J Psychiatr Res. 2011. 2011. https://doi.org/10.1016/j.jpsychires.2010.05.002.

96. Köhler S, van der Werf M, Hart B, Morrison G, McCreadie R, Kirkpatrick B, et al. Evidence that better outcome of psychosis in women is reversed with increasing age of onset: a population-based 5-year follow-up study. Schizophr Res. 2009;113:226–232.

97. Korten NCM, Comijs HC, Lamers F, Penninx BWJH. Early and late onset depression in young and middle aged adults: Differential symptomatology, characteristics and risk factors? J Affect Disord. 2012;138:259–267.

98. Lahey BB, Loeber R, Quay HC, Applegate B, Shaffer D, Waldman I, et al. Validity of DSM-IV subtypes of conduct disorder based on age of onset. J Am Acad Child Adolesc Psychiatry. 1998;37:435–442.

99. Lahti M, Eriksson JG, Heinonen K, Kajantie E, Lahti J, Wahlbeck K, et al. Maternal grand multiparity and the risk of severe mental disorders in adult offspring. PLoS One. 2014;9:1–19.

100. Le Strat Y, Grant BF, Ramoz N, Gorwood P. A new definition of early age at onset in alcohol dependence. Drug Alcohol Depend. 2010;108:43–48.

101. Lee S, Tsang A, Kessler RC, Jin R, Sampson N, Andrade L, et al. Rapid-cycling bipolar disorder: Cross-national community study. Br J Psychiatry. 2010. 2010. https://doi.org/10.1192/bjp.bp.109.067843.

102. Lee S, Tsang A, Zhang MY, Huang YQ, He YL, Liu ZR, et al. Lifetime prevalence and inter-cohort variation in DSM-IV disorders in metropolitan China. Psychol Med. 2007;37:61–71.

103. Lee S, Tsang A, Huang Y-Q, He Y-L, Liu ZR, Zhang M-Y, et al. The epidemiology of depression in metropolitan China. Psychol Med. 2009;39:735–747.

104. Lee S, Tsang A, Ruscio AM, Haro JM, Stein DJ, Alonso J, et al. Implications of modifying the duration requirement of generalized anxiety disorder in developed and developing countries. Psychol Med. 2009. 2009. https://doi.org/10.1017/S0033291708004807.

105. Lépine JP, Lellouch J. Classification and epidemiology of social phobia. Eur Arch Psychiatry Clin Neurosci. 1995;244:290—296.

106. Lepine JP, Lellouch J. Diagnosis and epidemiology of agoraphobia and social phobia. Clin Neuropharmacol. 1995. 1995. https://doi.org/10.1097/00002826-199518002-00004.

107. Levine SZ, Rabinowitz J. A population-based examination of the role of years of education, age of onset, and sex on the course of schizophrenia. Psychiatry Res. 2009;168:11–17.

108. Levinson D, Zilber N, Lerner Y, Grinshpoon A, Levav I. Prevalence of mood and anxiety disorders in the community: Results from the Israel national health survey. Isr J Psychiatry Relat Sci. 2007;44:94–103.

109. Lewis CE, Bucholz KK, Spitznagel E, Shayka JJ. Effects of gender and comorbidity on problem drinking in a community sample. Alcohol Clin Exp Res. 1996;20:466–476.

110. Luoma S, Hakko H, Ollinen T, Järvelin MR, Lindeman S. Association between age at onset and clinical features of schizophrenia: The Northern Finland 1966 birth cohort study. Eur Psychiatry. 2008. 2008. https://doi.org/10.1016/j.eurpsy.2008.03.005.

111. Manetti A, Hoertel N, Le Strat Y, Schuster JP, Lemogne C, Limosin F. Comorbidity of late-life depression in the united states: A population-based study. Am J Geriatr Psychiatry. 2014;22:1292–1306.

112. Mattisson C, Bogren M, Öjehagen A, Nordström G, Horstmann V. Mortality in alcohol use disorder in the Lundby Community Cohort--a 50 year follow-up. Drug Alcohol Depend. 2011;118:141–147.

113. McEvoy PM, Grove R, Slade T. Epidemiology of anxiety disorders in the Australian general population: Findings of the 2007 Australian National Survey of Mental Health and Wellbeing. Aust N Z J Psychiatry. 2011;45:957–967.

114. McLaughlin KA, Green JG, Hwang I, Sampson NA, Zaslavsky AM, Kessler RC. Intermittent explosive disorder in the national comorbidity survey replication adolescent supplement. Arch Gen Psychiatry. 2012. 2012. https://doi.org/10.1001/archgenpsychiatry.2012.592.

115. Medina-Mora ME, Borges G, Benjet C, Lara C, Berglund P. Psychiatric disorders in Mexico: Lifetime prevalence in a nationally representative sample. Br J Psychiatry. 2007;190:521–528.

116. Merikangas KR, He J, Burstein M, Swanson SA, Avenevoli S, Cui L, et al. Lifetime Prevalence of Mental Disorders in US Adolescents: Results from the National Comorbidity Study-Adolescent. J Am Acad Child Adolesc Psychiatry. 2010;49:980–989.

117. Merikangas KR, Akiskal HS, Angst J, Greenberg PE, Hirschfeld RMA, Petukhova M, et al. Lifetime and 12-month prevalence of bipolar spectrum disorder in the National Comorbidity Survey replication. Arch Gen Psychiatry. 2007;64:543—552.

118. Mojtabai R. Bereavement-related depressive episodes: Characteristics, 3-year course, and implications for the DSM-5. Arch Gen Psychiatry. 2011. 2011. https://doi.org/10.1001/archgenpsychiatry.2011.95.

119. Mojtabai R. Impairment in major depression: Implications for diagnosis. Compr Psychiatry. 2001. 2001. https://doi.org/10.1053/comp.2001.23142.

120. Murphy JA, Byrne GJ. Prevalence and correlates of the proposed DSM-5 diagnosis of Chronic Depressive Disorder. J Affect Disord. 2012. 2012. https://doi.org/10.1016/j.jad.2012.01.033.

121. Navarro-Mateu F, Tormo MJ, Salmerón D, Vilagut G, Navarro C, Ruíz-Merino G, et al. Prevalence of mental disorders in the South-East of Spain, one of the European regions most affected by the economic crisis: The cross-sectional PEGASUS-Murcia project. PLoS One. 2015;10:1–22.

122. Negash A, Kullgren G, Shibre T, Kebede D, Alem A, Deyessa N. Prevalence and clinical characteristics of bipolar I disorder in Butajira, Ethiopia: A community-based study. J Affect Disord. 2005;87:193–201.

123. Nelson CB, Little RJ, Heath AC, Kessler RC. Patterns of DSM-III-R alcohol dependence symptom progression in a general population survey. Psychol Med. 1996;26:449–460.

124. Nesvåg R, Bramness JG, Handal M, Hartz I, Hjellvik V, Skurtveit S. The incidence, psychiatric co-morbidity and pharmacological treatment of severe mental disorders in children and adolescents. Eur Psychiatry. 2018;49:16–22.

125. Nordström T, Hurtig T, Moilanen I, Taanila A, Ebeling H. Disruptive behaviour disorder with and without attention deficit hyperactivity disorder is a risk of psychiatric hospitalization. Acta Paediatr Int J Paediatr. 2013;102:1100–1103.

126. Oakley Browne MA, Elisabeth Wells J, Scott KM, Mcgee MA. Lifetime Prevalence and Projected Lifetime Risk of DSM-IV Disorders in Te Rau Hinengaro: The New Zealand Mental Health Survey. Aust New Zeal J Psychiatry. 2006;40:865–874.

127. Ormel J, Raven D, Van Oort F, Hartman CA, Reijneveld SA, Veenstra R, et al. Mental health in Dutch adolescents: A TRAILS report on prevalence, severity, age of onset, continuity and co-morbidity of DSM disorders. Psychol Med. 2015;45:345–360.

128. Orvaschel H, Lewinson PM, Seeley JR. Continuity of Psychopathology in a Community Sample of Adolescents. J Am Acad Child Adolesc Psychiatry. 1995;34:1525–1535.

129. Mortensen PB, Pedersen CB, McGrath JJ, Bertelsen A, Agerbo E, Eaton WW, et al. A Comprehensive Nationwide Study of the Incidence Rate and Lifetime Risk for Treated Mental Disorders. JAMA Psychiatry. 2014;71:573.

130. Perälä J, Kuoppasalmi K, Pirkola S, Härkänen T, Saarni S, Tuulio-Henriksson A, et al. Alcohol-induced psychotic disorder and delirium in the general population. Br J Psychiatry. 2010. 2010. https://doi.org/10.1192/bjp.bp.109.070797.

131. Peralta V, de Jalón EG, Campos MS, Zandio M, Sanchez-Torres A, Cuesta MJ. The meaning of childhood attention-deficit hyperactivity symptoms in patients with a first-episode of schizophrenia-spectrum psychosis. Schizophr Res. 2011;126:28–35.

132. Peyre H, Hoertel N, Cortese S, Acquaviva E, De Maricourt P, Limosin F, et al. Attention-deficit/hyperactivity disorder symptom expression: A comparison of individual age at onset using item response theory. J Clin Psychiatry. 2014;75:386–392.

133. Polo AJ, Alegría M, Chen CN, Blanco C. The prevalence and comorbidity of social anxiety disorder among United States latinos: A retrospective analysis of data from 2 national surveys. J Clin Psychiatry. 2011. 2011. https://doi.org/10.4088/JCP.08m04436.

134. J. R, S. F. Differences in age of first hospitalization for schizophrenia among immigrants and nonimmigrants in a National Case Registry. Schizophr Bull. 2002;28:491–499.

135. Ramage-Morin PL. Panic disorder and coping. Health Rep. 2004;15 Suppl:31–43.

136. M.-S. R, M.-Z. X, S.-X. L, Y.-H. S, M.-S. H, S.-G. L, et al. Prevalence and course of schizophrenia in a Chinese rural area. Aust N Z J Psychiatry. 2003;37:452–457.

137. Ran M-S, Chan CL-W, Xiang M-Z, Wu Q-H. Suicide attempts among patients with psychosis in a Chinese rural community. Acta Psychiatr Scand. 2003;107:430—435.

138. Räsänen S, Veijola J, Hakko H, Joukamaa M, Isohanni M. Gender differences in incidence and age at

onset of DSM-III-R schizophrenia. Preliminary results of the Northern Finland 1966 birth cohort study. Schizophr Res. 1999;37:197—198.

139. Rastam M, Gillberg C, Garton M. Anorexia nervosa in a Swedish urban region. A population-based study. Br J Psychiatry. 1989;155:642–646.

140. Rautio N, Käkelä J, Nordström T, Miettunen J, Keinänen-Kiukaanniemi S, Ala-Mursula L, et al. Prognosis of schizophrenia spectrum disorder may not be predetermined during early development--the Northern Finland Birth Cohort 1966. Schizophr Res. 2016;173:62–68.

141. Reardon ML, Burns AB, Preist R, Sachs-Ericsson N, Lang AR. Alcohol use and other psychiatric disorders in the formerly homeless and never homeless: prevalence, age of onset, comorbidity, temporal sequencing, and service utilization. Subst Use Misuse. 2003;38:601—644.

142. Reed V, Wittchen HU. DSM-IV panic attacks and panic disorder in a community sample of adolescents and young adults: How specific are panic attacks? J Psychiatr Res. 1998;32:335–345.

143. Rey JM. The epidemiologic catchment area (ECA) study: implications for Australia. Med J Aust. 1992;156:200—203.

144. Riala K, Hakko H, Isohanni M, Pouta A, Räsänen P. Is initiation of smoking associated with the prodromal phase of schizophrenia? J Psychiatry Neurosci. 2005. 2005.

145. Ritter PS, Höfler M, Wittchen HU, Lieb R, Bauer M, Pfennig A, et al. Disturbed sleep as risk factor for the subsequent onset of bipolar disorder - Data from a 10-year prospective-longitudinal study among adolescents and young adults. J Psychiatr Res. 2015;68:76–82.

146. Rodgers S, Ajdacic-Gross V, Kawohl W, Müller M, Rössler W, Hengartner MP, et al. Comparing two basic subtypes in OCD across three large community samples: a pure compulsive versus a mixed obsessive-compulsive subtype. Eur Arch Psychiatry Clin Neurosci. 2015;265:719–734.

147. Roest AM, de Vries YA, Lim CCW, Wittchen HU, Stein DJ, Adamowski T, et al. A comparison of DSM-5 and DSM-IV agoraphobia in the World Mental Health Surveys. Depress Anxiety. 2019;36:499–510.

148. Ruscio AM, Hallion LS, Lim CCW, Aguilar-Gaxiola S, Al-Hamzawi A, Alonso J, et al. Cross-sectional comparison of the epidemiology of DSM-5 generalized anxiety disorder across the globe. JAMA Psychiatry. 2017;74:465–475.

149. Sala R, Goldstein BI, Wang S, Flórez-Salamanca L, Iza M, Blanco C. Increased prospective health service use for depression among adults with childhood onset bipolar disorder. J Pediatr. 2013;163.

150. Schaffer A, Cairney J, Cheung A, Veldhuizen S, Levitt A. Community survey of bipolar disorder in Canada: Lifetime prevalence and illness characteristics. Can J Psychiatry. 2006;51:9–16.

151. Schneier FR, Foose TE, Hasin DS, Heimberg RG, Liu SM, Grant BF, et al. Social anxiety disorder and alcohol use disorder co-morbidity in the national epidemiologic survey on alcohol and related conditions. Psychol Med. 2010. 2010. https://doi.org/10.1017/S0033291709991231.

152. Schneier FR, Johnson J, Hornig CD, Liebowitz MR, Weissman MM. Social phobia. Comorbidity and morbidity in an epidemiologic sample. Arch Gen Psychiatry. 1992;49:282—288.

153. Schuckit MA, Smith TL. Onset and course of alcoholism over 25 years in middle class men. Drug Alcohol Depend. 2011;113:21–28.

154. Scott KM, Lim CCW, Hwang I, Adamowski T, Al-Hamzawi A, Bromet E, et al. The cross-national epidemiology of DSM-IV intermittent explosive disorder. Psychol Med. 2016;46:3161–3172.

155. Silove D, Alonso J, Bromet E, Gruber M, Sampson N, Scott K, et al. Pediatric-onset and adult-onset separation anxiety disorder across countries in the world mental health survey. Am J Psychiatry. 2015;172:647–656.

156. Singh SP, Lee AS. Conversion disorders in Nottingham: Alive, but not kicking. J Psychosom Res. 1997;43:425–430.

157. Slutske WS, Piasecki TM, Deutsch AR, Statham DJ, Martin NG. Telescoping and gender differences in the time course of disordered gambling: evidence from a general population sample. Addiction. 2015;110:144–151.

158. Sorenson SB, Rutter CM, Aneshensel CS. Depression in the Community: An Investigation Into Age of Onset. J Consult Clin Psychol. 1991;59:541–546.

159. Stefánsson JG, Líndal E, Gudmundsdóttir Á, Björnsson JK. Alcohol abuse and dependence in an Icelandic cohort as estimated with the diagnostic interview schedule. Nord J Psychiatry. 1996. 1996. https://doi.org/10.3109/08039489609081412.

160. Stein DJ, Lim CCW, Roest AM, de Jonge P, Aguilar-Gaxiola S, Al-Hamzawi A, et al. The cross-national epidemiology of social anxiety disorder: Data from the World Mental Health Survey Initiative. BMC Med. 2017;15:143.

161. Stein DJ, Seedat S, Herman A, Moomal H, Heeringa SG, Kessler RC, et al. Lifetime prevalence of psychiatric disorders in South Africa. Br J Psychiatry. 2008. 2008. https://doi.org/10.1192/bjp.bp.106.029280.

162. Steinhausen HC, Jakobsen H. Incidence rates of treated mental disorders in childhood and adolescence in a complete nationwide birth cohort. J Clin Psychiatry. 2019;80.

163. STINSON FS, DAWSON DA, PATRICIA CHOU S, SMITH S, GOLDSTEIN RB, JUNE RUAN W, et al. The epidemiology of DSM-IV specific phobia in the USA: results from the National Epidemiologic Survey on Alcohol and Related Conditions. Psychol Med. 2007;37:1047–1059.

164. Suliman S, Seedat S, Williams DR, Stein DJ. Predictors of transitions across stages of alcohol use and alcohol-use disorders in South Africa. J Stud Alcohol Drugs. 2010. 2010. https://doi.org/10.15288/jsad.2010.71.695.

165. Suvisaari JM, Haukka JK, Tanskanen AJ, Lönnqvist JK. Decline in the Incidence of Schizophrenia in Finnish Cohorts Born From 1954 to 1965. Arch Gen Psychiatry. 1999;56:733–740.

166. Suvisaari JM, Haukka JK, Tanskanen AJ, Lönnqvist JK. Decreasing seasonal variation of births in schizophrenia. Psychol Med. 2000;30:315–324.

167. Svensson AC, Lichtenstein P, Sandin S, Öberg S, Sullivan PF, Hultman CM. Familial aggregation of schizophrenia: The moderating effect of age at onset, parental immigration, paternal age and season of birth. Scand J Public Health. 2012. 2012. https://doi.org/10.1177/1403494811420485.

168. Swendsen J, Burstein M, Case B, Conway KP, Dierker L, He J, et al. Use and abuse of alcohol and illicit drugs in US adolescents: Results of the National Comorbidity Survey-Adolescent Supplement. Arch Gen Psychiatry. 2012;69:390–398.

169. Tai Y-M, Gau C-S, Gau SS-F, Chiu H-W. Prediction of ADHD to anxiety disorders: an 11-year national insurance data analysis in Taiwan. J Atten Disord. 2013;17:660–669.

170. Taylor JY, Caldwell CH, Baser RE, Faison N, Jackson JS. Prevalence of eating disorders among blacks in the national survey of American life. Int J Eat Disord. 2007;40:S10--S14.

171. Thorup A, Petersen L, Jeppesen P, Ohlenschlæger J, Christensen T, Krarup G, et al. Gender differences in young adults with first-episode schizophrenia spectrum disorders at baseline in the Danish OPUS study. J Nerv Ment Dis. 2007;195:396–405.

172. Tibi L, Van Oppen P, Aderka IM, Van Balkom AJLM, Batelaan NM, Spinhoven P, et al. An admixture analysis of age of onset in agoraphobia. J Affect Disord. 2015;180:112–115.

173. Tolin DF, Meunier SA, Frost RO, Steketee G. Course of compulsive hoarding and its relationship to life events. Depress Anxiety. 2010;27:829–838.

174. Udo T, Grilo CM. Prevalence and Correlates of DSM-5-Defined Eating Disorders in a Nationally Representative Sample of U.S. Adults. Biol Psychiatry. 2018;84:345—354.

175. Ullman VZ, Levine SZ, Reichenberg A, Rabinowitz J. Real-world premorbid functioning in schizophrenia and affective disorders during the early teenage years: A population-based study of school grades and teacher ratings. Schizophr Res. 2012. 2012. https://doi.org/10.1016/j.schres.2012.01.021.

176. Vaingankar JA, Rekhi G, Subramaniam M, Abdin E, Chong SA. Age of onset of life-time mental disorders and treatment contact. Soc Psychiatry Psychiatr Epidemiol. 2013;48:835–843.

177. Vande Voort JL, He J-P, Jameson ND, Merikangas KR. Impact of the DSM-5 Attention-Deficit/Hyperactivity Disorder Age-of-Onset Criterion in the US Adolescent Population. J Am Acad Child Adolesc Psychiatry. 2014;53:736–744.

178. Verdura Vizcaíno EJ, Fernández-Navarro P, Petry N, Rubio G, Blanco C. Differences between early-onset pathological gambling and later-onset pathological gambling: Data from the National Epidemiologic Survey on Alcohol and Related Conditions (NESARC). Addiction. 2014;109:807–813.

179. Viana MC, Andrade LH. Lifetime Prevalence, Age and Gender Distribution and Age-of-Onset of Psychiatric Disorders in the São Paulo Metropolitan Area, Brazil: Results from the São Paulo Megacity Mental Health Survey. Rev Bras Psiquiatr. 2013;34:249–260.

180. Vila-Rodriguez F, Ochoa S, Autonell J, Usall J, Haro JM. Complex interaction between symptoms, social factors, and gender in social functioning in a community-dwelling sample of schizophrenia. Psychiatr Q. 2011;82:261–274.

181. Posada-Villa J, Lim CCW, de Girolamo G, Benjet C, Demyttenaere K, Alonso J, et al. The cross-national epidemiology of specific phobia in the World Mental Health Surveys. Psychol Med. 2017;47:1744–1760.

182. Weissman MM, Leaf PJ, Tischler GL, Blazer DG, Karno M, Bruce ML, et al. Affective disorders in five United States communities. Psychol Med. 1988;18:141–153.

183. Weissman MM, Bland RC, Canino GJ, Faravelli C, Greenwald S, Hwu H-G, et al. Cross-National Epidemiology of Major Depression and Bipolar Disorder. JAMA. 1996;276:293–299.

184. Weissman MM, Bland R, Joyce PR, Newman S, Wells JE, Wittchen HU. Sex differences in rates of depression: cross-national perspectives. J Affect Disord. 1993;29:77–84.

185. Weissman MM, Bland RC, Canino GJ, Greenwald S, Lee CKK, Newman SC, et al. The cross-national epidemiology of social phobia: a preliminary report. Int Clin Psychopharmacol. 1996;11:9–14.

186. Wells JE, McGee MA, Scott KM, Oakley Browne MA. Bipolar disorder with frequent mood episodes in the New Zealand Mental Health Survey. J Affect Disord. 2010;126:65–74.

187. WHO International Consortium in Psychiatric Epidemiology. Cross-national comparisons of the prevalences and correlates of mental disorders WHO International Consortium in Psychiatric Epidemiology. Bull World Health Organ. 2000;78:413–426.

188. Wiborg JF, Gieseler D, Fabisch AB, Voigt K, Lautenbach A, Löwe B. Suicidality in primary care patients with somatoform disorders. Psychosom Med. 2013;75:800–806.

189. Williams LJ, Jacka FN, Pasco JA, Coulson CE, Quirk SE, Stuart AL, et al. The prevalence and age of onset of psychiatric disorders in Australian men. Aust N Z J Psychiatry. 2016;50:678–684.

190. Wittchen H-U, Nocon A, Beesdo K, Pine DS, Höfler M, Lieb R, et al. Agoraphobia and Panic. Psychother Psychosom. 2008;77:147–157.

191. Wittchen HU, Stein MB, Kessler RC. Social fears and social phobia in a community sample of

adolescents and young adults: prevalence, risk factors and co-morbidity. Psychol Med. 1999;29:309—323.

192. Woo J, Hong JP, Cho SJ, Lee JY, Joen HJ, Kim BS, et al. Bidirectional Association between First-Episode Panic Disorder and Major Depressive Disorder in a Nationwide General Population Survey in Korea. J Korean Med Sci. 2019;34:e181.

193. Yin H, Xu G, Tian H, Yang G, Wardenaar KJ, Schoevers RA. The prevalence, age-of-onset and the correlates of DSM-IV psychiatric disorders in the Tianjin Mental Health Survey (TJMHS). Psychol Med. 2018;48:473–487.

194. Yoshimasu K, Kawakami N. Epidemiological aspects of intermittent explosive disorder in Japan; prevalence and psychosocial comorbidity: Findings from the World Mental Health Japan Survey 2002-2006. Psychiatry Res. 2011;186:384–389.

195. Young SYN, Hansen CJ, Gibson RL, Ryan MAK. Risky Alcohol Use, Age at Onset of Drinking, and Adverse Childhood Experiences in Young Men Entering the US Marine Corps. Arch Pediatr Adolesc Med. 2006;160:1207.

196. Zvolensky MJ, Bernstein A, Sachs-Ericsson N, Schmidt NB, Buckner JD, Bonn-Miller MO. Lifetime associations between cannabis, use, abuse, and dependence and panic attacks in a representative sample. J Psychiatr Res. 2006;40:477–486.

197. Azagba S, Asbridge M. Age of first use, current marijuana use and driving after use among Canadian high school students. Addict Behav. 2019;90:329—333.

198. Baggio S, Studer J, Mohler-Kuo M, Daeppen JB, Gmel G. Profiles of drug users in Switzerland and effects of early-onset intensive use of alcohol, tobacco and cannabis on other illicit drug use. Swiss Med Wkly. 2013;143.

199. Best D, Rawaf S, Rowley J, Floyd K, Manning V, Strang J. Ethnic and gender differences in drinking and smoking among London adolescents. Ethn Heal. 2001;6:51–57.

200. Cheng HG, Anthony JC, Huang Y, Lee S, Liu Z, He Y. Childhood physical punishment and the onset of drinking problems : Evidence from metropolitan China. Drug Alcohol Depend. 2011;118:31–39.

201. Cheng HG, Anthony JC. Female-male differences in alcohol dependence levels: Evidence on newly incident adolescent and young-adult drinkers in the United States, 2002-2014. Int J Methods Psychiatr Res. 2018;27:e1717.

202. Ciairano S, Molinengo G, Bonino S, Miceli R. Age of initiation with different substances and relationships with resources and vulnerabilities : A cross-national study. 2009;6:666–684.

203. de Graaf R, Radovanovic M, van Laar M, Fairman B, Degenhardt L, Aguilar-Gaxiola S, et al. Early cannabis use and estimated risk of later onset of depression spells: Epidemiologic evidence from the population-based World Health Organization World Mental Health Survey Initiative. Am J Epidemiol. 2010;172:149—159.

204. Demant D, Hides L, Kavanagh DJ, White KM, Winstock AR, Ferris J. Differences in substance use between sexual orientations in a multi-country sample: findings from the Global Drug Survey 2015. J Public Health (Oxf). 2017;39:532—541.

205. Diekstra RF. The epidemiology of suicide and parasuicide. Acta Psychiatr Scand Suppl. 1993;371:9—20.

206. Duke DC, Bodzin DK, Tavares P, Geffken GR, Storch EA. The phenomenology of hairpulling in a community sample. J Anxiety Disord. 2009;23:1118—1125.

207. González-Chica DA, Licinio J, Musker M, Wong M, Bowden J, Hay P, et al. Bullying and sexual abuse and their association with harmful behaviours, antidepressant use and health-related quality of life in adulthood: a population-based study in South Australia. BMC Public Health. 2019;19:26.

208. Kaestle CE. Age of smoking milestones: longitudinal inconsistencies and recanting. J Adolesc Health. 2015;56:382—388.

209. Kandel DB, Yamaguchi K, Chen K. Stages of progression in drug involvement from adolescence to adulthood: further evidence for the gateway theory. J Stud Alcohol. 1992;53:447—457.

210. Karam EG, Sampson N, Itani L, Andrade LH, Borges G, Chiu WT, et al. Under-reporting bipolar disorder in large-scale epidemiologic studies. J Affect Disord. 2014;159:147–154.

211. Kessler RC, Coccaro EF, Fava M, McLaughlin KA. The Phenomenology and Epidemiology of Intermittent Explosive Disorder. Oxford Handb. Impuls. Control Disord., 2012.

212. Lahey BB, Goodman SH, Waldman ID, Bird H, Canino G, Jensen P, et al. Relation of age of onset to the type and severity of child and adolescent conduct problems. J Abnorm Child Psychol. 1999;27:247—260.

213. Lintonen T, Rimpelä M, Ahlström S, Rimpelä A, Vikat A. Trends in drinking habits among Finnish adolescents from 1977 to 1999. Addiction. 2000;95:1255—1263.

214. Lo CC. Timing of drinking initiation: A trend study predicting drug use among high school seniors. J Drug Issues. 2000. 2000. https://doi.org/10.1177/002204260003000303.

215. Manna G, Casiglia A, Farad P. Substances use and perception among adolescents in Italy: Findings from an exploratory study. BPA-Applied Psychol Bull (Bollettino Di Psicol Appl. 2010. 2010.

216. Martins-Oliveira JG, Kawachi I, Paiva PCP, Paiva HN de, Pordeus IA, Zarzar PM. Correlates of binge drinking among Brazilian adolescents. Cien Saude Colet. 2018;23:3445—3452.

217. McGrath JJ, Saha S, Al-Hamzawi AO, Alonso J, Andrade L, Borges G, et al. Age of Onset and Lifetime Projected Risk of Psychotic Experiences: Cross-National Data From the World Mental Health Survey. Schizophr Bull. 2016;42:933–941.

218. Monshouwer K, Smit F, de Graaf R, van Os J, Vollebergh W. First cannabis use: does onset shift to younger ages? Findings from 1988 to 2003 from the Dutch National School Survey on Substance Use. Addiction. 2005;100:963—970.

219. Mutumba M, Schulenberg JE. Tobacco and Alcohol Use Among Youth in Low and Middle Income Countries: A Multi-Country Analysis on the Influence of Structural and Micro-Level Factors. Subst Use Misuse. 2019;54:396—411.

220. Najman JM, Plotnikova M, Horwood J, Silins E, Fergusson D, Patton GC, et al. Does adolescent heavier alcohol use predict young adult aggression and delinquency? Parallel analyses from four Australasian cohort studies. Aggress Behav. 2019;45:427—436.

221. Nigg CR, Anderson JK, Troumbley R, Alam MM, Keller S. Recent trends in adolescent alcohol use in Hawai'i: 2005-2011. Hawaii J Med Public Health. 2013;72:92—98.

222. Pacek LR, Storr CL, Mojtabai R, Green KM, La Flair LN, Alvanzo AAH, et al. Comorbid alcohol dependence and anxiety disorders: A national survey. J Dual Diagn. 2013. 2013. https://doi.org/10.1080/15504263.2013.835164.

223. Parra GR, O'Neill SE, Sher KJ. Reliability of self-reported age of substance involvement onset. Psychol Addict Behav. 2003;17:211—218.

224. Reingle Gonzalez JM, Salas-Wright CP, Connell NM, Jetelina KK, Clipper SJ, Businelle MS. The long-term effects of school dropout and GED attainment on substance use disorders. Drug Alcohol Depend. 2016;158:60—66.

225. Resnick MD, Bearman PS, Blum RW, Bauman KE, Harris KM, Jones J, et al. Protecting adolescents from harm. Findings from the National Longitudinal Study on Adolescent Health. JAMA 278(10)823-32, 1997 Sep 10. 1997. 1997.

226. Roderick E, Penney J, Murrells T, Dargan PI, Norman IJ. Epidemiology of adolescent substance use in Norfolk schools. QJM. 2018;111:699—706.

227. Soueif MI, Yunis FA, Youssuf GS, Moneim HA, Taha HS, Sree OA, et al. The use of psychoactive substances among Egyptian males working in the manufacturing industries. Drug Alcohol Depend. 1988;21:217—229.

228. Staff J, Whichard C, Siennick S, Maggs J. EARLY LIFE RISKS, ANTISOCIAL TENDENCIES, AND PRETEEN DELINQUENCY. Criminol an Interdiscip J. 2015;53:677—701.

229. Storr CL, Zhou H, Liang K-Y, Anthony JC. Empirically derived latent classes of tobacco dependence syndromes observed in recent-onset tobacco smokers: epidemiological evidence from a national probability sample survey. Nicotine Tob Res. 2004;6:533—545.

230. Strunin L, Díaz-Martínez A, Díaz-Martínez LR, Heeren T, Chen C, Winter M, et al. Age of Onset, Current Use of Alcohol, Tobacco or Marijuana and Current Polysubstance Use Among Male and Female Mexican Students. Alcohol Alcohol. 2017;52:564—571.

231. Uppal GS, Babst D V, Schmeidler J. Assessing age-of-onset data on substance use among New York State public secondary school students. Am J Drug Alcohol Abuse. 1977;4:505—515.

232. Van Der Vorst H, Vermulst AA, Meeus WHJ, Deković M, Engels RCME. Identification and Prediction of Drinking Trajectories in Early and Mid-Adolescence. J Clin Child Adolesc Psychol. 2009;38:329–341.

233. Vieira DL, Ribeiro M, Laranjeira R. Evidence of association between early alcohol use and risk of later problems. Rev Bras Psiquiatr. 2007;29:222–227.

234. Wallinius M, Delfin C, Billstedt E, Nilsson T, Anckarsäter H, Hofvander B. Offenders in emerging adulthood: School maladjustment, childhood adversities, and prediction of aggressive antisocial behaviors. Law Hum Behav. 2016;40:551—563.

235. Wilson DM, Killen JD, Hayward C, Robinson TN, Hammer LD, Kraemer HC, et al. Timing and rate of sexual maturation and the onset of cigarette and alcohol use among teenage girls. Arch Pediatr Adolesc Med 148(8)789-95, 1994 Aug. 1994. 1994.

236. Fiestas F, Piazza M. Prevalencia de vida y edad de inicio de trastornos mentales en el Perú urbano: Resultados del estudio mundial de salud mental, 2005. Rev Perú Med Exp Salud Publica. 2014;31:39–47.

237. Haro JM, Palacín C, Vilagut G, Martínez M, Bernal M, Luque I, et al. Prevalence of mental disorders and associated factors: Results from the ESEMeD-Spain study. Med Clin (Barc). 2006;126:445–451.

238. Paixão C, Matias D, Alencar I, Nunes M, Sales P, Veiga PHA. [Analysis of the prevalence of psychic disorders in the metropolitan region of Recife]. Cien Saude Colet;14:261–266.

239. Abdin E, Subramaniam M, Vaingankar JA, Chong SA. The Role of Sociodemographic Factors in the Risk of Transition from Alcohol Use to Disorders and Remission in Singapore. Alcohol Alcohol. 2013;49:103–108.

240. Åberg MAI, Torén K, Nilsson M, Henriksson M, Kuhn HG, Nyberg J, et al. Nonpsychotic Mental Disorders in Teenage Males and Risk of Early Stroke: A Population-Based Study. Stroke. 2016;47:814–821.

241. Acarturk C, Smit F, de Graaf R, van Straten A, ten Have M, Cuijpers P. Economic costs of social phobia: A population-based study. J Affect Disord. 2009;115:421–429.

242. Afzali MH, Sunderland M, Batterham PJ, Carragher N, Slade T. Trauma characteristics, post-traumatic symptoms, psychiatric disorders and suicidal behaviours: Results from the 2007 Australian National Survey of Mental Health and Wellbeing. Aust N Z J Psychiatry. 2017;51:1142–1151.

243. Agosti V, Stewart JW. Hypomania with and without Dysphoria: Comparison of comorbidity and

clinical characteristics of respondents from a national community sample. J Affect Disord. 2008;108:177–182.

244. Ahmed AO, Green BA, Mccloskey MS, Berman ME. Latent structure of intermittent explosive disorder in an epidemiological sample. J Psychiatr Res. 2010;44:663–672.

245. Alaräisänen A, Miettunen J, Lauronen E, Räsänen P, Isohanni M. Good school performance is a risk factor of suicide in psychoses: A 35-year follow up of the Northern Finland 1966 Birth Cohort. Acta Psychiatr Scand. 2006;114:357–362.

246. Albor YC, Benjet C, Méndez E, Medina-Mora ME. Persistence of specific phobia from adolescence to early adulthood: Longitudinal follow-up of the mexican adolescent mental health survey. J Clin Psychiatry. 2017;78:340–346.

247. Alghzawi H. Probability and correlates of nicotine dependence among smokers with and without major depressive disorder: Results from the national epidemiology survey on alcohol and related conditions. Perspect Psychiatr Care. 2018;54:354—364.

248. Allen KL, Byrne SM, Oddy WH, Schmidt U, Crosby RD. Risk factors for binge eating and purging eating disorders: Differences based on age of onset. Int J Eat Disord. 2014;47:802–812.

249. Alonso J, Jonge P De, Lim CCW, Aguilar-gaxiola S, Bruffaerts R, Caldas-de-almeida JM, et al. Association between mental disorders and subsequent adult onset asthma. J Psychiatr Res Vol59 2014, Pp 179-188. 2014;59.

250. Andrade L, Eaton WW, Chilcoat HD. Lifetime co-morbidity of panic attacks and major depression in a population-based study: age of onset. Psychol Med. 1996;26:991—996.

251. Andreasen NC. Vulnerability to mental illnesses: gender makes a difference, and so does providing good psychiatric care. Am J Psychiatry. 2005;162:211—213.

252. Angst J. Treated versus untreated major depressive episodes. Psychopathology. 1998;31:37—44.

253. Angst J, Gamma A, Rössler W, Ajdacic V, Klein DN. Long-term depression versus episodic major depression: Results from the prospective Zurich study of a community sample. J Affect Disord. 2009;115:112–121.

254. Angst J. European Long-term Followup Studies of Schizophrenia. Schizophr Bull. 2012;14:501–513.

255. Angst J, Paksarian D, Cui L, Merikangas KR, Hengartner MP, Ajdacic-Gross V, et al. The epidemiology of common mental disorders from age 20 to 50: Results from the prospective Zurich cohort Study. Epidemiol Psychiatr Sci. 2016;25:24–32.

256. Bacon S, Paternoster R, Brame R. Understanding the relationship between onset age and subsequent offending during adolescence. J Youth Adolesc. 2009;38:301—311.

257. Baldwin JM, Stogner JM, Miller BL. It ’ s five o ’ clock somewhere : An examination of the association between happy hour drinking and negative consequences. Subst Abus Treat Prev Policy Vol9 2014, ArtID 17. 2014;9:1–12.

258. Barker D, Coid JW, Jones PB, Stamps R, Cowden F, Yang M, et al. Psychoses, ethnicity and socio-economic status. Br J Psychiatry. 2008;193:18–24.

259. Bauer M, Glenn T, Alda M, Aleksandrovich MA, Andreassen OA, Angelopoulos E, et al. Solar insolation in springtime influences age of onset of bipolar I disorder. Acta Psychiatr Scand. 2017;136:571–582.

260. Bauermeister JJ, Bird HR, Shrout PE, Chavez L, Ramírez R, Canino G. Short-term persistence of DSM-IV ADHD diagnoses: influence of context, age, and gender. J Am Acad Child Adolesc Psychiatry. 2011;50:554—562.

261. Baumeister SE, Tossmann P. Association between early onset of cigarette, alcohol and cannabis use

and later drug use patterns: an analysis of a survey in European metropolises. Eur Addict Res. 2005;11:92—98.

262. Behrendt S, Wittchen HU, Höfler M, Lieb R, Beesdo K. Transitions from first substance use to substance use disorders in adolescence: Is early onset associated with a rapid escalation? Drug Alcohol Depend. 2009;99:68–78.

263. Behrendt S, Beesdo-baum K, Höfler M, Perkonigg A, Bühringer G, Lieb R, et al. The relevance of age at first alcohol and nicotine use for initiation of cannabis use and progression to cannabis use disorders. Drug Alcohol Depend. 2012;123:48–56.

264. Belik SL, Cox BJ, Stein MB, Asmundson GJG, Sareen J. Traumatic events and suicidal behavior: Results from a national mental health survey. J Nerv Ment Dis. 2007;195:342–349.

265. Bernstein A, Zvolensky MJ, Sachs-Ericsson N, Schmidt NB, Bonn-Miller MO. Associations between age of onset and lifetime history of panic attacks and alcohol use, abuse, and dependence in a representative sample. Compr Psychiatry. 2006;47:342–349.

266. BHUGRA D, MALLETT R, RUDGE S, LEFF J, DER G, CORRIDAN B. Incidence and outcome of schizophrenia in Whites, African-Caribbeans and Asians in London. Psychol Med. 2002;27:791–798.

267. Bilevicius E, Sommer JL, Asmundson GJG, El-Gabalawy R. Associations of PTSD, chronic pain, and their comorbidity on cannabis use disorder: Results from an American nationally representative study. Depress Anxiety. 2019;36:1036—1046.

268. Birrell L, Newton NC, Teesson M, Tonks Z, Slade T. Anxiety disorders and first alcohol use in the general population. Findings from a nationally representative sample. J Anxiety Disord. 2015;31:108–113.

269. Blobaum EM, Anderson JF. The Impact of Exposure and Perceived Disapproval of Underage Drinking. Crim Justice Stud. 2006. 2006. https://doi.org/10.1080/14786010600764559.

270. Bogren M, Mattisson C, Horstmann V, Bhugra D, Munk-Jørgensen P, Nettelbladt P. Lundby revisited: First incidence of mental disorders 1947-1997. Aust N Z J Psychiatry. 2007;41:178–186.

271. Bogren M, Mattisson C, Isberg P, Munk-jørgensen P. Incidence of psychotic disorders in the 50 year follow up of the Lundby population. Aust New Zeal J Psychiatry Vol44(1), 2010, Pp 31-39. 2010. 2010.

272. Borga P, Widerlov B, Cg S, Social CJ. Social conditions in a total population with long-term functional psychosis in three different areas of Stockholm Countv. Acta Psychiatr Scand 85(6)465-73, 1992 Jun. 1992. 1992.

273. Borges G, Medina Mora-Icaza ME, Benjet C, Lee S, Lane M, Breslau J. Influence of mental disorders on school dropout in Mexico Suggested citation. vol. 30. 2011.

274. Boschloo L, Vogelzangs N, Smit JH, Van Den Brink W, Veltman DJ, Beekman ATF, et al. Comorbidity and risk indicators for alcohol use disorders among persons with anxiety and/or depressive disorders: Findings from the netherlands study of depression and anxiety (NESDA). J Affect Disord. 2011;131:233–242.

275. Bourdon KH, Rae DS, Locke BZ, Narrow WE, Regier DA. Estimating the prevalence of mental disorders in U.S. adults from the Epidemiologic Catchment Area Survey. Public Health Rep. 1992;107:663–668.

276. Breslau N, Novak SP, Kessler RC. Psychiatric disorders and stages of smoking. Biol Psychiatry. 2004;55:69—76.

277. Breslau J, Aguilar-Gaxiola S, Borges G, Castilla-Puentes RC, Kendler KS, Medina-Mora M-E, et al. Mental disorders among English-speaking Mexican immigrants to the US compared to a national sample of Mexicans. Psychiatry Res. 2007;151:115—122.

278. Breslau J, Lane M, Sampson N, C R. NIH Public Access. Psychiatry (Abingdon). 2009;42:708–716.

279. Brezo J, Paris J, Barker ED, Tremblay R, Vitaro F, Zoccolillo M, et al. Natural history of suicidal behaviors in a population-based sample of young adults. Psychol Med. 2007;37:1563—1574.

280. Bromet EJ, Havenaar JM, Tintle N, Kostyuchenko S, Kotov R, Gluzman S. Suicide ideation, plans and attempts in Ukraine: findings from the Ukraine World Mental Health Survey. Psychol Med. 2007;37:807—819.

281. Brower KJ, Perron BE. Prevalence and correlates of withdrawal-related insomnia among adults with alcohol dependence: Results from a national survey. Am J Addict. 2010. 2010. https://doi.org/10.1111/j.1521-0391.2010.00035.x.

282. Browne MAO, Scott KM, Kokaua J, Wells JE, McGee MA, Baxter J. Prevalence, interference with life and severity of 12 month DSM-IV disorders in Te Rau Hinengaro: The New Zealand Mental Health Survey. Aust N Z J Psychiatry. 2006;40:845–854.

283. Bruffaerts R, Bonnewyn A, Demyttenaere K. Delays in seeking treatment for mental disorders in the Belgian general population. Soc Psychiatry Psychiatr Epidemiol. 2007;42:937–944.

284. Bruffaerts R, Caldas-de-almeida JM. HHS Public Access. 2017;73:150–158.

285. Bulayeva KB. Overview of genetic-epidemiological studies in ethnically and demographically diverse isolates of Dagestan, Northern Caucasus, Russia. Croat Med J. 2006;47:641–648.

286. Buller R, Maier W, Goldenberg IM, Lavoir PW, Benkert O. Chronology of panic and avoidance, age of onset in panic disorder, and prediction of treatment response - A report from the cross-national collaborative panic study. Eur Arch Psychiatry Clin Neurosci. 1991;240:163–168.

287. Burke KC, Burke JD, Rae DS, Regier DA. Comparing age at onset of major depression and other psychiatric disorders by birth cohorts in five US community populations. Arch Gen Psychiatry. 1991;48:789—795.

288. Burke JD, Burke KC, Rae DS. Increased rates of drug abuse and dependence after onset of mood or anxiety disorders in adolescence. Hosp Community Psychiatry. 1994. 1994. https://doi.org/10.1176/ps.45.5.451.

289. Burns LE. The epidemiology of fears and phobias in general practice. J Int Med Res. 1980;8 Suppl 3:1—7.

290. Burt SA, Hopwood CJ. A Comparison of Two Different Approaches to Characterizing the Heterogeneity Within Antisocial Behavior: Age-of-Onset versus Behavioral Sub-Types. J Pers Disord. 2010;24:272–283.

291. Byers AL, Covinsky KE, Neylan TC, Yaffe K. Chronicity of posttraumatic stress disorder and risk of disability in older persons. JAMA Psychiatry. 2014;71:540—546.

292. Cantor-Graae E, Pedersen CB. Risk for schizophrenia in intercountry adoptees: A Danish population-based cohort study. J Child Psychol Psychiatry Allied Discip. 2007;48:1053–1060.

293. Capone C, Wood MD. Density of Familial Alcoholism and Its Effects on Alcohol Use and Problems in College Students. Alcohol Clin Exp Res Vol32(8), 2008, Pp 1451-1458. 2008;32:1451–1458.

294. Castagnini AC, Laursen TM, Mortensen PB, Bertelsen A. Family psychiatric morbidity of acute and transient psychotic disorders and their relationship to schizophrenia and bipolar disorder. Psychol Med. 2013;43:2369–2375.

295. Chan SKW, Tang JYM, Lam MML, Chan KKS, Hui CLM, Chen EYH, et al. Executive function in first-episode schizophrenia: A three-year longitudinal study of an ecologically valid test. Schizophr Res. 2010;126:87–92.

296. Chang H-J, Chou K-R, Chiou H-C, Chiu C-H, Tang H-S, Hung T-C, et al. Anxiety, depressive

symptom and suicidal ideation of outpatients with obsessive compulsive disorders in Taiwan. J Clin Nurs. 2010;19:3092–3101.

297. Chapa DAN, Bohrer BK, Forbush KT. Is the diagnostic threshold for bulimia nervosa clinically meaningful? Eat Behav. 2018;28:16–19.

298. Chapman C, Slade T, Hunt C, Teesson M. Delay to first treatment contact for alcohol use disorder. Drug Alcohol Depend. 2015;147:116–121.

299. Chartier MJ, Walker JR, Stein MB. Considering comorbidity in social phobia. Soc Psychiatry Psychiatr Epidemiol. 2003;38:728–734.

300. Chartier KG, Hesselbrock MN, Hesselbrock VM. Alcohol problems in young adults transitioning from adolescence to adulthood: The association with race and gender. Addict Behav. 2011;36:167—174.

301. Chen J, Tsuchiya M, Kawakami N, Furukawa TA. Non-fearful vs. fearful panic attacks: A general population study from the National Comorbidity Survey. J Affect Disord. 2009;112:273–278.

302. Cheng HG, Lopez-Quintero C, Anthony JC. Age of onset or age at assessment—that is the question: Estimating newly incident alcohol drinking and rapid transition to heavy drinking in the United States, 2002–2014. Int J Methods Psychiatr Res. 2018;27.

303. Chong SA, Abdin E, Sherbourne C, Vaingankar J, Heng D, Yap M, et al. Treatment gap in common mental disorders: The Singapore perspective. Epidemiol Psychiatr Sci. 2012;21:195–202.

304. Clark C, Rodgers B, Caldwell T, Power C, Stansfeld S. Childhood and adulthood psychological ill health as predictors of midlife affective and anxiety disorders: the 1958 British Birth Cohort. Arch Gen Psychiatry. 2007;64:668—678.

305. Clemmensen L, Van Os J, Drukker M, Munkholm A, Rimvall MK, Væver M, et al. Psychotic experiences and hyper-theory-of-mind in preadolescence - A birth cohort study. Psychol Med. 2016;46:87–101.

306. Cohen A, Esan O, Morgan C, Patel V, Hutchinson G, Hibben M, et al. The incidence of psychoses in diverse settings, INTREPID (2): a feasibility study in India, Nigeria, and Trinidad. Psychol Med. 2016;46:1923–1933.

307. Colman I, Wadsworth MEJ, Croudace TJ, Jones PB. Forty-year psychiatric outcomes following assessment for internalizing disorder in adolescence. Am J Psychiatry. 2007;164:126–133.

308. Crum RM, Ford DE, Storr CL, Chan Y-F. Association of sleep disturbance with chronicity and remission of alcohol dependence: data from a population-based prospective study. Alcohol Clin Exp Res. 2004;28:1533—1540.

309. Cuijpers P, Smit F, Ten Have M, De Graaf R. Smoking is associated with first-ever incidence of mental disorders: A prospective population-based study. Addiction. 2007;102:1303–1309.

310. A. S. DAVID,"A. MALMBERG, L. BRANDT PA and GL. IQ and risk for schizophrenia : a population-based cohort study. Psychol Med 27(6)1311-23, 1997 Nov. 1997:1311–1323.

311. Dayal P, Balhara YPS. A naturalistic study of predictors of retention in treatment among emerging adults entering first buprenorphine maintenance treatment for opioid use disorders. J Subst Abuse Treat. 2017;80:1–5.

312. de Graaf R, Penninx BWJH, Beekman ATF, Rhebergen D, Spijker J, Lamers F. Course trajectories of unipolar depressive disorders identified by latent class growth analysis. Psychol Med. 2011;42:1383–1396.

313. de Heer EW, Dekker J, van Marwijk HWJ, ten Have M, van der Feltz-Cornelis CM, Beekman ATF, et al. Pain as a risk factor for common mental disorders. Results from the Netherlands Mental Health Survey and Incidence Study-2. Pain. 2017;159:1.

314. Degenhardt L, Hall W, Lynskey M. Testing hypotheses about the relationship between cannabis use and psychosis. Drug Alcohol Depend. 2003;71:37–48.

315. Degenhardt L, Chiu WT, Conway K, Dierker L, Glantz M, Kalaydjian A, et al. Does the ‘gateway’ matter? Associations between the order of drug use initiation and the development of drug dependence in the National Comorbidity Study Replication. Psychol Med. 2009;39:157—167.

316. Degonda M, Wyss M, Angst J. The Zurich Study - XVIII. Obsessive-compulsive disorders and syndromes in the general population. Eur Arch Psychiatry Clin Neurosci. 1993;243:16–22.

317. Demallie DA, Cottler LB, Compton WM. Alcohol abuse and dependence: consistency in reporting of symptoms over ten years. Addiction. 1995;90:615–625.

318. Devanand DP. Dysthymic disorder in the elderly population. Int Psychogeriatrics Vol26(1), 2014, Pp 39-48. 2014:39–48.

319. Dietrich UC, Brooks RT, Beard JR, Heathcote K, Kelly B, Brooks LO. Incidence and Outcomes of Mental Disorders in a Regional Population: The Northern Rivers Mental Health Study. Aust New Zeal J Psychiatry. 2009;40:674–682.

320. Ding K, Chang GA, Southerland R. Age of inhalant first time use and its association to the use of other drugs. J Drug Educ. 2009;39:261—272.

321. Doherty EE, Green KM, Reisinger HS, Ensminger ME. Long-term patterns of drug use among an urban African-American cohort: The role of gender and family. J Urban Heal. 2008;85:250–267.

322. Dube SR, Miller JW, Brown DW, Giles WH, Felitti VJ, Dong M, et al. Adverse childhood experiences and the association with ever using alcohol and initiating alcohol use during adolescence. J Adolesc Health. 2006;38:444.e1—10.

323. Ehlers CL, Slutske WS, Gilder DA, Lau P, Wilhelmsen KC. Age at first intoxication and alcohol use disorders in Southwest California Indians. Alcohol Clin Exp Res. 2006;30:1856—1865.

324. Essau CA, Lewinsohn PM, Seeley JR, Sasagawa S. Gender differences in the developmental course of depression. J Affect Disord. 2010;127:185–190.

325. Evans BE, Greaves-Lord K, Euser AS, Franken IHA, Huizink AC. The relation between hypothalamic-pituitary-adrenal (HPA) axis activity and age of onset of alcohol use. Addiction. 2012;107:312—322.

326. Fairman BJ, Furr-Holden CD, Johnson RM. When Marijuana Is Used before Cigarettes or Alcohol: Demographic Predictors and Associations with Heavy Use, Cannabis Use Disorder, and Other Drug-related Outcomes. Prev Sci. 2019;20:225—233.

327. Fallu J, Brière FN, Janosz M. Latent classes of substance use in adolescent cannabis users : predictors and subsequent substance-related harm. Front Psychiatry Vol5 2014, ArtID 9. 2014;5:1–10.

328. Farrer LA, Florio LP, Bruce ML, Leaf PJ, Weissman MM. Reliability of self-reported age at onset of major depression. J Psychiatr Res. 1989;23:35–47.

329. Fergusson DM, Mcleod GFH, Horwood LJ. Childhood sexual abuse and adult developmental outcomes: Findings from a 30-year longitudinal study in New Zealand. Child Abuse Negl. 2013;37:664–674.

330. Fergusson DM, Horwood LJ, Boden JM, Mulder RT. Impact of a major disaster on the mental health of a well-studied cohort. JAMA Psychiatry. 2014;71:1025–1031.

331. Fischer JA, Najman JM, Plotnikova M, Clavarino AM. Quality of life, age of onset of alcohol use and alcohol use disorders in adolescence and young adulthood: Findings from an Australian birth cohort. Drug Alcohol Rev. 2015;34:388–396.

332. Fleming R, Leventhal H, Glynn K, Ershler J. The role of cigarettes in the initiation and progression of

early substance use. Addict Behav. 1989;14:261—272.

333. Fontenelle LF, Miguel EC, Pittenger C, do Rosario MC, Torres AR, Bloch MH, et al. Dimensional correlates of poor insight in obsessive–compulsive disorder. Prog Neuro-Psychopharmacology Biol Psychiatry. 2011;35:1677–1681.

334. French MT, Balsa AI, Homer JF. The health effects of parental problem drinking on adult children. J Ment Health Policy Econ. 2009;12:55–66.

335. Gaysina D, Hotopf M, Richards M, Colman I, Kuh D, Hardy R. Symptoms of depression and anxiety , and change in body mass index from adolescence to adulthood : results from a British birth cohort. 2011:175–184.

336. Gilder DA, Gizer IR, Lau P, Ehlers CL. Stimulant Dependence and Stimulant-Associated Psychosis. J Addict Med. 2014;8:241–248.

337. Gissler M, Laursen TM, Ösby U, Nordentoft M, Wahlbeck K. Patterns in mortality among people with severe mental disorders across birth cohorts: A register-based study of Denmark and Finland in 1982-2006. BMC Public Health. 2013;13.

338. Glantz MD, Anthony JC, Berglund PA, Dierker L, Kalaydjian A, Merikangas KR, et al. NIH Public Access. 2009;39:1365–1377.

339. Glazebrook C. Incidence of schizophrenia in Nottingham and 992â € ” 94. Schizophrenia. 1997. 1997.

340. Glenn CR, Lanzillo EC, Esposito EC, Santee AC, Nock MK, Auerbach RP. Examining the Course of Suicidal and Nonsuicidal Self-Injurious Thoughts and Behaviors in Outpatient and Inpatient Adolescents. J Abnorm Child Psychol. 2017;45:971–983.

341. Godart N, Chambry J, Falissard B, Rouillon F, Brun-Eberentz A, Guelfi J-D, et al. Severe anorexia nervosa in men: Comparison with severe AN in women and analysis of mortality. Int J Eat Disord. 2012;45:537–545.

342. Goldstein BI, Levitt AJ. Further evidence for a developmental subtype of bipolar disorder defined by age at onset: results from the national epidemiologic survey on alcohol and related conditions. Am J Psychiatry. 2006;163:1633—1636.

343. González-Blanch C, Crespo-Facorro B, Álvarez-Jiménez M, Rodríguez-Sánchez JM, Pelayo-Terán JM, Pérez-Iglesias R, et al. Pretreatment predictors of cognitive deficits in early psychosis. Psychol Med. 2008;38:737–746.

344. Goodwin R, Olfson M. Treatment of panic attack and risk of major depressive disorder in the community. Am J Psychiatry. 2001;158:1146—1148.

345. Grant JD, Scherrer JF, Lynskey MT, Lyons MJ, Eisen SA, Tsuang MT, et al. Adolescent alcohol use is a risk factor for adult alcohol and drug dependence: Evidence from a twin design. Psychol Med. 2006;36:109–118.

346. Gratzer D, Levitan RD, Sheldon T, Toneatto T, Rector NA, Goering P. Lifetime rates of alcoholism in adults with anxiety, depression, or co-morbid depression/anxiety: A community survey of Ontario. J Affect Disord. 2004;79:209–215.

347. Greenfield SF, Pettinati HM, O’Malley S, Randall PK, Randall CL. Gender differences in alcohol treatment: An analysis of outcome from the COMBINE study. Alcohol Clin Exp Res. 2010;34:1803–1812.

348. Gregory A, Poulton R. NIH Public Access. 2009;117:460–466.

349. Grucza RA, Norberg K, Bucholz KK, Bierut LJ. Correspondence between secular changes in alcohol dependence and age of drinking onset among women in the United States. Alcohol Clin Exp Res. 2008;32:1493—1501.

350. Gureje O, Oladeji B, Hwang I, Chiu WT, Kessler RC, Sampson NA, et al. Parental psychopathology and the risk of suicidal behavior in their offspring: results from the World Mental Health surveys. Mol Psychiatry. 2011;16:1221—1233.

351. Häfner H, Behrens S, De Vry J, Gattaz WF. Oestradiol enhances the vulnerability threshold for schizophrenia in women by an early effect on dopaminergic neurotransmission. Evidence from an epidemiological study and from animal experiments. Eur Arch Psychiatry Clin Neurosci. 1991;241:65—68.

352. Häfner H, an der Heiden W. The course of schizophrenia in the light of modern follow-up studies: the ABC and WHO studies. Eur Arch Psychiatry Clin Neurosci. 1999;249 Suppl:14—26.

353. Weingarden H, Renshaw KD. Early and late perceived pubertal timing as risk factors for anxiety disorders in adult women. J Psychiatr Res. 2012;46:1524–1529.

354. Hanna EZ, Grant BF. Parallels to early onset alcohol use in the relationship of early onset smoking with drug use and DSM-IV drug and depressive disorders: findings from the National Longitudinal Epidemiologic Survey. Alcohol Clin Exp Res. 1999;23:513—522.

355. Hanna EZ, Yi HY, Dufour MC, Whitmore CC. The relationship of early-onset regular smoking to alcohol use, depression, illicit drug use, and other risky behaviors during early adolescence: results from the youth supplement to the third national health and nutrition examination survey. J Subst Abuse. 2001;13:265—282.

356. Hansen T, Gylfason A, Kiemeney LA, Strengman E, Werge T, Möller H-J, et al. Copy number variations of chromosome 16p13.1 region associated with schizophrenia. Mol Psychiatry. 2009;16:17–25.

357. Harford TC, Muthén BO. Alcohol use among college students: the effects of prior problem behaviors and change of residence. J Stud Alcohol. 2001;62:306—312.

358. Harford TC, Yi H-Y, Hilton ME. Alcohol abuse and dependence in college and noncollege samples: A ten-year prospective follow-up in a national survey. J Stud Alcohol. 2006;67:803—809.

359. Hart C, De Vet R, Moran P, Hatch SL, Dean K. A UK population-based study of the relationship between mental disorder and victimisation. Soc Psychiatry Psychiatr Epidemiol. 2012;47:1581–1590.

360. Hastrup LH, Haahr UH, Jansen JE, Simonsen E. Determinants of duration of untreated psychosis among first-episode psychosis patients in Denmark: A nationwide register-based study. Schizophr Res. 2018;192:154—158.

361. Hayatbakhsh R, Williams GM, Bor W, Najman JM. Early childhood predictors of age of initiation to use of cannabis : A birth prospective study. Drug Alcohol Rev Vol32(3), 2013, Pp 232-240. 2013:232–240.

362. Hayatbakhsh R, Clavarino A, Williams GM, Najman JM. Maternal and Personal Religious Engagement as Predictors of Early Onset and Frequent Substance Use. Am J Addict Vol23(4), 2014, Pp 363-370. 2014:1–8.

363. Helenius D, Munk-Jørgensen P, Steinhausen HC. Family load estimates of schizophrenia and associated risk factors in a nation-wide population study of former child and adolescent patients up to forty years of age. Schizophr Res. 2012;139:183–188.

364. Hermos JA, Winter MR, Heeren TC, Hingson RW. Early Age-of-onset Drinking Predicts Prescription Drug Misuse Among Teenagers and Young Adults: Results from a National Survey. J Addict Med. 2008;2:22—30.

365. Hill SY, Shen S, Lowers L, Locke J. Factors predicting the onset of adolescent drinking in families at high risk for developing alcoholism. Biol Psychiatry. 2000;48:265–275.

366. Hingson RW, Edwards EM, Heeren T, Rosenbloom D. Age of drinking onset and injuries, motor

vehicle crashes, and physical fights after drinking and when not drinking. Alcohol Clin Exp Res. 2009;33:783—790.

367. Holdcraft LC, Iacono WG. Cross-generational effects on gender differences in psychoactive drug abuse and dependence. Drug Alcohol Depend. 2004;74:147–158.

368. Hong CJ, Pan GM, Tsai SJ. Association study of onset age, attempted suicide, aggressive behavior, and schizophrenia with a serotonin 1B receptor (A-161T) genetic polymorphism. Neuropsychobiology. 2004;49:1–4.

369. Horwath E, Weissman MM. The epidemiology and cross-national presentation of obsessive-compulsive disorder. Psychiatr Clin North Am. 2000;23:493—507.

370. Horwath E, Johnson J, Weissman MM, Hornig CD. The validity of major depression with atypical features based on a community study. J Affect Disord. 1992;26:117—125.

371. Horwath E, Johnson J, Hornig CD. Epidemiology of panic disorder in African-Americans. Am J Psychiatry. 1993;150:465—469.

372. Hwu HG, Chang IH, Yeh EK, Chang CJ, Yeh LL. Major depressive disorder in Taiwan defined by the Chinese diagnostic Interview Schedule. J Nerv Ment Dis. 1996;184:497—502.

373. Hwu HG, Faraone S V., Liu CM, Chen WJ, Liu SK, Shieh MH, et al. Taiwan Schizophrenia Linkage Study? The field study. Am J Med Genet - Neuropsychiatr Genet. 2005;134 B:30–36.

374. Janzing JGE, De Graaf R, Ten Have M, Vollebergh WA, Verhagen M, Buitelaar JK. Familiality of depression in the community; Associations with gender and phenotype of major depressive disorder. Soc Psychiatry Psychiatr Epidemiol. 2009;44:1067–1074.

375. Jenkins MB, Agrawal A, Lynskey MT, Nelson EC, Madden PAF, Bucholz KK, et al. Correlates of alcohol abuse/dependence in early-onset alcohol-using women. Am J Addict. 2011;20:429—434.

376. Joinson C, Heron J, Araya R, Lewis G. Early Menarche and Depressive Symptoms From Adolescence to Young Adulthood in a UK Cohort Carol. J Am Acad Child Adolesc Psychiatry. 2013;52:591-598.e2.

377. Kaelber CT, Regier DA, Rae DS, Schatzberg AF, Narrow WE. Prevalence of anxiety disorders and their comorbidity with mood and addictive disorders. Br J Psychiatry. 2018;173:24–28.

378. Kaltiala-Heino R, Kosunen E, Rimpelä M. Pubertal timing, sexual behaviour and self-reported depression in middle adolescence. J Adolesc. 2003;26:531—545.

379. Karam EG, Salamoun MM, Mneimneh ZN, Fayyad JA, Karam AN, Hajjar R, et al. War and first onset of suicidality: the role of mental disorders. Psychol Med. 2012;42:2109—2118.

380. Keenan K, Wroblewski K, Hipwell A, Stouthamer-loeber M. NIH Public Access. 2010;119:689–698.

381. Keers R, Coid JW, Ullrich S, Cowden F, Stamps R, Kallis C, et al. The Relationship Between Delusions and Violence. JAMA Psychiatry. 2013;70:465.

382. Keller MB, Lavori PW, Wunder J, Beardslee WR, Schwartz CE, Roth J. Chronic Course of Anxiety Disorders in Children and Adolescents. J Am Acad Child Adolesc Psychiatry. 1992;31:595–599.

383. Kelly A, Chan GCK, O'Flaherty M. How important is the context of an adolescent's first alcoholic drink? Evidence that parental provision may reduce later heavy episodic drinking. Eur Addict Res. 2012;18:140—148.

384. Kerr WC, Ye Y, Cherpitel CJ. Racial/Ethnic Disparities in the Risk of Injury Related to the Frequency of Heavy Drinking Occasions. Alcohol Alcohol. 2015;50:573—578.

385. Kessing L V, Mortensen PB, Bolwig TG. Clinical definitions of sensitisation in affective disorder: a case register study of prevalence and prediction. J Affect Disord. 1998;47:31—39.

386. Kessler RC, McGonagle KA, Swartz M, Blazer DG, Nelson CB. Sex and depression in the National Comorbidity Survey I: Lifetime prevalence, chronicity and recurrence. J Affect Disord. 1993;29:85–96.

387. Kessler RC, Sonnega A, Nelson CB, Bromet E. Posttraumatic stress disorder in the National Comorbidity Survey. 1995. 1995.

388. Kessler RC, Stang PE, Wittchen H-U, Ustun TB, Roy-Burne PP, Walters EE. Lifetime Panic-Depression Comorbidity in the National Comorbidity Survey. Arch Gen Psychiatry. 1998;55:801–808.

389. Kessler RC, Olfson M, Berglund PA. Patterns and predictors of treatment contact after first onset of psychiatric disorders. Am J Psychiatry. 1998;155:62—69.

390. Kessler RC, Stang P, Wittchen HU, Stein M, Walters EE. Lifetime co-morbidities between social phobia and mood disorders in the US national comorbidity survey. Psychol Med. 1999. 1999. https://doi.org/10.1017/S0033291799008375.

391. Kessler RC, Aguilar-Gaxiola S, Berglund PA, Caraveo-Anduaga JJ, DeWit DJ, Greenfield SF, et al. Patterns and predictors of treatment seeking after onset of a substance use disorder. Arch Gen Psychiatry. 2001;58:1065—1071.

392. Kessler RC, Hwang I, Labrie R, Petukhova M, Sampson NA, Winters KC, et al. The prevalence and correlates of DSM-IV Pathological Gambling in the National Comorbidity Survey Replication. Psychol Med. 2008;38:1351–1360.

393. Kessler RC, Borges G, Kawakami N, Nock MK, Karam EG, Huang Y, et al. Cross-National Analysis of the Associations among Mental Disorders and Suicidal Behavior: Findings from the WHO World Mental Health Surveys. PLoS Med. 2009;6:e1000123.

394. Kessler RC, Sampson NA, Berglund P, Gruber MJ, Al-Hamzawi A, Andrade L, et al. Anxious and non-anxious major depressive disorder in the World Health Organization World Mental Health Surveys. Epidemiol Psychiatr Sci. 2015;24:210—226.

395. Keyes KM, McLaughlin KA, Vo T, Galbraith T, Heimberg RG. ANXIOUS and AGGRESSIVE: The CO-OCCURRENCE of IED with ANXIETY DISORDERS. Depress Anxiety. 2016;33:101–111.

396. Khalifa N, Von Knorring AL. Tourette syndrome and other tic disorders in a total population of children: Clinical assessment and background. Acta Paediatr Int J Paediatr. 2005;94:1608–1614.

397. Kim JH, Chang SM, Bae JN, Cho SJ, Lee JY, Kim BS, et al. Mental-physical comorbidity in Korean adults: Results from a nationwide general population survey in Korea. Psychiatry Investig. 2016;13:496–503.

398. Kinnunen J, Räsänen P, Timonen M, Järvelin M-R, Miettunen J, Hakko H, et al. Psychiatric admissions at different levels of the national health care services and male criminality: the Northern Finland 1966 Birth Cohort study. Soc Psychiatry Psychiatr Epidemiol. 2002;35:198–201.

399. Kinnunen L, Niemelä M, Hakko H, Miettunen J, Merikukka M, Karttunen V, et al. Brain Injury Psychiatric diagnoses of children affected by their parents' traumatic brain injury: the 1987 Finnish Birth Cohort study Psychiatric diagnoses of children affected by their parents' traumatic brain injury: the 1987 Finnish Birth Cohort study. 2018. 2018. https://doi.org/10.1080/02699052.2018.1470331.

400. Kirkpatrick B, Castle D, Murray RM, Carpenter WT. Risk factors for the deficit syndrome of schizophrenia. Schizophr Bull. 2000. 2000. https://doi.org/10.1093/oxfordjournals.schbul.a033443.

401. Knappe S, Beesdo K, Fehm L, Höfler M, Lieb R, Wittchen H-U. Do parental psychopathology and unfavorable family environment predict the persistence of social phobia? J Anxiety Disord. 2009;23:986—994.

402. Kollins SH, McClernon FJ, Fuemmeler BF. Association between smoking and attention-

deficit/hyperactivity disorder symptoms in a population-based sample of young adults. Arch Gen Psychiatry. 2005;62:1142—1147.

403. Konings M, Konings M, Maharajh HD, Dip Clin Neuro DC. Substance abuse in different school systems in Trinidad and Tobago: A controlled study of children with disabilities and their drug use. Int J Disabil Hum Dev. 2007. 2007. https://doi.org/10.1515/IJDHD.2007.6.1.29.

404. Kratzer L, Hodgins S. Adult outcomes of child conduct problems: A cohort study. J Abnorm Child Psychol. 1997;25:65–81.

405. Kraus L, Augustin R, Kunz-Ebrecht S, Orth B. Drug use patterns and drug-related disorders of cocaine users in a sample of the general population in Germany. Eur Addict Res. 2007;13:116—125.

406. Krueger RF, Caspi A, Moffitt TE, Silva PA, McGee R. Personality traits are differentially linked to mental disorders: A multitrait-multidiagnosis study of an adolescent birth cohort. J Abnorm Psychol. 1996;105:299–312.

407. Kunert HJ. PW01-231 - Attentional dysfunctions in multiple drug abuse: impact of drug clustering, age of onset and comorbid mental disorder. Eur Psychiatry. 2010;25:1659.

408. Laursen TM, Munk-Olsen T, Gasse C. Chronic somatic comorbidity and excess mortality due to natural causes in persons with schizophrenia or bipolar affective disorder. PLoS One. 2011;6.

409. Le Strat Y, Dubertret C, Le Foll B. Impact of age at onset of cannabis use on cannabis dependence and driving under the influence in the United States. Accid Anal Prev. 2015;76:1—5.

410. Leach LS, Butterworth P. The effect of early onset common mental disorders on educational attainment in Australia. Psychiatry Res. 2012;199:51–57.

411. Leão TS, Sundquist J, Johansson LM, Johansson SE, Sundquist K. Incidence of mental disorders in second-generation immigrants in Sweden: A four-year cohort study. Ethn Heal. 2005;10:243–256.

412. Leatherdale ST, Hammond D, Ahmed R. Alcohol, marijuana, and tobacco use patterns among youth in Canada. Cancer Causes Control. 2008;19:361—369.

413. Lee CS, Winters KC, Wall MM. Trajectories of Substance Use Disorders in Youth: Identifying and Predicting Group Memberships. J Child Adolesc Subst Abus Vol19(2), 2010, Pp 135-157. 2010;19:612–627.

414. Legleye S, Obradovic I, Janssen E, Spilka S, Le Nézet O, Beck F. Influence of cannabis use trajectories, grade repetition and family background on the school-dropout rate at the age of 17 years in France. Eur J Public Health. 2010;20:157—163.

415. Leung C-M, Chen EY-H, Lee EH-M, Tang JY-M, Lau WW-Y, Chan SK-W, et al. Clinical and social correlates of duration of untreated psychosis among adult-onset psychosis in Hong Kong Chinese: the JCEP study. Early Interv Psychiatry. 2013;9:118–125.

416. Lever-van Milligen BA, Vogelzangs N, Smit JH, Penninx BWJH. Hemoglobin levels in persons with depressive and/or anxiety disorders. J Psychosom Res. 2014;76:317–321.

417. Levinson D, Lerner Y. Employment among persons with past and current mood and anxiety disorders in the Israel National Health Survey. Psychiatr Serv. 2009;60:655—662.

418. Lewinsohn PM, Fenn DS, Stanton AK, Franklin J. Relation of age at onset to duration of episode in unipolar depression. Psychol Aging. 1986;1:63—68.

419. Lewinsohn PM, Rohde P, Seeley JR, Fischer SA. Age-Cohort Changes in the Lifetime Occurrence of Depression and Other Mental Disorders. J Abnorm Psychol. 1993;102:110–120.

420. Lieb R, Isensee B, Höfler M, Wittchen H-U. Parental depression and depression in offspring: evidence for familial characteristics and subtypes? J Psychiatr Res. 2002;36:237—246.

421. Locatelli L, Cadamuro M, Spirli C, Fiorotto R, Morell CM, Popov Y, et al. HHS Public Access.

Hepatology. 2017;63:965–982.

422. Löffler W, Häfner H, Riecher A, Maurer K, Strömgren E, Munk-Jørgensen P. How does gender influence age at first hospitalization for schizophrenia? A transnational case register study. Psychol Med. 2009;19:903.

423. Lyness JM, Pearson JL, Lebowitz BD, Kupfer DJ. Age at Onset or Late-Life Depression: A Research Agenda Report of a MacArthur Foundation-NIMH Workshop. Am J Geriatr Psychiatry. 1994;2:4.

424. Lyvers M, Duff H, Basch V, Edwards MS. Rash impulsiveness and reward sensitivity in relation to risky drinking by university students : Potential roles of frontal systems. Addict Behav. 2012;37:940–946.

425. Malmberg M, Overbeek G, Monshouwer K, Lammers J, Vollebergh WAM, Engels RCME. Substance use risk profiles and associations with early substance use in adolescence. J Behav Med. 2010;33:474—485.

426. Marcelis M, Navarro-Mateu F, Murray R, Selten JP, Van Os J. Urbanization and psychosis: a study of 1942-1978 birth cohorts in The Netherlands. Psychol Med. 1998;28:871—879.

427. Marmorstein NR. Associations between subtypes of major depressive episodes and substance use disorders. Psychiatry Res. 2011;186:248–253.

428. Matza LS, Revicki DA, Davidson JR, Stewart JW. Depression with atypical features in the National Comorbidity Survey: classification, description, and consequences. Arch Gen Psychiatry. 2003;60:817—826.

429. McCabe L, Cairney J, Veldhuizen S, Herrmann N, Streiner DL. Prevalence and correlates of agoraphobia in older adults. Am J Geriatr Psychiatry. 2006;14:515—522.

430. McCabe SE, Dickinson K, West BT, Wilens TE. Age of Onset, Duration, and Type of Medication Therapy for Attention-Deficit/Hyperactivity Disorder and Substance Use During Adolescence: A Multi-Cohort National Study. J Am Acad Child Adolesc Psychiatry. 2016;55:479–486.

431. McIntyre RS, Konarski JZ, Wilkins K, Bouffard B, Soczynska JK, Kennedy SH. The prevalence and impact of migraine headache in bipolar disorder: results from the Canadian Community Health Survey. Headache. 2006;46:973—982.

432. McKeganey N, McIntosh J, MacDonald F, Gilvarry E, McHardle P, McCarthy S, et al. Preteens and illegal drugs: Use, offers, exposure and prevention. Drugs. 2005. 2005.

433. McLaughlin KA, Green JG, Gruber MJ, Sampson NA, Zaslavsky AM, Kessler RC. Childhood adversities and adult psychiatric disorders in the national comorbidity survey replication II: associations with persistence of DSM-IV disorders. Arch Gen Psychiatry. 2010;67:124—132.

434. McLean CP, Asnaani A, Litz BT, Hofmann SG. Gender differences in anxiety disorders: Prevalence, course of illness, comorbidity and burden of illness. J Psychiatr Res. 2011;45:1027–1035.

435. Meier MH, Ph D, Caspi A, Ph D, Houts R, Ph D, et al. Prospective Developmental Subtypes of Alcohol Dependence from Age 18 to 32 Years: Implications for Nosology, Etiology, and Intervention Madeline. Dev Psychopathol Vol25(3), 2013, Pp 785-800. 2013;25:785–800.

436. Melchior M, Moffitt TE, Milne BJ, Poulton R, Caspi A. Why do children from socioeconomically disadvantaged families suffer from poor health when they reach adulthood? A life-course study. Am J Epidemiol. 2007;166:966–974.

437. Mikkelsen SS, Flensborg-Madsen T, Eliasen M, Mortensen EL. A longitudinal cohort study of intelligence and later hospitalisation with mental disorder. Compr Psychiatry. 2014;55:912–919.

438. Miller L, Gur M. Religiosity, depression, and physical maturation in adolescent girls. J Am Acad Child Adolesc Psychiatry. 2002;41:206—214.

439. Milne BJ, Caspi A, Harrington H, Poulton R, Rutter M, Moffitt TE. Predictive value of family history on severity of illness: the case for depression, anxiety, alcohol dependence, and drug dependence. Arch Gen Psychiatry. 2009;66:738—747.

440. P.L.H. M, C.B. P, D. S, A. A, N. K, S. A, et al. Parental psychiatric disease and risks of attempted suicide and violent criminal offending in offspring a population-based cohort study. JAMA Psychiatry. 2016;73:1015–1022.

441. Monshouwer K, Smit F, Ruiter M, Ormel H, Verhulst F, Vollebergh W, et al. Identifying target groups for the prevention of depression in early adolescence : The TRAILS study. J Affect Disord. 2012;138:287–294.

442. Montgomery S, Cook D, Bartley M, Wadsworth MEJ. Unemployment pre-dates symptoms of depression and anxiety resulting in medical consultation in young men. Int J Epidemiol. 1999;28:95–100.

443. Morean ME, Kong G, Camenga DR, Cavallo DA, Connell C. First Drink to First Drunk : Age of Onset and Delay to Intoxication Are Associated with Adolescent Alcohol Use and Binge Drinking. Alcohol Clin Exp Res Vol38(10), 2014, Pp 2615-2621. 2014;38:2615–2622.

444. Moss HB, Chen CM, Yi HY. Prospective follow-up of empirically derived alcohol dependence subtypes in wave 2 of the national epidemiologic survey on alcohol and related conditions (NESARC): Recovery status, alcohol use disorders and diagnostic criteria, alcohol consumption behavio. Alcohol Clin Exp Res. 2010;34:1073–1083.

445. Mota N, Alvarez-Gil R, Corral M, Rodríguez Holguín S, Parada M, Crego A, et al. Risky alcohol use and heavy episodic drinking among Spanish University students: a two-year follow-up. Gac Sanit. 2010;24:372—377.

446. Munkholm A, Olsen EM, Rask CU, Clemmensen L, Rimvall MK, Jeppesen P, et al. Early Predictors of Eating Problems in Preadolescence - A Prospective Birth Cohort Study. J Adolesc Heal. 2016;58:533–542.

447. Mustelin L, Raevuori A, Hoek HW, Kaprio J, Keski-Rahkonen A. Incidence and weight trajectories of binge eating disorder among young women in the community. Int J Eat Disord. 2015;48:1106–1112.

448. Mustonen A, Niemelä S, McGrath JJ, Murray GK, Nordström T, Mäki P, et al. Adolescent inhalant use and psychosis risk – a prospective longitudinal study. Schizophr Res. 2018;201:360–366.

449. Myles-Worsley M, Blailes F, Ord LM, Weaver S, Dever G, Faraone S V. The Palau Early Psychosis Study: Distribution of cases by level of genetic risk. Am J Med Genet Part B Neuropsychiatr Genet. 2007;144:5–9.

450. Neale MC, Eaves LJ, Hewitt JK, MacLean CJ, Meyer JM, Kendler KS. Analyzing the relationship between age at onset and risk to relatives. Am J Hum Genet. 1989;45:226—239.

451. Nesvåg R, Jönsson EG, Bakken IJ, Knudsen GP, Bjella TD, Reichborn-Kjennerud T, et al. The quality of severe mental disorder diagnoses in a national health registry as compared to research diagnoses based on structured interview. BMC Psychiatry. 2017;17:1–8.

452. Nettelbladt P, Göth M, Bogren M, Mattisson C. Risk of mental disorders in subjects with intellectual disability in the Lundby cohort 1947-97. Nord J Psychiatry. 2009;63:316–321.

453. Newman SC, Bland RC, Orn H. Morbidity Risk of Psychiatric Disorders. Acta Psychiatr Scand. 1988;77:50–56.

454. Nierenberg AA, Haley CL, Sung SC, Fava M, Hollon SD, Gaynes BN, et al. Is prior course of illness relevant to acute or longer-term outcomes in depressed out-patients? A STAR*D report. Psychol Med. 2011;42:1131–1149.

455. Nishiura C, Nanri A, Kashino I, Hori A, Kinugawa C, Endo M, et al. Age-, sex-, and diagnosis-specific incidence rate of medically certified long-term sick leave among private sector employees: The Japan Epidemiology Collaboration on Occupational Health (J-ECOH) study. J Epidemiol. 2017;27:590–595.

456. Nixon MK, Cloutier P, Jansson SM. Nonsuicidal self-harm in youth: a population-based survey. CMAJ. 2008;178:306—312.

457. Nock MK, Kazdin AE, Hiripi E, Kessler RC. Prevalence, subtypes, and correlates of DSM-IV conduct disorder in the National Comorbidity Survey Replication. Psychol Med. 2006;36:699–710.

458. Nock MK, Green JG, Hwang I, McLaughlin KA, Sampson NA, Zaslavsky AM, et al. Prevalence, correlates, and treatment of lifetime suicidal behavior among adolescents: Results from the national comorbidity survey replication adolescent supplement. JAMA Psychiatry. 2013;70:300–310.

459. Nolen WA, de Jonge P, Penninx BWJH, Roest AM, Schuch JJJ. Gender differences in major depressive disorder: Results from the Netherlands study of depression and anxiety. J Affect Disord. 2013;156:156–163.

460. Oakley Browne MA, Elisabeth Wells J, Mcgee MA. Twelve-Month and Lifetime Health Service use in Te Rau Hinengaro: The New Zealand Mental Health Survey. Aust New Zeal J Psychiatry. 2009;40:855–864.

461. Odgers CL, Caspi A, Broadbent JM, Dickson N, Hancox RJ, Harrington HL, et al. Prediction of differential adult health burden by conduct problem subtypes in males. Arch Gen Psychiatry. 2007;64:476–484.

462. Omer S, Waddington JL, O'Callaghan E, Pringle DG, Kirkbride JB, Russell V. Neighbourhood-level socio-environmental factors and incidence of first episode psychosis by place at onset in rural Ireland: The Cavan–Monaghan First Episode Psychosis Study [CAMFEPS]. Schizophr Res. 2013;152:152–157.

463. O'Neil A, Fisher AJ, Kibbey KJ, Jacka FN, Kotowicz MA, Williams LJ, et al. The addition of depression to the Framingham Risk Equation model for predicting coronary heart disease risk in women. Prev Med (Baltim). 2016;87:115—120.

464. Onyike CU, Johnston D, Rosenblatt A, Brandt J, Lyketsos CG, Samus QM, et al. 12-month incidence, prevalence, persistence, and treatment of mental disorders among individuals recently admitted to assisted living facilities in Maryland. Int Psychogeriatrics. 2013;25:721–731.

465. Osler M, Nybo Andersen AM, Nordentoft M. Impaired childhood development and suicidal behaviour in a cohort of Danish men born in 1953. J Epidemiol Community Health. 2008;62:23–28.

466. Paananen R, Ristikari T, Merikukka M, Gissler M. Social determinants of mental health: A finnish nationwide follow-up study on mental disorders. J Epidemiol Community Health. 2013;67:1025–1031.

467. Pacheco A, Barguil M, Contreras J, Montero P, Dassori A, Escamilla MA, et al. Social and clinical comparison between schizophrenia and bipolar disorder type I with psychosis in Costa Rica. Soc Psychiatry Psychiatr Epidemiol. 2010;45:675–680.

468. Pardo G, Manrique JFD, Vázquez-Barquero JL, Dunn G, Castanedo SH, Nuñez MJC. Sociodemographic and clinical variables as predictors of the diagnostic characteristics of first episodes of schizophrenia. Acta Psychiatr Scand. 2007;94:149–155.

469. Parker G, Hadzi-Pavlovic D. Is the female preponderance in major depression secondary to a gender difference in specific anxiety disorders? Psychol Med. 2004;34:461—470.

470. Pasco J, Henry M, Berk M, Nicholson G, Kotowicz M, Dodd S, et al. The Prevalence of Mood and Anxiety Disorders in Australian Women. Australas Psychiatry. 2010;18:250–255.

471. Paykel ES, Watters L, Abbott R, Wadsworth M. Do treated psychiatric patients become later community cases?. A prospective cohort study. Eur Psychiatry. 2006;21:315–318.

472. Pedersen CG, Jensen SOW, Johnsen SP, Nordentoft M, Mainz J. Processes of in-hospital psychiatric care and subsequent criminal behaviour among patients with schizophrenia: A National population-based, follow-up study. Can J Psychiatry. 2013;58:515–521.

473. Perroud N, Uher R, Hauser J, Rietschel M, Henigsberg N, Placentino A, et al. History of suicide attempts among patients with depression in the GENDEP project. J Affect Disord. 2010;123:131–137.

474. Pfister H, Wittchen HU, Hoyer J, Muller AG, Hofler M, Lieb R, et al. Physical activity and prevalence and incidence of mental disorders in adolescents and young adults. Psychol Med. 2007;37:1657–1666.

475. Pilatti A, Caneto F, Garimaldi JA, Vera BDV, Pautassi RM. Contribution of time of drinking onset and family history of alcohol problems in alcohol and drug use behaviors in Argentinean college students. Alcohol Alcohol. 2014;49:128—137.

476. Pitkänen T, Lyyra A-L, Pulkkinen L. Age of onset of drinking and the use of alcohol in adulthood: a follow-up study from age 8-42 for females and males. Addiction. 2005;100:652—661.

477. Pitkänen T, Kokko K, Lyyra AL, Pulkkinen L. A developmental approach to alcohol drinking behaviour in adulthood: A follow-up study from age 8 to age 42. Addiction. 2008;103:48–68.

478. Platt JM, Keyes KM, McLaughlin KA, Kaufman AS. Intellectual disability and mental disorders in a US population representative sample of adolescents. Psychol Med. 2019;49:952—961.

479. Polanczyk G, Caspi A, Houts R, Kollins SH, Rohde LA, Moffitt TE. Implications of extending the ADHD age-of-onset criterion to age 12: results from a prospectively studied birth cohort. J Am Acad Child Adolesc Psychiatry. 2010;49:210—216.

480. Pope HG, Kanayama G, Athey A, Ryan E, Hudson JI, Baggish A. The lifetime prevalence of anabolic-androgenic steroid use and dependence in Americans: current best estimates. Am J Addict. 2014;23:371—377.

481. Posada J, Camacho J, Valenzuela J, Arguello A, Cendales J, Fajardo R. Prevalence of suicide risk factors and suicide-related outcomes in the National Mental Health Study, Colombia. Suicide Life-Threatening Behav. 2009;39:408–424.

482. Power RA, Kyaga S, Uher R, MacCabe JH, Långström N, Landen M, et al. Fecundity of patients with schizophrenia, autism, bipolar disorder, depression, anorexia nervosa, or substance abuse vs their unaffected siblings. Arch Gen Psychiatry. 2013;70:22–30.

483. Prelipceanu D, Elston RC, Grimberg M, Sima D, Mihailescu R, Grigoroiu-Serbanescu M, et al. Paternal age effect on age of onset in bipolar I disorder is mediated by sex and family history. Am J Med Genet Part B Neuropsychiatr Genet. 2012;159B:567–579.

484. Rabinowitz J, Levine SZ, Häfner H. A population based elaboration of the role of age of onset on the course of schizophrenia. Schizophr Res. 2006;88:96–101.

485. M.-S. R, C.L.-W. C, M.-Z. X, Q.-H. W. Suicide attempts among patients with psychosis in a Chinese rural community. Acta Psychiatr Scand. 2003;107:430–435.

486. Räsänen P, Tiihonen J, Hakko H. The incidence and onset-age of hospitalized bipolar affective disorder in Finland. J Affect Disord. 1998;48:63—68.

487. Ratnasingham S, Cairney J, Manson H, Rehm J, Lin E, Kurdyak P. The burden of mental illness and addiction in Ontario. Can J Psychiatry. 2013;58:529–537.

488. Razavi T, Clark C, Stansfeld SA. Work-family conflict as a predictor of common mental disorders in the 1958 British birth cohort. Longit Life Course Stud. 2015;6:264–278.

489. Reichenberg A, Weiser M, Caspi A, Knobler HY, Lubin G, Harvey PD, et al. Premorbid intellectual functioning and risk of schizophrenia and spectrum disorders. J Clin Exp Neuropsychol. 2006;28:193—207.

490. Resnick MD, Bearman PS, Blum RW, Bauman KE, Harris KM, Jones J, et al. Protecting adolescents from harm. Findings from the National Longitudinal Study on Adolescent Health. JAMA. 1997;278:823—832.

491. Reuter M, Hennig J, Amelang M, Montag C, Korkut T, Hueweler A, et al. The role of the TPH1 and TPH2 genes for nicotine dependence: a genetic association study in two different age cohorts. Neuropsychobiology. 2007;56:47—54.

492. Ridenour TA, Kirisci L, Tarter RE, Vanyukov MM. Could a continuous measure of individual transmissible risk be useful in clinical assessment of substance use disorder ? Findings from the National Epidemiological Survey on Alcohol and Related Conditions ಔ. Drug Alcohol Depend. 2011;119:10–17.

493. Roberts ME, Fuemmeler BF, McClernon FJ, Beckham JC. Association Between Trauma Exposure and Smoking in a Population-Based Sample of Young Adults. J Adolesc Heal. 2008. 2008. https://doi.org/10.1016/j.jadohealth.2007.08.029.

494. Rössler W, Hengartner MP, Ajdacic-Gross V, Haker H, Angst J. Sex differences in sub-clinical psychosis--results from a community study over 30 years. Schizophr Res. 2012;139:176—182.

495. Rubio JM, Olfson M, Pérez-Fuentes G, Garcia-Toro M, Wang S, Blanco C. Effect of first episode Axis I disorders on quality of life. J Nerv Ment Dis. 2014;202:271–274.

496. Sanchez ZM, Santos MGR, Pereira APD, Nappo SA, Carlini EA, Carlini CM, et al. Childhood Alcohol Use May Predict Adolescent Binge Drinking: A Multivariate Analysis among Adolescents in Brazil. J Pediatr. 2013;163:363–368.

497. Savolainen K, Räikkönen K, Kananen L, Kajantie E, Hovatta I, Lahti M, et al. History of mental disorders and leukocyte telomere length in late adulthood: The Helsinki Birth Cohort Study (HBCS). J Psychiatr Res. 2012;46:1346–1353.

498. Schneider C, Cerwenka S, Nieder TO, Briken P, Cohen-Kettenis PT, De Cuypere G, et al. Measuring Gender Dysphoria: A Multicenter Examination and Comparison of the Utrecht Gender Dysphoria Scale and the Gender Identity/Gender Dysphoria Questionnaire for Adolescents and Adults. Arch Sex Behav. 2016;45:551–558.

499. Schoeyen HK, Vaaler AE, Auestad BH, Malt UF, Melle I, Andreassen OA, et al. Despite clinical differences, bipolar disorder patients from acute wards and outpatient clinics have similar educational and disability levels compared to the general population. J Affect Disord. 2011;132:209–215.

500. Scocco P, de Girolamo G, Vilagut G, Alonso J. Prevalence of suicide ideation, plans, and attempts and related risk factors in Italy: results from the European Study on the Epidemiology of Mental Disorders--World Mental Health study. Compr Psychiatry. 2008;49:13—21.

501. Scott KM, Korff M Von, Alonso J, Angermeyer MC, Benjet C, Bruffaerts R, et al. Childhood adversity, early-onset depressive/anxiety disorders, and adult-onset asthma. Psychosom Med. 2008;70:1035–1043.

502. Scott KM, Von Korff M, Angermeyer MC, Benjet C, Bruffaerts R, de Girolamo G, et al. Association of childhood adversities and early-onset mental disorders with adult-onset chronic physical conditions. Arch Gen Psychiatry. 2011;68:838—844.

503. Scott KM, Alonso J, de Jonge P, Viana MC, Liu Z, O’Neill S, et al. Associations between DSM-IV mental disorders and onset of self-reported peptic ulcer in the World Mental Health Surveys. J Psychosom Res. 2013;75:121—127.

504. Seedat S, Scott KM, Angermeyer MC, Berglund P, Bromet EJ, Brugha TS, et al. Cross-national

associations between gender and mental disorders in the World Health Organization World Mental Health Surveys. Arch Gen Psychiatry. 2009;66:785—795.

505. Shaffer D, Fisher P, Dulcan MK, Davies M, Piacentini J, Schwab-Stone ME, et al. The NIMH Diagnostic Interview Schedule for Children Version 2.3 (DISC- 2.3): Description, acceptability, prevalence rates, and performance in the MECA study. J Am Acad Child Adolesc Psychiatry. 1996;35:865–877.

506. Shang LL, Huang YQ, Liu ZR, Chen HG. A cross-sectional survey of disability attributed to mental disorders and service use in China. Chin Med J (Engl). 2017;130:1441–1445.

507. Shillington AM, Clapp JD. Self-report stability of adolescent substance use: are there differences for gender, ethnicity and age? Drug Alcohol Depend. 2000;60:19—27.

508. Shillington AM, Roesch SC, Reed MB, Clapp JD, Woodruff SI. Typologies of recanting of lifetime cigarette , alcohol and marijuana use during a six-year longitudinal panel study. Drug Alcohol Depend. 2011;118:134–140.

509. Silberg J, Moore AA, Rutter M. Age of onset and the subclassification of conduct/dissocial disorder. J Child Psychol Psychiatry Allied Discip. 2015;56:826–833.

510. Silver E, Arseneault L, Langley J, Caspi A, Moffitt TE. Mental disorder and violent victimization in a total birth cohort. Am J Public Health. 2005;95:2015–2021.

511. Simon GE, Vonkorff M. Reevaluation of Secular Trends in Depression Rates. Am J Epidemiol 135(12)1411-22, 1992 Jun 15. 1992;135:1411–1422.

512. Skodol AE, Grilo CM, Keyes KM, Geier T, Grant BF, Hasin DS. Relationship of personality disorders to the course of major depressive disorder in a nationally representative sample. Am J Psychiatry. 2011. 2011. https://doi.org/10.1176/appi.ajp.2010.10050695.

513. Skodol AE, Geier T, Grant BF, Hasin DS. Personality disorders and the persistence of anxiety disorders in a nationally representative sample. Depress Anxiety. 2014;31:721—728.

514. Smart D, Hayes A, Sanson A, Toumbourou JW. Mental health and wellbeing of Australian adolescents: pathways to vulnerability and resilience. Int J Adolesc Med Health. 2007;19:263—268.

515. Smyth BP, Kelly A, Cox G. Decline in age of drinking onset in Ireland, gender and per capita alcohol consumption. Alcohol Alcohol. 2011. 2011. https://doi.org/10.1093/alcalc/agr047.

516. Sørensen HJ, Andersen SB, Karstoft KI, Madsen T. The influence of pre-deployment cognitive ability on post-traumatic stress disorder symptoms and trajectories: The Danish USPER follow-up study of Afghanistan veterans. J Affect Disord. 2016;196:148–153.

517. Sourander A, Jensen P, Davies M, Niemelä S, Elonheimo H, Ristkari T, et al. Who is at greatest risk of adverse long-term outcomes? The Finnish from a boy to a man study. J Am Acad Child Adolesc Psychiatry. 2007;46:1148–1161.

518. Spaner D, Bland RC, Newman SC. Epidemiology of psychiatric disorders in Edmonton. Major depressive disorder. Acta Psychiatr Scand Suppl. 1994;376:7—15.

519. Spauwen J, Krabbendam L, Lieb R, Wittchen HU, van Os J. Sex differences in psychosis: normal or pathological? Schizophr Res. 2003;62:45—49.

520. Spiers N, Bebbington P, McManus S, Brugha TS, Jenkins R, Meltzer H. Age and birth cohort differences in the prevalence of common mental disorder in England: National psychiatric morbidity surveys 1993-2007. Br J Psychiatry. 2011;198:479–484.

521. Stein MB, Stein DJ. Social anxiety disorder. 2008:1115–1125.

522. Stein DJ, Ruscio AM, Lee S, Petukhova M, Alonso J, Andrade LHSG, et al. Subtyping social anxiety disorder in developed and developing countries. Depress Anxiety. 2010;27:390–403.

523. Stevens H, Dean K, Agerbo E, Nielsen PR, Mortensen PB, Nordentoft M. Offending prior to first psychiatric contact: a population-based register study. Psychol Med. 2012;42:2673–2684.

524. Stice E, Killen JD, Hayward C, Taylor CB. Age of onset for binge eating and purging during late adolescence: a 4-year survival analysis. J Abnorm Psychol. 1998;107:671—675.

525. Strunin L, Edwards EM, Godette DC, Heeren T. Country of origin, age of drinking onset, and drinking patterns among Mexican American young adults. Drug Alcohol Depend. 2007;91:134—140.

526. Strunin L, Díaz Martínez A, Díaz-Martínez LR, Heeren T, Kuranz S, Winter M, et al. Parental monitoring and alcohol use among Mexican students. Addict Behav. 2013;38:2601—2606.

527. Swartz MS, Landerman R, George LK, Turnbull JE, Blazer DG. Social outcomes related to age of onset among psychiatric disorders. J Consult Clin Psychol. 2005;58:832–839.

528. Tanskanen P, Jaaskelainen E, Isohanni MK, Ridler K, Bullmore ET, Miettunen J, et al. Morphometric Brain Abnormalities in Schizophrenia in a Population-Based Sample: Relationship to Duration of Illness. Schizophr Bull. 2008;36:766–777.

529. Tantoh DM, Lee J-F, Chiang Y-C, Lee C-T, Huang J-Y, Liaw Y-P, et al. Incidence of Major Depressive Disorder. Medicine (Baltimore). 2016;95:e3110.

530. Tebeka S, Le Strat Y, Dubertret C. Is parity status associated with bipolar disorder clinical features, severity or evolution? J Affect Disord. 2018;225:201–206.

531. Tegethoff M, Stalujanis E, Belardi A, Meinlschmidt G. Chronology of onset of mental disorders and physical diseases in mental-physical comorbidity - A national representative survey of adolescents. PLoS One. 2016;11:1–19.

532. Ten Have M, Vollebergh W, Bijl R, Ormel J. Combined effect of mental disorder and low social support on care service use for mental health problems in the Dutch general population. Psychol Med. 2002;32:311–323.

533. Ten Have M, Vollebergh W, Bijl R, Nolen WA. Bipolar disorder in the general population in The Netherlands (prevalence, consequences and care utilisation): Results from The Netherlands Mental Health Survey and Incidence Study (NEMESIS). J Affect Disord. 2002;68:203–213.

534. ten Have M, de Graaf R, van Dorsselaer S, Beekman A. Lifetime Treatment Contact and Delay in Treatment Seeking After First Onset of a Mental Disorder. Psychiatr Serv. 2013;64:981–989.

535. Ten Have M, Nuyen J, Beekman A, De Graaf R. Common mental disorder severity and its association with treatment contact and treatment intensity for mental health problems. Psychol Med. 2013;43:2203–2213.

536. Ten Have M, Van Dorsselaer S, De Graaf R. Prevalence and risk factors for first onset of suicidal behaviors in the Netherlands Mental Health Survey and Incidence Study-2. J Affect Disord. 2013;147:205–211.

537. Thapar A, Hammerton G, Harold G, Thapar A. Depression and blood pressure in high-risk children and adolescents: An investigation using two longitudinal cohorts. BMJ Open. 2013;3:1–9.

538. Thomas G, Reifman A, Barnes G, Farrell M. Delayed onset of drunkenness as a protective factor for adolescent alcohol misuse and sexual risk taking: a longitudinal study. Deviant Behav. 2000;21:181–209.

539. Thompson AH, Dewa CS, Phare S. The suicidal process: age of onset and severity of suicidal behaviour. Soc Psychiatry Psychiatr Epidemiol. 2012;47:1263—1269.

540. Taka-Eilola (nèe Riekki) T, Veijola J, Murray GK, Koskela J, Mäki P. Severe mood disorders and schizophrenia in the adult offspring of antenatally depressed mothers in the Northern Finland 1966 Birth Cohort: Relationship to parental severe mental disorder. J Affect Disord. 2019;249:63–72.

541. Trinkoff AM, Anthony JC, Muñoz A. Predictors of the initiation of psychotherapeutic medicine use. Am J Public Health. 1990;80:61—65.

542. Trumpf J, Margraf J, Vriends N, Meyer AH, Becker ES. Specific phobia predicts psychopathology in young women. Soc Psychiatry Psychiatr Epidemiol. 2010;45:1161—1166.

543. Udo T, Bitley S, Grilo CM. Suicide attempts in US adults with lifetime DSM-5 eating disorders. BMC Med. 2019;17:120.

544. Vaillant GE. Evidence that the type 1/type 2 dichotomy in alcoholism must be re-examined. Addiction. 1994;89:1049—57; discussion 1059—70.

545. van Balkom AJLM, Comijs HC, van Oppen P, van der Steenstraten IM, Stek ML, Rhebergen D, et al. Admixture analysis of age of onset in generalized anxiety disorder. J Anxiety Disord. 2017;50:47–51.

546. Beek JHDA Van, Willemsen G, Moor MHM De, Hottenga JJ, Boomsma DI. Associations Between ADH Gene Variants and Alcohol Phenotypes in Dutch Adults. Twin Res Hum Genet Vol13(1), 2010, Pp 30-42. 2010;13:30–42.

547. van Lang NDJ, Ferdinand RF, Verhulst FC. Predictors of future depression in early and late adolescence. J Affect Disord. 2007;97:137—144.

548. VAN OS J, MURRAY R, SELTEN J-P, NAVARRO-MATEU F, MARCELIS M. Urbanization and psychosis: a study of 1942–1978 birth cohorts in The Netherlands. Psychol Med. 2002;28:871–879.

549. van Ours JC. Dynamics in the use of drugs. Health Econ. 2006;15:1283—1294.

550. Vega WA, Aguilar-Gaxiola S, Andrade L, Bijl R, Borges G, Caraveo-Anduaga JJ, et al. Prevalence and age of onset for drug use in seven international sites: results from the international consortium of psychiatric epidemiology. Drug Alcohol Depend. 2002;68:285—297.

551. Verhagen M, Van Der Meij A, Franke B, Vollebergh W, De Graaf R, Buitelaar J, et al. Familiality of major depressive disorder and gender differences in comorbidity. Acta Psychiatr Scand. 2008;118:130–138.

552. Verster JC, van Herwijnen J, Olivier B, Kahler CW. Validation of the Dutch version of the brief young adult alcohol consequences questionnaire (B-YAACQ). Addict Behav. 2009;34:411—414.

553. Viana MC, Lim CCW, Garcia Pereira F, Aguilar-Gaxiola S, Alonso J, Bruffaerts R, et al. Previous Mental Disorders and Subsequent Onset of Chronic Back or Neck Pain: Findings From 19 Countries. J Pain. 2018;19:99–110.

554. Vilalta-Franch J, López-Pousa S, Llinàs-Reglà J, Calvó-Perxas L, Merino-Aguado J, Garre-Olmo J. Depression subtypes and 5-year risk of dementia and Alzheimer disease in patients aged 70 years. Int J Geriatr Psychiatry. 2013;28:341—350.

555. Vilsaint CL, NeMoyer A, Fillbrunn M, Sadikova E, Kessler RC, Sampson NA, et al. Racial/ethnic differences in 12-month prevalence and persistence of mood, anxiety, and substance use disorders: Variation by nativity and socioeconomic status. Compr Psychiatry. 2019;89:52—60.

556. Vitola ES, Bau CHD, Salum GA, Horta BL, Quevedo L, Barros FC, et al. Exploring DSM-5 ADHD criteria beyond young adulthood: Phenomenology, psychometric properties and prevalence in a large three-decade birth cohort. Psychol Med. 2017;47:744–754.

557. Vitousek K, Manke F. Personality Variables and Disorders in Anorexia Nervosa and Bulimia Nervosa. J Abnorm Psychol. 1994.

558. Korff V, Eaton WW, Coop- GH, Hopkins J, Hopkins J, Health G, et al. The epidemiology of panic attacks and panic disorder. Results of three community surveys. Am J Epidemiol 122(6)970-81, 1985 Dec. 1985;122:970–981.

559. Walker NP, McConville PM, Hunter D, Deary IJ, Whalley LJ. Childhood mental ability and lifetime

psychiatric contact. Intelligence. 2002;30:233–245.

560. Wang PS, Berglund P, Olfson M, Pincus HA, Wells KB, Kessler RC. Failure and delay in initial treatment contact after first onset of mental disorders in the National Comorbidity Survey Replication. Arch Gen Psychiatry. 2005.

561. Wang H, Lin SL, Leung GM, Schooling CM. Age at Onset of Puberty and Adolescent Depression: 'Children of 1997' Birth Cohort. Pediatrics. 2016;137.

562. Warner LA, White HR, Johnson V. Alcohol initiation experiences and family history of alcoholism as predictors of problem-drinking trajectories. J Stud Alcohol Drugs. 2007;68:56—65.

563. Weitzman ER, Chen Y-Y. The co-occurrence of smoking and drinking among young adults in college: national survey results from the United States. Drug Alcohol Depend. 2005;80:377—386.

564. Wells JE, McGee MA. Violations of the usual sequence of drug initiation: prevalence and associations with the development of dependence in the New Zealand Mental Health Survey. J Stud Alcohol Drugs. 2008;69:789—795.

565. Wells JE, McGee MA, Baxter J, Agnew F, Kokaua J, New Zealand Mental Health Survey Research Team. Onset and lifetime use of drugs in New Zealand: results from Te Rau Hinengaro: the New Zealand Mental Health Survey 2003-2004. Drug Alcohol Rev. 2009;28:166—174.

566. Wessely SC, Castle D, Douglas AJ, Taylor PJ. The criminal careers of incident cases of schizophrenia. Psychol Med. 1994;24:483—502.

567. Willoughby MT, Kollins SH, Mcclernon FJ, Family T, Investigative L. Association between smoking and retrospectively reported attention-defi cit / hyperactivity disorder symptoms in a large sample of new mothers. 2009;11:313–322.

568. Wilson DM, Killen JD, Hayward C, Robinson TN, Hammer LD, Kraemer HC, et al. Timing and rate of sexual maturation and the onset of cigarette and alcohol use among teenage girls. Arch Pediatr Adolesc Med. 1994;148:789—795.

569. Wilson S, Vaidyanathan U, Miller MB, McGue M, Iacono WG. Premorbid risk factors for major depressive disorder: Are they associated with early onset and recurrent course? Dev Psychopathol. 2014;26:1477–1493.

570. Wisniewski K. Delinquency, Academic Underachievement, and Attention Deficit Hyperactivity Disorder: A Longitudinal Investigation of Developmental Sequencing and Interrelated Risk Factors. (Doctoral Diss Duquesne Univ Retrieved from Https//DscDuqEdu/Etd/1374. 2006. 2006.

571. Wittchen HU, Essau CA, von Zerssen D, Krieg JC, Zaudig M. Lifetime and six-month prevalence of mental disorders in the Munich Follow-Up Study. Eur Arch Psychiatry Clin Neurosci. 1992;241:247—258.

572. Wittchen H-U, Nocon A, Beesdo K, Pine DS, Hofler M, Lieb R, et al. Agoraphobia and panic. Prospective-longitudinal relations suggest a rethinking of diagnostic concepts. Psychother Psychosom. 2008;77:147—157.

573. Wright F, Bewick BM, Barkham M, House AO, Hill AJ. Co-occurrence of self-reported disordered eating and self-harm in UK university students. Br J Clin Psychol. 2009. 2009. https://doi.org/10.1348/014466509X410343.

574. Wrobel A. The impact of economic restructuring on the spatial pattern of urbanization in Poland. Landsc Urban Plan. 1992;22:115–120.

575. "Wu SS, Mittal V, Pennington B, Willcutt EG. Mathematics achievement scores and early psychosis in school-aged children. Schizophr Res. 2014;156:133–134.

576. Yates WR, Meller WH, Lund BC, Thurber S, Grambsch PL. Early-onset major depressive disorder in men is associated with childlessness. J Affect Disord. 2010;124:187–190.

577. YUCUN S, SHURAN L, CHANGHUI C, WEIXI Z. An epidemiological survey on neuroses of urban elderly in Beijing. Psychiatry Clin Neurosci. 1998;52:S288–S290.

578. Zakrajsek JS, Shope JT. Longitudinal examination of underage drinking and subsequent drinking and risky driving. J Safety Res. 2006;37:443—451.

579. Abbott DW, Zwaan M De, Mussell MP, Raymond NC, Seim HC, Crow SJ, et al. ONSET OF BINGE EATING AND DIETING IN OVERWEIGHT WOMEN : IMPLICATIONS FOR ETIOLOGY , ASSOCIATED FEATURES AND TREATMENT. J Psychosom Res 44(3-4)367-74, 1998 Mar-Apr. 1998;44:367–374.

580. Agrawal A, Madden PAF, Bucholz KK, Heath AC, Lynskey MT. Initial reactions to tobacco and cannabis smoking : a twin study. Addict Vol109(4), 2014, Pp 663-671. 2014:663–671.

581. Akvardar Y, Demiral Y, Ergor G, Ergor A. Substance use among medical students and physicians in a medical school in Turkey. Soc Psychiatry Psychiatr Epidemiol. 2004;39:502—506.

582. Alda M, Ahrens B, Lit W, Dvorakova M, Labelle A, Zvolsky P, et al. Age of onset in familial and sporadic schizophrenia. Acta Psychiatr Scand. 1996;93:447–450.

583. Allegri F, Belvederi Murri M, Paparelli A, Marcacci T, Braca M, Menchetti M, et al. Current cannabis use and age of psychosis onset: A gender-mediated relationship? Results from an 8-year FEP incidence study in Bologna. Psychiatry Res. 2013;210:368–370.

584. Almeida OP, Fenner S. Bipolar disorder: Similarities and differences between patients with illness onset before and after 65 years of age. Int Psychogeriatrics. 2002;14:311–322.

585. Anand A, Koller DL, Lawson WB, Gershon ES, Nurnberger JI. Genetic and childhood trauma interaction effect on age of onset in bipolar disorder: An exploratory analysis. J Affect Disord. 2015;179:1–5.

586. Arunpongpaisal S, Sangsirilak A. Using MoCA-Thai to evaluate cognitive impairment in patients with schizophrenia. J Med Assoc Thai. 2013;96:860–865.

587. Baldessarini RJ, Bolzani L, Cruz N, Jones PB, Lai M, Lepri B, et al. Onset-age of bipolar disorders at six international sites. J Affect Disord. 2010;121:143–146.

588. Bar JL. A twin study of the relationship between behavioral disinhibition and alcoholism. 2003.

589. Barnes ER. Psychotic-like experiences and age at first use of cannabis in a non-clinical sample. 2011.

590. Bauer M, Glenn T, Alda M, Andreassen OA, Ardau R, Bellivier F, et al. Impact of sunlight on the age of onset of bipolar disorder. Bipolar Disord. 2012;14:654–663.

591. Bauer M, Glenn T, Alda M, Andreassen OA, Angelopoulos E, Ardau R, et al. Influence of birth cohort on age of onset cluster analysis in bipolar I disorder. Eur Psychiatry. 2015;30:99–105.

592. Beekman AT, Deeg DJ, Smit JH, Comijs HC, Braam AW, de Beurs E, et al. Dysthymia in later life: a study in the community. J Affect Disord. 2004;81:191—199.

593. Benazzi F. A tetrachoric factor analysis validation of mixed depression. Prog Neuro-Psychopharmacology Biol Psychiatry. 2008;32:186–192.

594. Borga P, Widerlov B, Cullberg J, Patterns SC. Patterns of care among people with long-term functional psychosis in three different areas of Stockholm County. Acta Psychiatr Scand 83(3)223-33, 1991 Mar. 1991. 1991.

595. Borgå P, Widerlöv B, Stefansson CG, Cullberg J. Social conditions in a total population with long-term functional psychosis in three different areas of Stockholm County. Acta Psychiatr Scand. 1992;85:465–473.

596. Bourne C, Bilderbeck A, Drennan R, Atkinson L, Price J, Geddes JR, et al. Verbal learning

impairment in euthymic bipolar disorder: BDI v BDII. J Affect Disord. 2015;182:95–100.

597. Brown C, Miguel EC, Lochner C, Shavitt RG, Torres AR, Shyam Sundar A, et al. Comorbidity, age of onset and suicidality in obsessive–compulsive disorder (OCD): An international collaboration. Compr Psychiatry. 2017;76:79–86.

598. Bueno B, Krug I, Bulik CM, Jiménez-Murcia S, Granero R, Thornton L, et al. Late onset eating disorders in Spain: Clinical characteristics and therapeutic implications. J Clin Psychol. 2014;70:1–17.

599. Bulayeva KB, Leal SM, Pavlova TA, Kurbanov R, Coover S, Bulayev O, et al. The ascertainment of multiplex schizophrenia pedigrees from Daghestan genetic isolates (Northern Caucasus, Russia). Psychiatr Genet. 2000;10:67–72.

600. Buoli M, Bertino V, Caldiroli A, Dobrea C, Serati M, Ciappolino V, et al. Are obstetrical complications really involved in the etiology and course of schizophrenia and mood disorders? Psychiatry Res. 2016;241:297–301.

601. Bureau A, Chagnon YC, Croteau J, Fournier A, Roy MA, Paccalet T, et al. Follow-up of a major psychosis linkage site in 13q13-q14 reveals significant association in both case-control and family samples. Biol Psychiatry. 2013;74:444–450.

602. Butwicka A, Gmitrowicz A. Symptom clusters in obsessive-compulsive disorder (OCD): Influence of age and age of onset. Eur Child Adolesc Psychiatry. 2010;19:365–370.

603. Caamaño-Isorna F, Corral M, Parada M, Cadaveira F. Factors associated with risky consumption and heavy episodic drinking among Spanish university students. J Stud Alcohol Drugs. 2008;69:308—312.

604. D P, Cadenhead KS, Calkins ME, Dobie DJ, Freedman R, Greenwood TA, et al. the Consortium on the Genetics of Schizophrenia. Schizophrenia. 2009;103:218–228.

605. Capella M del M, Benaiges I, Adan A. Neuropsychological Performance in Polyconsumer Men Under Treatment. Influence of Age of Onset of Substance Use. Sci Rep. 2015;5:12038.

606. Carter JC, Bewell-Weiss C V. Nonfat phobic anorexia nervosa: Clinical characteristics and response to inpatient treatment. Int J Eat Disord. 2011;44:220–224.

607. Castle DJ, Wessely S, Murray RM. Sex and Schizophrenia : and Associations Effects of Diagnostic Stringency , with Premorbid Variables. Br J Psychiatry 162658-64, 1993 May. 1993:658–665.

608. Chen J, Cai Y, Cong E, Liu Y, Gao J, Li Y, et al. Childhood sexual abuse and the development of recurrent major depression in Chinese women. PLoS One. 2014;9:e87569.

609. Chen L, Selvendra A, Stewart A, Castle D. Risk factors in early and late onset schizophrenia. Compr Psychiatry. 2018;80:155–162.

610. Chengappa KNR, Kupfer DJ, Frank E, Houck PR, Grochocinski VJ, Cluss PA, et al. Relationship of birth cohort and early age at onset of illness in a bipolar disorder case registry. Am J Psychiatry. 2003;160:1636—1642.

611. Chou K-L, Cheung KC-K. Major depressive disorder in vulnerable groups of older adults, their course and treatment, and psychiatric comorbidity. Depress Anxiety. 2013;30:528—537.

612. Cieslak K, Pato M, Buckley P, Pato C, Sobell JL, Medeiros H, et al. Traumatic brain injury and bipolar psychosis in the Genomic Psychiatry Cohort. Am J Med Genet B Neuropsychiatr Genet. 2016;171:506–512.

613. Col SE, Caykoylu A, Karakas Ugurlu G, Ugurlu M. Factors affecting treatment compliance in patients with bipolar I disorder during prophylaxis: A study from Turkey. Gen Hosp Psychiatry. 2014;36:208–213.

614. Cong E, Li Y, Shao C, Chen J, Wu W, Shang X, et al. Childhood sexual abuse and the risk for recurrent major depression in Chinese women. Psychol Med. 2012;42:409–417.

615. Corruble E, Gorwood P, Falissard B. Association between age of onset and symptom profiles of late-life depression. Acta Psychiatr Scand. 2008;118:389–394.

616. Coryell W, Fiedorowicz J, Leon AC, Endicott J, Keller MB. Age of onset and the prospectively observed course of illness in bipolar disorder. J Affect Disord. 2013;146:34–38.

617. Cotton SM, Lambert M, Schimmelmann BG, Mackinnon A, Gleeson JFM, Berk M, et al. Differences between first episode schizophrenia and schizoaffective disorder. Schizophr Res. 2013;147:169–174.

618. Cuttler C, Spradlin A. Measuring cannabis consumption: Psychometric properties of the Daily Sessions, Frequency, Age of Onset, and Quantity of Cannabis Use Inventory (DFAQ-CU). PLoS One. 2017;12:1–14.

619. Cuypere G, Kreukels BPC, Richter-Appelt H, Cohen-Kettenis PT, Nieder TO, Haraldsen IRH, et al. Body Image in Young Gender Dysphoric Adults: A European Multi-Center Study. Arch Sex Behav. 2015;45:559–574.

620. Dakanalis A, Pla-Sanjuanelo J, Caslini M, Volpato C, Riva G, Clerici M, et al. Predicting onset and maintenance of men's eating disorders. Int J Clin Health Psychol. 2016;16:247—255.

621. Das P, Sreedaran P, MV A. A study to compare the differences between genders in psychiatric comorbidities in individuals with psychoses. Asian J Psychiatr. 2018;32:84–88.

622. De la Torre R, Sancho C, Casanueva FF, Frühbeck G, Menchón JM, Treasure J, et al. Lifetime Obesity in Patients with Eating Disorders: Increasing Prevalence, Clinical and Personality Correlates. Eur Eat Disord Rev. 2012;20:250–254.

623. del Pino-Gutiérrez A, Islam MA, Granero R, Gómez-Peña M, Savvidou LG, Fernández-Aranda F, et al. Comparative analysis of distinct phenotypes in gambling disorder based on gambling preferences. BMC Psychiatry. 2015;15:1–11.

624. Dell'Osso B, Benatti B, Buoli M, Altamura AC, Marazziti D, Hollander E, et al. The influence of age at onset and duration of illness on long-term outcome in patients with obsessive-compulsive disorder: A report from the International College of Obsessive Compulsive Spectrum Disorders (ICOCS). Eur Neuropsychopharmacol. 2013;23:865–871.

625. Delucchi KL, Katerberg H, Stewart SE, Denys DAJP, Lochner C, Stack DE, et al. Latent class analysis of the Yale-Brown Obsessive-Compulsive Scale symptoms in obsessive-compulsive disorder. Compr Psychiatry. 2011;52:334–341.

626. Dierkhising CB, Ko SJ, Woods-jaeger B, Briggs EC, Lee R, Pynoos RS. Trauma histories among justice-involved youth: findings from the National Child Traumatic Stress Network. Eur J Psychotraumatology Vol4 2013, ArtID 20274. 2013;1:1–12.

627. Docherty AR, Edwards AC, Yang F, Peterson RE, Sawyers C, Adkins DE, et al. Age of onset and family history as indicators of polygenic risk for major depression. Depress Anxiety. 2017;34:446–452.

628. Dominguez M de G, Fisher HL, Major B, Chisholm B, Rahaman N, Joyce J, et al. Duration of untreated psychosis in adolescents: Ethnic differences and clinical profiles. Schizophr Res. 2013;150:526–532.

629. Duivis HE, Penninx BWJH, Beekman ATF, de Jonge P, Hoogendijk W, Kluft C, et al. Association of depressive disorders, depression characteristics and antidepressant medication with inflammation. Transl Psychiatry. 2012;2:e79–e79.

630. Eaton WW, Mortensen PB, Herrman H, Freeman H, Bilker W, Burgess P, et al. Long-term course of hospitalization for schizophrenia: Part I. Risk for rehospitalization. Schizophr Bull. 1992;18:217—

228.

631. Ebejer JL, Medland SE, Werf J Van Der, Gondro C, Henders AK, Lynskey M, et al. Attention Deficit Hyperactivity Disorder in Australian Adults : Prevalence , Persistence , Conduct Problems and Disadvantage. PLoS ONE Vol7(10), 2012, ArtID E47404. 2012;7.

632. Egeland JA, Blumenthal RL, Nee J, Sharpe L, Endicott J. Reliability and relationship of various ages of onset criteria for major affective disorder. J Affect Disord. 1987;12:159—165.

633. Ehmann TS, Tee KA, Macewan GW, Dalzell KL, Hanson LA, Smith GN, et al. Treatment delay and pathways to care in early psychosis. Early Interv Psychiatry. 2014;8:240–246.

634. El Wasify M, Fawzy M, Barakat D, Youssef U, El Wasify M, Saleh A, et al. The Sociodemographic and Clinical Characteristics of Tramadol Dependence among Egyptians and Their Relationship to the Associated Insomnia. Addict Disord Their Treat. 2018. 2018. https://doi.org/10.1097/ADT.0000000000000129.

635. El-Hadidy MA, Abdeen HM, Abd El-Aziz SM, Al-Harrass M. MTHFR Gene Polymorphism and Age of Onset of Schizophrenia and Bipolar Disorder. Biomed Res Int. 2014;2014:1–9.

636. Elkins IJ, McGue M, Iacono WG. Prospective effects of attention-deficit/hyperactivity disorder, conduct disorder, and sex on adolescent substance use and abuse. Arch Gen Psychiatry. 2007;64:1145—1152.

637. Enander J, Ivanov VZ, Mataix-Cols D, Kuja-Halkola R, Ljótsson B, Lundström S, et al. Prevalence and heritability of body dysmorphic symptoms in adolescents and young adults: a population-based nationwide twin study. Psychol Med. 2018;48:2740—2747.

638. Eriksson Å, Romelsjö A, Stenbacka M, Tengström A. Early risk factors for criminal offending in schizophrenia: A 35-year longitudinal cohort study. Soc Psychiatry Psychiatr Epidemiol. 2011;46:925–932.

639. Evenson RC, Meier ST, Hagan BJ. Sex differences in the age of onset of affective disorders. Compr Psychiatry. 1993;34:187–191.

640. Fang Y-R, Wang G, Chiu HFK, Chen D-F, Wong SYS, Li H-C, et al. Gender differences in demographic and clinical features and prescribing patterns of psychotropic medications in patients with major depressive disorder in China. Compr Psychiatry. 2013;54:1198–1202.

641. Faraone S V, Chen WJ, Goldstein JM, Tsuang MT. Gender Differences in Age at Onset of Schizophrenia. Br J Psychiatry. 1994;164:625–629.

642. Faravelli C, Cosci F, Rotella F, Faravelli L, Catena Dell'osso M. Agoraphobia between panic and phobias: clinical epidemiology from the Sesto Fiorentino Study. Compr Psychiatry. 2008;49:283—287.

643. Fichter MM, Quadflieg N. Mortality in eating disorders - Results of a large prospective clinical longitudinal study. Int J Eat Disord. 2016;49:391–401.

644. Fiedorowicz JG, Leon AC, Keller MB, Solomon DA, Rice JP, Coryell WH. Do risk factors for suicidal behavior differ by affective disorder polarity? Psychol Med. 2009;39:763—771.

645. Fiedorowicz JG, Endicott J, Solomon DA, Keller MB, Coryell WH. Course of illness following prospectively observed mania or hypomania in individuals presenting with unipolar depression. Bipolar Disord. 2012;14:664–671.

646. Fink DS, Calabrese JR, Liberzon I, Tamburrino MB, Chan P, Cohen GH, et al. Retrospective age-of-onset and projected lifetime prevalence of psychiatric disorders among U.S. Army National Guard soldiers. J Affect Disord. 2016;202:171—177.

647. Fontenelle LF, Kohlrausch FB, Melo-Felippe FB, Gomes CKF, de Salles Andrade JB, Vieira-Fonseca T. Association analysis of SLC6A4 and HTR2A genes with obsessive-compulsive disorder: Influence

of the STin2 polymorphism. Compr Psychiatry. 2017;82:1–6.

648. Furberg H, Lichtenstein P, Pedersen NL, Thornton L, Bulik CM, Lerman C, et al. The STAGE cohort: a prospective study of tobacco use among Swedish twins. Nicotine Tob Res. 2008;10:1727—1735.

649. Gadermann AM, Gilman SE, McLaughlin KA, Nock MK, Petukhova M, Sampson NA, et al. Projected Rates of Psychological Disorders and Suicidality Among Soldiers Based on Simulations of Matched General Population Data. Mil Med. 2012. 2012. https://doi.org/10.7205/milmed-d-12-00092.

650. Gaebel W, Schmidt LG, Pfeiffer H, Rüther E, Seemüller F, Möller H-J, et al. One-year functional outcomes of naturalistically treated patients with schizophrenia. Psychiatry Res. 2012;198:378–385.

651. Gallagher D, Mhaolain AN, Greene E, Walsh C, Denihan A, Bruce I, et al. Late life depression: A comparison of risk factors and symptoms according to age of onset in community dwelling older adults. Int J Geriatr Psychiatry. 2010. 2010. https://doi.org/10.1002/gps.2438.

652. Gallagher BJ, Jones BJ. Early-onset schizophrenia: Symptoms and social class of origin. Int J Soc Psychiatry. 2017;63:492–497.

653. Geske J, Crow S, Mori N, Prieto ML, Bond DJ, McElroy SL, et al. Prevalence and correlates of DSM-5 eating disorders in patients with bipolar disorder. J Affect Disord. 2015;191:216–221.

654. Ghisleni G, Jansen K, Gazal M, Silva RA da, Oses JP, Kaster MP, et al. Association of interleukin-10 levels with age of onset and duration of illness in patients with major depressive disorder. Rev Bras Psiquiatr. 2015;37:296–302.

655. Ghosh A, Malhotra S, Basu D. Are childhood externalizing disorders the harbinger of early-onset alcohol dependence? Indian J Med Res. 2016;144:385–392.

656. Giltay EJ, van Rossum EFC, Spijker AT, Zitman FG, Hoencamp E, DeRijk RH, et al. Glucocorticoid and mineralocorticoid receptor polymorphisms and clinical characteristics in bipolar disorder patients. Psychoneuroendocrinology. 2011;36:1460–1469.

657. Glahn DC, Ontiveros A, Medina R, Jerez A, Raventos H, Muñoz R, et al. Heritability of age of onset of psychosis in schizophrenia. Am J Med Genet Part B Neuropsychiatr Genet. 2009;9999B:n/a-n/a.

658. Glasheen C, Richardson GA, Kim KH, Larkby CA, Swartz HA, Day NL. Exposure to maternal pre- and postnatal depression and anxiety symptoms: risk for major depression, anxiety disorders, and conduct disorder in adolescent offspring. Dev Psychopathol. 2013;25:1045—1063.

659. Goes FS, McCusker MG, Bienvenu OJ, MacKinnon DF, Mondimore FM, Schweizer B, et al. Co-morbid anxiety disorders in bipolar disorder and major depression: Familial aggregation and clinical characteristics of co-morbid panic disorder, social phobia, specific phobia and obsessive-compulsive disorder. Psychol Med. 2012;42:1449–1459.

660. Goldstein JM, Santangelo SL, Simpson JC, Tsuang MT. The role of gender in identifying subtypes of schizophrenia: a latent class analytic approach. Schizophr Bull. 1990;16:263—275.

661. Goodwin RD, Hamilton SP. The early-onset fearful panic attack as a predictor of severe psychopathology. Psychiatry Res. 2002;109:71–79.

662. Gradus JL, Bozi I, Antonsen S, Svensson E, Lash TL, Resick PA, et al. Severe stress and adjustment disorder diagnoses in the population of Denmark. J Trauma Stress. 2014;27:370—374.

663. Grant BF, Dawson DA. Age of onset of drug use and its association with DSM-IV drug abuse and dependence: Results from the national longitudinal alcohol epidemiologic survey. J Subst Abuse. 1998;10:163–173.

664. Grant JE, Odlaug BL, Chamberlain SR. Gambling disorder, DSM-5 criteria and symptom severity. Compr Psychiatry. 2017;75:1–5.

665. Green AC, Hunt C, Stain HJ. The delay between symptom onset and seeking professional treatment for anxiety and depressive disorders in a rural Australian sample. Soc Psychiatry Psychiatr Epidemiol. 2012;47:1475–1487.

666. Grigoroiu-Serbanescu M, Diaconu CC, Heilmann-Heimbach S, Neagu AI, Becker T. Association of age-of-onset groups with GWAS significant schizophrenia and bipolar disorder loci in Romanian bipolar I patients. Psychiatry Res. 2015;230:964–967.

667. Grove J, Vares M, Agartz I, Saetre P, Andreassen OA, Terenius L, et al. Methylenetetrahydrofolate reductase (MTHFR) C677T polymorphism and age at onset of schizophrenia: No consistent evidence for an association in the nordic population . Am J Med Genet Part B Neuropsychiatr Genet. 2012;159B:981–986.

668. Gundogdu C, Oguzoncul AF. The relationship between smoking and exercise among physical education teachers in Turkey. Southeast Asian J Trop Med Public Health. 2013;44:712–717.

669. Gupta G, Kate N, Avasthi A, Sarkar S, Chakrabarti S, Grover S, et al. Factor analysis of symptom profile in early onset and late onset OCD. Psychiatry Res. 2017;262:631–635.

670. Gur RC, Zackai EH, McDonald-McGinn DM, Moore TM, Kohler CG, Savitt A, et al. The Psychosis Spectrum in 22q11.2 Deletion Syndrome Is Comparable to That of Nondeleted Youths. Biol Psychiatry. 2016;82:17–25.

671. Gureje O. Gender and schizophrenia: age at onset and sociodemographic attributes. Acta Psychiatr Scand. 1991;83:402–405.

672. Heinz Hafner, Stephan Behrens, Jean De Vry WFG. Oestradiol Enhances the Vulnerability Threshold for Schizophrenia in Women by an Early Effect on Dopaminergic Neurotransmission Evidence from an Epidemiological Study and from Animal Experiments. Eur Arch Psychiatry Clin Neurosci 241(1)65-8, 1991. 1991:65–68.

673. Häfner H, Riecher-Rössler A, An Der Heiden W, Maurer K, Fätkenheuer B, Löffler W. Generating and testing a causal explanation of the gender difference in age at first onset of schizophrenia. Psychol Med. 1993;23:925—940.

674. Hakko H, Räsänen P, Timonen M, von Wendt L, Zitting P, Veijola J, et al. The association of preceding traumatic brain injury with mental disorders, alcoholism and criminality: the Northern Finland 1966 Birth Cohort Study. Psychiatry Res. 2003;113:217–226.

675. Hall A, Hay PJ. Eating disorder patient referrals from a population region 1977-1986. Psychol Med. 1991;21:697—701.

676. Hambrecht M, Maurer K, Häfner H, Sartorius N. Transnational stability of gender differences in schizophrenia? An analysis based on the WHO study on determinants of outcome of severe mental disorders. Eur Arch Psychiatry Clin Neurosci. 1992;242:6—12.

677. Hasler BP, Kirisci L, Clark DB. Restless Sleep and Variable Sleep Timing During Late Childhood Accelerate the Onset of Alcohol and Other Drug Involvement. J Stud Alcohol Drugs. 2016;77:649–655.

678. Hayatbakhsh MR, McGee TR, Bor W, Najman JM, Jamrozik K, Mamun AA. Child and adolescent externalizing behavior and cannabis use disorders in early adulthood: An Australian prospective birth cohort study. Addict Behav. 2008;33:422–438.

679. Heinrich A, Nieratschker V, Hovatta I, Frank J, Breuer R, Rietschel M, et al. Longer telomere length in patients with schizophrenia. Schizophr Res. 2013;149:116–120.

680. Hickling LM, Perez-Iglesias R, Ortiz-García de la Foz V, Balanzá-Martínez V, McGuire P, Crespo-Facorro B, et al. Tobacco smoking and its association with cognition in first episode psychosis patients. Schizophr Res. 2018;192:269–273.

681. Hirneth SJ, Hazell PL, Hanstock TL, Lewin TJ. Bipolar disorder subtypes in children and adolescents: Demographic and clinical characteristics from an Australian sample. J Affect Disord. 2015;175:98–107.

682. Hoffmann JP, Baldwin SA, Cerbone FG. Onset of major depressive disorder among adolescents. J Am Acad Child Adolesc Psychiatry. 2003. 2003. https://doi.org/10.1097/00004583-200302000-00016.

683. Hsu SE, Chin Chen K, Lee LT, Chun Tsai H, Lee IH, See Chen P, et al. Comparison of cognitive deficits among drug-naive patients with schizophrenia and major depressive disorder. J Affect Disord. 2015;175:133–138.

684. Hulkko AP, Murray GK, Moilanen J, Haapea M, Rannikko I, Jones PB, et al. Lifetime use of psychiatric medications and cognition at 43 years of age in schizophrenia in the Northern Finland Birth Cohort 1966. Eur Psychiatry. 2017. 2017. https://doi.org/10.1016/j.eurpsy.2017.06.004.

685. Hybels CF, Landerman LR, Blazer DG. Age differences in symptom expression in patients with major depression. Int J Geriatr Psychiatry. 2012;27:601–611.

686. Ifabumuyi OI, Akindele MO. Post-partum mental illness in northern Nigeria. Acta Psychiatr Scand. 1985;72:63—68.

687. Iga J, Nishi A, Umehara H, Watanabe S, Ohmori T, Numata S, et al. Polymorphism in the promoter of the gene for the serotonin transporter affects the age of onset of major depressive disorder in the Japanese population. J Affect Disord. 2015;183:156–158.

688. Jääskeläinen M, Holmila M, Notkola IL, Raitasalo K. Mental disorders and harmful substance use in children of substance abusing parents: A longitudinal register-based study on a complete birth cohort born in 1991. Drug Alcohol Rev. 2016;35:728–740.

689. Javaras KN, Laird NM, Reichborn-Kjennerud T, Bulik CM, Pope HG, Hudson JI. Affect modulates appetite-related brain activity. Int J Eat Disord. 2008;41:174–179.

690. Jenkins PE, Price T. Eating pathology in midlife women: Similar or different to younger counterparts? Int J Eat Disord. 2018;51:3–9.

691. Jhanda S, Malhotra S, Grover S. Relationship between bipolar disorder and attention deficit hyperkinetic disorder: An exploratory study. Asian J Psychiatr. 2018;35:101–108.

692. Jia F-J, Cao X-L, Hou C-L, Shinfuku N, Zhong B-L, Lin Y-Q, et al. Clozapine Prescription and Quality of Life in Chinese Patients with Schizophrenia Treated in Primary Care. Pharmacopsychiatry. 2015;48:200–204.

693. Jiménez-Murcia S, Álvarez-Moya EM, Stinchfield R, Fernández-Aranda F, Granero R, Aymamí N, et al. Age of onset in pathological gambling: Clinical, therapeutic and personality correlates. J Gambl Stud. 2010;26:235–248.

694. Jiménez-Murcia S, Granero R, Tárrega S, Angulo A, Fernández-Aranda F, Arcelus J, et al. Mediational Role of Age of Onset in Gambling Disorder, a Path Modeling Analysis. J Gambl Stud. 2016;32:327–340.

695. Jiménez-Murcia S, Granero R, Aymamí MN, Santamaría J, Menchón JM, Álvarez-Moya EM, et al. Subtyping Study of a Pathological Gamblers Sample. Can J Psychiatry. 2017;55:498–506.

696. Johnson CL, Love SQ. Bulimia: multivariate predictors of life impairment. J Psychiatr Res. 1985;19:343—347.

697. Joshi V, Grella CE, Hser YI. Drug use and treatment initiation patterns: Differences by birth-cohorts. J Drug Issues. 2001. 2001. https://doi.org/10.1177/002204260103100412.

698. Kamali M, Mctigue O, Whitty P, Gervin M, Clarke M, Browne S, et al. Lifetime history of substance misuse in first-episode psychosis: Prevalence and its influence on psychopathology and onset of

psychotic symptoms. Early Interv Psychiatry. 2009;3:198–203.

699. Katerberg H, Cath DC, Denys DAJP, Heutink P, Polman A, Van Nieuwerburgh FCW, et al. The role of the COMT Val158 met polymorphism in the phenotypic expression of obsessive-compulsive disorder. Am J Med Genet Part B Neuropsychiatr Genet. 2010;153:167–176.

700. Kecmanović M, Dobričić V, Dimitrijević R, Keckarević D, Savić-Pavićević D, Keckarević-Marković M, et al. Schizophrenia and apolipoprotein e gene polymorphism in Serbian population. Int J Neurosci. 2010;120:502–506.

701. Kelly MM, Dalrymple K, Zimmerman M, Phillips KA. A comparison study of body dysmorphic disorder versus social phobia. Psychiatry Res. 2013;205:109–116.

702. Kenardy J, Oei TPS, Evans L. Neuroticism and age of onset for agoraphobia with panic attacks. J Behav Ther Exp Psychiatry. 1990;21:193–197.

703. Kendler KS, Neale MC, Kessler RC, Heath AC, Eaves LJ. The Genetic Epidemiology of Phobias in Women The Interrelationship of Agoraphobia, Social Phobia, Situational Phobia, and Simple Phobia. Arch Gen Psychiatry 49(4)273-81, 1992 Apr. 1992. 1992.

704. Kendler KS, Gatz M, Gardner CO, Pedersen NL. Age at onset and familial risk for major depression in a Swedish national twin sample. Psychol Med. 2005;35:1573—1579.

705. Kendler KS, Gatz M, Gardner CO, Pedersen NL. Clinical indices of familial depression in the Swedish Twin Registry. Acta Psychiatr Scand. 2007;115:214—220.

706. Kendler KS, Karkowski-Shuman L, Walsh D. Age at Onset in Schizophrenia and Risk of Illness in Relatives. Br J Psychiatry. 2008;169:213–218.

707. Kendler KS, Myers J, Zisook S. Does bereavement-related major depression differ from major depression associated with other stressful life events? Am J Psychiatry. 2008;165:1449—1455.

708. Kenneth S. Kendler, M.D., John Myers, M.S., M. Imad Damaj, Ph.D., and Xianging Chen PD. Early Smoking Onset and Risk for Subsequent Nicotine Dependence: A Monozygotic Co-Twin Control Study Kenneth. Am J Psychiatry Vol170(4), 2013, Pp 408-413. 2013;170:408–413.

709. Kessler RC, Heeringa SG, Stein MB, Colpe LJ, Fullerton CS, Hwang I, et al. Thirty-day prevalence of DSM-IV mental disorders among nondeployed soldiers in the US Army: results from the Army Study to Assess Risk and Resilience in Servicemembers (Army STARRS). JAMA Psychiatry. 2014;71:504—513.

710. Ketter TA, Rasgon NL, Wang PW, Holtzman JN, Chang KD, Miller S, et al. Childhood-compared to adolescent-onset bipolar disorder has more statistically significant clinical correlates. J Affect Disord. 2015;179:114–120.

711. Kienzle J. Predictors of smoking initiation in African American adolescents Predictors of smoking initiation in African American adolescents Downloaded from Downloaded from. 2009.

712. Kiezebrink K, Campbell D, Mann E, Blundell J. Similarities and differences between excessive exercising anorexia nervosa patients compared with DSM-IV defined anorexia nervosa subtypes. Eat Weight Disord. 2009;14:199–204.

713. Kinasz K, Accurso EC, Kass AE, Le Grange D. Does Sex Matter in the Clinical Presentation of Eating Disorders in Youth? J Adolesc Heal. 2016;58:410–416.

714. Kovacs M, Feinberg TL, Crouse-Novak MA, Paulauskas SL, Finkelstein R. Depressive disorders in childhood. I. A longitudinal prospective study of characteristics and recovery. Arch Gen Psychiatry. 1984;41:229—237.

715. Krausz M, Müller-Thomsen T. Schizophrenia with onset in adolescence: an 11-year followup. Schizophr Bull. 1993;19:831—841.

716. Kuo P-H, Gardner CO, Kendler KS, Prescott CA. The temporal relationship of the onsets of alcohol dependence and major depression: using a genetically informative study design. Psychol Med. 2006;36:1153—1162.

717. Kuo SC, Yeh YW, Chen CY, Huang CC, Chang HA, Yen CH, et al. DRD3 variation associates with early-onset heroin dependence, but not specific personality traits. Prog Neuro-Psychopharmacology Biol Psychiatry. 2014;51:1–8.

718. Larson CA, Nyman GE. Schizophrenia: outcome in a birth year cohort. Psychiatr Clin (Basel). 1974;7:50—55.

719. Lavori PW, Klerman GL, Keller MB, Reich T, Rice J, Endicott J. Age-period-cohort analysis of secular trends in onset of major depression: findings in siblings of patients with major affective disorder. J Psychiatr Res. 1987;21:23—35.

720. Lavori PW, Warshaw M, Klerman G, Mueller TI, Leon A, Rice J, et al. Secular trends in lifetime onset of MDD stratified by selected sociodemographic risk factors. J Psychiatr Res. 1993;27:95—109.

721. Lee LO, Wolff KCY, Kendler KS, Prescott CA. The Effects of Age at Drinking Onset and Stressful Life Events on Alcohol Use in Adulthood : A Replication and Extension Using a Population-Based Twin Sample. Alcohol Clin Exp Res Vol36(4), 2012, Pp 693-704. 2012;36:693–704.

722. Lesch O, Djordjevic N, Walter H, Patek K, Jovanovic M, Jakovljevic M, et al. Serbian and Austrian Alcohol-Dependent Patients: A Comparison of Two Samples Regarding Therapeutically Relevant Clinical Features. Alcohol Alcohol. 2013;48:505–508.

723. Lewitzka U, Rasgon N, Whybrow PC, Sagduyu K, Munoz R, Bauer M, et al. Association between age of onset and mood in bipolar disorder: Comparison of subgroups identified by cluster analysis and clinical observation. J Psychiatr Res. 2010;44:1170–1175.

724. Li Q, Xiang YT, Su YA, Shu L, Yu X, Correll CU, et al. Clozapine in schizophrenia and its association with treatment satisfaction and quality of life: Findings of the three national surveys on use of psychotropic medications in China (2002-2012). Schizophr Res. 2015;168:523–529.

725. Li Q, Xiang YT, Su YA, Shu L, Yu X, Chiu HFK, et al. Antipsychotic polypharmacy in schizophrenia patients in China and its association with treatment satisfaction and quality of life: Findings of the third national survey on use of psychotropic medications in China. Aust N Z J Psychiatry. 2015;49:129–136.

726. Li Q, Su YA, Xiang YT, Shu L, Yu X, Ungvari GS, et al. Adjunctive antidepressant use in schizophrenia in China: A national survey (2002–2012). Hum Psychopharmacol. 2017;32:1–5.

727. Lieb R, Brückl T, Pfister H, Wittchen H-U, Beesdo K, Nocon A, et al. Differential familial liability of panic disorder and agoraphobia. Depress Anxiety. 2007;25:422–434.

728. Lin CH, Tsai SJ, Yu YW, Yang KH, Hsu CP, Hong CJ. Study of anticipation in Chinese families with schizophrenia. Psychiatry Clin Neurosci. 2001;55:137—140.

729. Lin GM, Chen YJ, Kuo DJ, Jaiteh LES, Wu YC, Lo TS, et al. Cancer incidence in patients with schizophrenia or bipolar disorder: A nationwide population-based study in Taiwan, 1997-2009. Schizophr Bull. 2013. 2013. https://doi.org/10.1093/schbul/sbr162.

730. Liu I-C, Blacker DL, Xu R, Fitzmaurice G, Lyons MJ, Tsuang MT. Genetic and environmental contributions to the development of alcohol dependence in male twins. Arch Gen Psychiatry. 2004;61:897—903.

731. Liu YH, Chen L, Su YA, Fang YR, Srisurapanont M, Hong JP, et al. Is early-onset in major depression a predictor of specific clinical features with more impaired social function? Chin Med J (Engl). 2015;128:811–815.

732. Lydecker JA, Grilo CM. Comparing men and women with binge-eating disorder and co-morbid obesity. Int J Eat Disord. 2018;51:411–417.

733. Lynskey MT, Bucholz KK, Madden PAF, Heath AC. Early-onset alcohol-use behaviors and subsequent alcohol-related driving risks in young women: a twin study. J Stud Alcohol Drugs. 2007;68:798—804.

734. Lynskey MT, Agrawal A, Henders A, Nelson EC, Madden PAF, Martin NG. An Australian twin study of cannabis and other illicit drug use and misuse, and other psychopathology. Twin Res Hum Genet. 2012;15:631—641.

735. Macciardi F, Holmgren S, Perris H, Knorring L, Perris C, Smeraldi E. Age at onset of affective disorders in Italian and Swedish patients. Acta Psychiatr Scand. 2007;75:352–357.

736. Mäki P, Koskela S, Murray GK, Nordström T, Miettunen J, Jääskeläinen E, et al. Difficulty in making contact with others and social withdrawal as early signs of psychosis in adolescents-the Northern Finland Birth Cohort 1986. Eur Psychiatry. 2014;29:345–351.

737. Mäkikyrö T, Isohanni M, Moring J, Oja H, Hakko H, Jones P, et al. Is a child's risk of early onset schizophrenia increased in the highest social class? Schizophr Res. 1997;23:245–252.

738. Mann JJ, Burke AK, Poh E, Galfalvy H, Oquendo MA, Grunebaum MF, et al. Sex differences in clinical predictors of depression: A prospective study. J Affect Disord. 2013;150:1179–1183.

739. Marel C, Sunderland M, Mills KL, Slade T, Teesson M, Chapman C. Conditional probabilities of substance use disorders and associated risk factors: Progression from first use to use disorder on alcohol, cannabis, stimulants, sedatives and opioids. Drug Alcohol Depend. 2019;194:136—142.

740. Marie D, Fergusson DM, Boden JM. Results of a 25 Year Longitudinal Study. Ethnicity. 2008. 2008.

741. Markkula N, Lehti V, Gissler M, Suvisaari J. Incidence and prevalence of mental disorders among immigrants and native Finns: a register-based study. Soc Psychiatry Psychiatr Epidemiol. 2017;52:1523–1540.

742. Masmoudi J, Baati I, Sellami R, Trigui D, Feki I, Moalla M. Impulsivity in bipolar disorders in a Tunisian sample. Asian J Psychiatr. 2016;22:77–80.

743. Mechri A, Slama H, Gaha L, Gassab L, Aissi M. Prevalence and score of minor physical anomalies in patients with schizophrenia and their first degree relatives: A Tunisian study. Compr Psychiatry. 2013;54:575–580.

744. Menezes PR, Mann AH. Characteristics of hospital-treated schizophrenia in Sao Paulo, Brazil. Soc Psychiatry Psychiatr Epidemiol 28(6)267-74, 1993 Nov. 1993:267–274.

745. Moore EA, Green MJ, Carr VJ. Comorbid personality traits in schizophrenia: Prevalence and clinical characteristics. J Psychiatr Res. 2012;46:353–359.

746. Mork E, Walby FA, Harkavy-Friedman JM, Barrett EA, Steen NE, Lorentzen S, et al. Clinical characteristics in schizophrenia patients with or without suicide attempts and non-suicidal self-harm - A cross-sectional study. BMC Psychiatry. 2013;13:2–9.

747. Moss HB, Chen CM, Yi HY. DSM-IV criteria endorsement patterns in alcohol dependence: Relationship to severity. Alcohol Clin Exp Res. 2008;32:306–313.

748. Mustelin L, Silén Y, Raevuori A, Hoek HW, Kaprio J, Keski-Rahkonen A. The DSM-5 diagnostic criteria for anorexia nervosa may change its population prevalence and prognostic value. J Psychiatr Res. 2016;77:85–91.

749. Myles-Worsley M, Coon H, Tiobech J, Collier J, Dale P, Wender P, et al. Genetic epidemiological study of schizophrenia in Palau, Micronesia: Prevalence and familiality. Am J Med Genet - Neuropsychiatr Genet. 1999;88:4–10.

750. Nair MKC, Russell PSS, Krishnan R, Russell S, Subramaniam VS, Nazeema S, et al. ADad 4: The Symptomatology and Clinical Presentation of Anxiety Disorders Among Adolescents in a Rural Community Population in India. Indian J Pediatr. 2013;80:149–154.

751. Naji L, Dennis BB, Bawor M, Varenbut M, Daiter J, Plater C, et al. The association between age of onset of opioid use and comorbidity among opioid dependent patients receiving methadone maintenance therapy. Addict Sci Clin Pract. 2017;12:9.

752. Neuman RJ, Sitdhiraksa N, Reich W, Ji TH-C, Joyner CA, Sun L-W, et al. Estimation of prevalence of DSM-IV and latent class-defined ADHD subtypes in a population-based sample of child and adolescent twins. Twin Res Hum Genet. 2005;8:392—401.

753. Newman SC, Bland RC. Incidence of mental disorders in Edmonton: Estimates of rates and methodological issues. J Psychiatr Res. 1998;32:273–282.

754. Niles AN, Lebeau RT, Liao B, Glenn DE, Craske MG. Dimensional indicators of generalized anxiety disorder severity for DSM-V. J Anxiety Disord. 2012;26:279–286.

755. Ohaeri JU. Age at onset in a cohort of schizophrenics in Nigeria. Acta Psychiatr Scand. 1992;86:332—334.

756. Oncel SY, Dick DM, Maes HH. Risk Factors Influencing Smoking Behavior : A Turkish Twin Study. Twin Res Hum Genet Vol17(6), 2014, Pp 563-573. 2014;17:563–573.

757. Oostervink F, Nolen WA, Kok RM. Two years’ outcome of acute mania in bipolar disorder: Different effects of age and age of onset. Int J Geriatr Psychiatry. 2015;30:201–209.

758. Ortiz-García de la Foz V, Crespo-Facorro B, Perez-Iglesias R, Hickling LM, McGuire P, Ayesa-Arriola R. The effects of tobacco smoking on age of onset of psychosis and psychotic symptoms in a first-episode psychosis population. Addiction. 2016;112:526–532.

759. Orvaschel H, Thompson WD, Belanger A, Prusoff BA, Kidd KK. Comparison of the family history method to direct interview. Factors affecting the diagnosis of depression. J Affect Disord. 1982;4:49—59.

760. Oude RC, Kapur N, Bickley H, Williams A, Purandare N. Suicide in later life : A comparison between cases with early-onset and late-onset depression ☆. J Affect Disord. 2011;132:185–191.

761. Park J-I, Won S, Lee D-W, Lee J-Y, Lee YM, Bae JN, et al. Lifetime risk and age of onset distributions of psychiatric disorders: analysis of national sample survey in South Korea. Soc Psychiatry Psychiatr Epidemiol. 2011;47:671–681.

762. Park SC, Hahn SW, Hwang TY, Kim JM, Jun TY, Lee MS, et al. Does age at onset of first major depressive episode indicate the subtype of major depressive disorder?: The clinical research center for depression study. Yonsei Med J. 2014;55:1712–1720.

763. Parker G, Graham R, Hadzi-Pavlovic D, McCraw S, Hong M, Friend P. Differentiation of bipolar i and II disorders by examining for differences in severity of manic/hypomanic symptoms and the presence or absence of psychosis during that phase. J Affect Disord. 2013;150:941–947.

764. Paruk S, Jhazbhay K, Singh K, Sartorius B, Burns JK. Clinical correlates of first episode early onset psychosis in KwaZulu-Natal, South Africa. J Child Adolesc Ment Health. 2015;27:103–111.

765. Pawlak J, Zaremba D, Czerski P, Kapelski P, Rajewska-Rager A, Miechowicz I, et al. Are suicide risk factors gender specific? Psychiatr Pol. 2018;52:21–32.

766. Pedersen J, Aarkrog T. A 20-year study of an adolescent psychiatric clientele, with special reference to the age of onset. Nord J Psychiatry. 2001;55:5–10.

767. Penninx BWJH, Giltay EJ, Milaneschi Y, Thesing CS, Bot M. Omega-3 and omega-6 fatty acid levels in depressive and anxiety disorders. Psychoneuroendocrinology. 2017;87:53–62.

768. Petruzzelli MG, Margari L, Bosco A, Craig F, Palumbi R, Margari F. Early onset first episode psychosis: dimensional structure of symptoms, clinical subtypes and related neurodevelopmental markers. Eur Child Adolesc Psychiatry. 2018;27:171–179.

769. Pickens RW, Svikis DS, Mcgue M, Lykken DT, Heston LL, Clayton PJ. Heterogeneity in the Inheritance of Alcoholism. Arch Gen Psychiatry 48(1)19-28, 1991 Jan. 1991;21224.

770. Poon JYK, Leung CM. Outcome of first-episode acute and transient psychotic disorder in Hong Kong Chinese: a 20-year retrospective follow-up study. Nord J Psychiatry. 2017;71:139–144.

771. Post RM, Altshuler LL, Kupka R, Mcelroy SL, Frye MA, Rowe M, et al. Verbal abuse, like physical and sexual abuse, in childhood is associated with an earlier onset and more difficult course of bipolar disorder. Bipolar Disord. 2015;17:323–330.

772. Post RM, Altshuler LL, Kupka R, McElroy SL, Frye MA, Rowe M, et al. More illness in offspring of bipolar patients from the U.S. compared to Europe. J Affect Disord. 2016;191:180–186.

773. Poudel A, Gautam S. Age of onset of substance use and psychosocial problems among individuals with substance use disorders. BMC Psychiatry. 2017;17:1–7.

774. Prata D, Morgan C, La Cascia C, Reis Marques T, Stahl D, Trotta A, et al. Daily Use, Especially of High-Potency Cannabis, Drives the Earlier Onset of Psychosis in Cannabis Users. Schizophr Bull. 2013;40:1509–1517.

775. Preisig M, Strippoli MPF, Castelao E, Merikangas KR, Gholam-Rezaee M, Marquet P, et al. The specificity of the familial aggregation of early-onset bipolar disorder: A controlled 10-year follow-up study of offspring of parents with mood disorders. J Affect Disord. 2016;190:26–33.

776. Pulver AE, Brown CH, Wolyniec P, McGrath J, Tam D, Adler L, et al. Schizophrenia: age at onset, gender and familial risk. Acta Psychiatr Scand. 1990;82:344—351.

777. Qian W, Huo Z, Lu H, Sheng Y, Geng Z, Ma Z. Digit ratio (2D:4D) in a Chinese population with schizophrenia. Early Hum Dev. 2016;98:45–48.

778. Queirazza F, Semple DM, Lawrie SM. Transition to schizophrenia in acute and transient psychotic disorders. Br J Psychiatry. 2014;204:299—305.

779. Ramirez A, Ekselius L, Ramklint M. Depression in young adult psychiatric outpatients: Delimiting early onset. Early Interv Psychiatry. 2015;9:108–117.

780. Rasgon NL, Hooshmand F, Chang KD, Miller S, Goffin KC, Hill SJ, et al. Gender by onset age interaction may characterize distinct phenotypic subgroups in bipolar patients. J Psychiatr Res. 2016;76:128–135.

781. Ridley RM, Baker HF. Implications of age of onset for the genetics of schizophrenia. Biol Psychiatry. 1990;28:455–458.

782. Ritsner MS, Strous RD. Neurocognitive deficits in schizophrenia are associated with alterations in blood levels of neurosteroids: A multiple regression analysis of findings from a double-blind, randomized, placebo-controlled, crossover trial with DHEA. J Psychiatr Res. 2010;44:75–80.

783. Ritter P, Viswanath B, Berk M, Belmaker RH, Henry C, Larsen ER, et al. Relationship between sunlight and the age of onset of bipolar disorder: An international multisite study. J Affect Disord. 2014;167:104–111.

784. Robison EJ, Shankman SA, McFarland BR. Independent Associations Between Personality Traits and Clinical Characteristics of Depression. J Nerv Ment Dis. 2009;197:476–483.

785. Rognli EB, Håkansson A, Berge J, Bramness JG. Does the pattern of amphetamine use prior to incarceration predict later psychosis?--a longitudinal study of amphetamine users in the Swedish criminal justice system. Drug Alcohol Depend. 2014;143:219—224.

786. Rubio-Abadal E, Ochoa S, Barajas A, Baños I, Dolz M, Sanchez B, et al. Birth weight and obstetric complications determine age at onset infirst episode of psychosis. J Psychiatr Res. 2015;65:108–114.

787. Saewyc EM, Bearinger LH, Heinz PA, Blum RW, Resnick MD. Gender differences in health and risk behaviors among bisexual and homosexual adolescents. J Adolesc Health. 1998;23:181—188.

788. Sartor CE, Jackson KM, McCutcheon V V, Duncan AE, Grant JD, Werner KB, et al. Progression from First Drink, First Intoxication, and Regular Drinking to Alcohol Use Disorder: A Comparison of African American and European American Youth. Alcohol Clin Exp Res. 2016;40:1515—1523.

789. Sathyan S, Nair CM, Saradalekshmi KR, Banerjee M, Nair I V., Neetha NV. DNA Methyl Transferase (DNMT) Gene Polymorphisms Could Be a Primary Event in Epigenetic Susceptibility to Schizophrenia. PLoS One. 2014;9:e98182.

790. Schandrin A, Chereau I, Fond G, Urbach M, Le Strat Y, Lancon C, et al. Differential effects of childhood trauma and cannabis use disorders in patients suffering from schizophrenia. Schizophr Res. 2016;175:161–167.

791. Schimmelmann BG, Conus P, Cotton S, McGorry PD, Lambert M. Pre-treatment, baseline, and outcome differences between early-onset and adult-onset psychosis in an epidemiological cohort of 636 first-episode patients. Schizophr Res. 2007;95:1–8.

792. Segarra R, Ojeda N, Zabala A, García J, Catalán A, Eguíluz JI, et al. Similarities in early course among men and women with a first episode of schizophrenia and schizophreniform disorder. Eur Arch Psychiatry Clin Neurosci. 2012;262:95–105.

793. Seo HJ, Jung YE, Kim TS, Kim JB, Lee MS, Kim JM, et al. Distinctive clinical characteristics and suicidal tendencies of patients with anxious depression. J Nerv Ment Dis. 2011;199:42–48.

794. Serretti A, Chiesa A, Calati R, Linotte S, Sentissi O, Papageorgiou K, et al. Influence of family history of major depression, bipolar disorder, and suicide on clinical features in patients with major depression and bipolar disorder. Eur Arch Psychiatry Clin Neurosci. 2013;263:93–103.

795. Sharp CW, Clark SA, Dunan JR, Blackwood DH, Shapiro CM. Clinical presentation of anorexia nervosa in males: 24 new cases. Int J Eat Disord. 1994;15:125—134.

796. Berner LA, Lowe MR, Franko DL, Clark VL, Shaw JA, Eddy KT, et al. Elevated pre-morbid weights in bulimic individuals are usually surpassed post-morbidly: Implications for perpetuation of the disorder. Int J Eat Disord. 2012;45:512–523.

797. Shi Q, Susser E, Li X, Song Z, Phillips MR, Ding Z, et al. Marriage outcome and relationship with urban versus rural context for individuals with psychosis in a population-based study in China. Soc Psychiatry Psychiatr Epidemiol. 2015;50:1501–1509.

798. Shim IH, Woo YS, Bahk WM. Prevalence rates and clinical implications of bipolar disorder 'with mixed features' as defined by DSM-5. J Affect Disord. 2015;173:120–125.

799. Shimshoni Y, Reuven O, Dar R, Hermesh H. Insight in obsessive-compulsive disorder: A comparative study of insight measures in an Israeli clinical sample. J Behav Ther Exp Psychiatry. 2011;42:389–396.

800. Sibisi CDT. Sex differences in the age of onset of bipolar affective illness. Br J Psychiatry. 1990. 1990. https://doi.org/10.1192/bjp.156.6.842.

801. Silveira CM, Viana MC, Siu ER, de Andrade AG, Anthony JC, Andrade LH. Sociodemographic correlates of transitions from alcohol use to disorders and remission in the São Paulo megacity mental health survey, Brazil. Alcohol Alcohol. 2011;46:324–332.

802. Skodol AE, Gunderson JG, Shea MT, McGlashan TH, Morey LC, Sanislow CA, et al. The Collaborative Longitudinal Personality Disorders Study (CLPS): overview and implications. J Pers Disord. 2005;19:487–504.

803. Sørensen HJ, Gamborg M, Sørensen TIA, Baker JL, Mortensen EL. Childhood body mass index and risk of schizophrenia in relation to childhood age, sex and age of first contact with schizophrenia. Eur Psychiatry. 2016;34:64–69.

804. Southwick SM, Sareen J, Tsai J, el-Gabalawy R, Pietrzak RH, Neumeister A. Typologies of posttraumatic stress disorder in the U.S. adult population. J Affect Disord. 2014;162:102–106.

805. Stassen HH, Scharfetter C, Angst J. Morbid risks of subgroups of affective disorders: Some methodological and empirical results. J Psychiatr Res. 1987. 1987. https://doi.org/10.1016/0022-3956(87)90081-1.

806. Statham DJ, Deutsch AR, Piasecki TM, Slutske WS, Martin NG. Telescoping and gender differences in the time course of disordered gambling: evidence from a general population sample. Addiction. 2014;110:144–151.

807. Stefanis NC, Dragovic M, Power BD, Jablensky A, Castle D, Morgan VA. Age at Initiation of Cannabis Use Predicts Age at Onset of Psychosis : The 7- to 8-Year Trend. Schizophr Bull Vol39(2), 2013, Pp 251-254. 2013;39:251–254.

808. Stegenga BT, Nazareth I, Grobbee DE, Torres-gonzález F, Igor Š, Maaroos H, et al. Recent life events pose greatest risk for onset of major depressive disorder during mid-life. 2012;136:505–513.

809. Steinberg DL. Predictors of relapse and recurrence in depression: The scar hypothesis and the impact of depressive episodes. 1999.

810. Subramaniam M, Rekhi G, Chong SA, Vaingankar JA, Abdin E. Age of onset of life-time mental disorders and treatment contact. Soc Psychiatry Psychiatr Epidemiol. 2012;48:835–843.

811. Suchanek R, Owczarek A, Kowalski J. Association study between BDNF C-281A polymorphism and paranoid schizophrenia in Polish population. J Mol Neurosci. 2012;46:217–222.

812. Sugranyes G, Flamarique I, Parellada E, Baeza I, Goti J, Fernandez-Egea E, et al. Cannabis use and age of diagnosis of schizophrenia. Eur Psychiatry. 2009;24:282–286.

813. Sylvia LG, Shelton RC, Kemp DE, Bernstein EE, Friedman ES, Brody BD, et al. Medical burden in bipolar disorder: Findings from the Clinical and Health Outcomes Initiative in Comparative Effectiveness for Bipolar Disorder study (Bipolar CHOICE). Bipolar Disord. 2015;17:212–223.

814. Szerman N, Babin F, Ochoa E, Vega P, Basurte I, Morant C, et al. Cocaine abuse or dependency and other psychiatric disorders. Madrid study on dual pathology. Rev Psiquiatr y Salud Ment (English Ed. 2013;6:121–128.

815. Tabarés-Seisdedos R, Ayesa-Arriola R, Crespo-Facorro B, de la Ortiz-García de la Foz V, Martínez-García O, Gajardo Galán VG, et al. Rates and predictors of relapse in first-episode non-affective psychosis: a 3-year longitudinal study in a specialized intervention program (PAFIP). Eur Arch Psychiatry Clin Neurosci. 2016;267:315–323.

816. Tamás Z, Gentzler AL, Kapornai K, Mayer L, Tepper P, Vetró Á, et al. Early developmental characteristics and features of major depressive disorder among child psychiatric patients in Hungary. J Affect Disord. 2006;100:91–101.

817. Tanskanen P, Haapea M, Veijola J, Miettunen J, Järvelin M, Pyhtinen J, et al. Volumes of brain , grey and white matter and cerebrospinal fl uid in schizophrenia in the Northern Finland 1966 Birth Cohort : An epidemiological approach to analysis. Psychiatry Res Neuroimaging. 2009;174:116–120.

818. Timberlake DS, Haberstick BC, Hopfer CJ, Bricker J, Sakai JT, Lessem JM, et al. Progression from marijuana use to daily smoking and nicotine dependence in a national sample of U.S. adolescents. Drug Alcohol Depend. 2007;88:272—281.

819. Todd RD, Huang H, Henderson CA. Poor utility of the age of onset criterion for DSM-IV attention deficit/hyperactivity disorder: recommendations for DSM-V and ICD-11. J Child Psychol Psychiatry.

2008;49:942—949.

820. Tondo L, Lepri B, Cruz N, Baldessarini RJ. Age at onset in 3014 Sardinian bipolar and major depressive disorder patients. Acta Psychiatr Scand. 2010;121:446–452.

821. Tondo L, Visioli C, Preti A, Baldessarini RJ. Bipolar disorders following initial depression: Modeling predictive clinical factors. J Affect Disord. 2014;167:44–49.

822. Torres US, Duran FLS, Schaufelberger MS, Crippa JAS, Louzã MR, Sallet PC, et al. Patterns of regional gray matter loss at different stages of schizophrenia: A multisite, cross-sectional VBM study in first-episode and chronic illness. NeuroImage Clin. 2016;12:1–15.

823. Trim RS, Schuckit MA, Smith TL. The relationships of the level of response to alcohol and additional characteristics to alcohol use disorders across adulthood: A discrete-time survival analysis. Alcohol Clin Exp Res. 2009;33:1562–1570.

824. Truong W, Minuzzi L, Soares CN, Frey BN, Evans AC, MacQueen GM, et al. Changes in cortical thickness across the lifespan in major depressive disorder. Psychiatry Res - Neuroimaging. 2013;214:204–211.

825. der Wee N van, Beekman ATF, Lamers F, Cuijpers P, der Meer K van, Ormel J, et al. Two-year course of depressive and anxiety disorders: Results from the Netherlands Study of Depression and Anxiety (NESDA). J Affect Disord. 2011;133:76–85.

826. van der Wee NJA, Eikelenboom M, Schruers K, van Oppen P, Aderka IM, van Balkom AJLM, et al. Age of onset in obsessive–compulsive disorder: admixture analysis with a large sample. Psychol Med. 2013;44:185–194.

827. Vandeleur C, Preisig M, Rodgers S, Ajdacic-Gross V, Rössler W, Kawohl W, et al. Comparing two basic subtypes in OCD across three large community samples: a pure compulsive versus a mixed obsessive–compulsive subtype. Eur Arch Psychiatry Clin Neurosci. 2015;265:719–734.

828. Vénisse J-L, Chéreau-Boudet I, Fatséas M, Romo L, Luquiens A, Magalon D, et al. Cognitive distortions and ADHD in pathological gambling: A national longitudinal case-control cohort study. J Behav Addict. 2016;5:649–657.

829. Verhaak P, Kok R, Voshaar RCO, Naarding P, Stek ML, de Waal MW, et al. The two-year course of late-life depression; results from the Netherlands study of depression in older persons. BMC Psychiatry. 2015;15:1–9.

830. Viviani B, Cecchi C, D'adamo D, Perone A, Vigliaturo D, Zucchi T, et al. Epidemiology of social phobia: a clinical approach. Eur Psychiatry. 2003;15:17–24.

831. Vyas NS, Hadjulis M, Vourdas A, Byrne P, Frangou S. The Maudsley early onset schizophrenia study: Predictors of psychosocial outcome at 4-year follow-up. Eur Child Adolesc Psychiatry. 2007;16:465–470.

832. Wang Y, Meng C, Wang W, Song M, Gao X, Moira S, et al. Influence of Brain-Derived Neurotrophic Factor Genetic Polymorphisms on the Ages of Onset for Heroin Dependence in a Chinese Population. Genet Test Mol Biomarkers. 2012;16:1044–1050.

833. Welham JL, Thomis R, McGrath JJ. Age-at-first-registration and heterogeneity in affective psychoses. Aust N Z J Psychiatry. 2003;37:66–69.

834. Wessely S. The Camberwell Study of Crime and Schizophrenia. Soc Psychiatry Psychiatr Epidemiol. 1998;33 Suppl 1:S24—8.

835. Wichow Icz HM, Cis Zew S K I S, Żu K K, Rybak-Korneluk A, Wichowicz HM. Hollow mask illusion – is it really a test for schizophrenia? Stanisław Kryzan Psychiatric Hospital in Starogard Gdanski. Psychiatr Pol. 2016;50:741–745.

836. Wilhelmsen KC. NIH Public Access. 2011;35:102–110.

837. Wilson S, Hicks BM, Foster KT, McGue M, Iacono WG. Age of onset and course of major depressive disorder: Associations with psychosocial functioning outcomes in adulthood. Psychol Med. 2015;45:505–514.

838. Wölfl M, Kuball J, Eyrich M, Schlegel PG, D P. NIH Public Access. Cytometry. 2009;73:1043–1049.

839. Woo YS, Shim IH, Wang HR, Song HR, Jun TY, Bahk WM. A diagnosis of bipolar spectrum disorder predicts diagnostic conversion from unipolar depression to bipolar disorder: A 5-year retrospective study. J Affect Disord. 2014;174:83–88.

840. Woodside DB, Garfinkel PE. Age of onset in eating disorders. Int J Eat Disord. 1992. 1992. https://doi.org/10.1002/1098-108X(199207)12:1<31::AID-EAT2260120105>3.0.CO;2-S.

841. Wu L-T, Howard MO, Pilowsky DJ. Substance use disorders among inhalant users: results from the National Epidemiologic Survey on alcohol and related conditions. Addict Behav. 2008;33:968—973.

842. Yao HJ, Zhang XY, Zhang CX, Yan XM, Li CB, Kosten TR, et al. The study of BDNF Val66Met polymorphism in Chinese schizophrenic patients. Prog Neuro-Psychopharmacology Biol Psychiatry. 2010;34:930–933.

843. L. Z, D. S, R. C, P. S, L. J, S. M, et al. Mixed, melancholic, and anxious features in depression: A cross-sectional study of sociodemographic and clinical correlates. Ann Clin Psychiatry. 2014;26:243–253.

844. Zhang X, Liu J, Cui J, Liu C. Study of symptom dimensions and clinical characteristics in Chinese patients with OCD. J Affect Disord. 2013;151:868–874.

845. Zhang L, Tang Y-L, Wang C-Y, Li X-B, Liu J-T, Zhu X-Z. Childhood trauma associates with clinical features of bipolar disorder in a sample of Chinese patients. J Affect Disord. 2014;168:58–63.

846. Zhong M, Cai L, Zhu X, Zhang X, Fan J, Lei H. Age at symptom onset is not associated with reduced action cancelation in adults with obsessive-compulsive disorder. Psychiatry Res. 2017;252:180–184.

847. Zhu T, D M, Luca V De, Ph D, Gallaugher LA, Sc HB, et al. Admixture analysis of age at onset in major depressive disorder. Gen Hosp Psychiatry. 2012;34:686–691.

848. Gilman S, Kawachi I, Fitzmaurice G, Buka S. Socioeconomic status in childhood and the lifetime risk of major depression. Int J Epidemiol. 2002;31:359–367.

849. Häfner H, Maurer K, Löffler W, An Der Heiden W, Munk-Jørgensen P, Hambrecht M, et al. The ABC schizophrenia study: A preliminary overview of the results. Soc Psychiatry Psychiatr Epidemiol. 1998;33:380–386.

850. Tian H, Xu G, Schoevers RA, Yin H, Yang G, Wardenaar KJ. The prevalence, age-of-onset and the correlates of DSM-IV psychiatric disorders in the Tianjin Mental Health Survey (TJMHS). Psychol Med. 2017;48:473–487.

851. Wittchen HU, Essau CA, von Zerssen D, Krieg JC, Zaudig M. Lifetime and six-month prevalence of mental disorders in the Munich follow-up study. Eur Arch Psychiatry Clin Neurosci. 1992;241:247–258.

852. Alegría M, Mulvaney-Day N, Torres M, Polo A, Cao Z, Canino G. Prevalence of psychiatric disorders across Latino subgroups in the United States. Am J Public Health. 2007;97:68–75.

853. Andrade L, Caraveo-Anduaga JJ, Berglund P, Bijl R V., De Graaf R, Vollebergh W, et al. The epidemiology of major depressive episodes: Results from the International Consortium of Psychiatric Epidemiology (ICPE) Surveys. Int J Methods Psychiatr Res. 2003;12:3–21.

854. Atladottir HO, Gyllenberg D, Langridge A, Sandin S, Hansen SN, Leonard H, et al. The increasing prevalence of reported diagnoses of childhood psychiatric disorders: a descriptive multinational comparison. Eur Child Adolesc Psychiatry. 2014;24:173–183.

855. Auerbach RP, Alonso J, Axinn WG, Cuijpers P, Ebert DD, Green JG, et al. HHS Public Access. 2017;46:2955–2970.

856. Beesdo K, Bittner A, Pine DS, Stein MB, Höfler M, Lieb R, et al. Incidence of social anxiety disorder and the consistent risk for secondary depression in the first three decades of life. Arch Gen Psychiatry. 2007;64:903—912.

857. Bland RC, Orn H, Newman SC. Lifetime Prevalence of Psychiatric Disorders in Edmonton. Acta Psychiatr Scand. 1988;77:24–32.

858. Borges G, Benjet C, Orozco R, Medina-Mora M-E, Menendez D. Alcohol, cannabis and other drugs and subsequent suicide ideation and attempt among young Mexicans. J Psychiatr Res. 2017;91:74—82.

859. Bourdon KH, Boyd JH, Rae DS, Burns BJ, Thompson JW, Locke BZ. Gender differences in phobias: Results of the ECA community survey. J Anxiety Disord. 1988. 1988. https://doi.org/10.1016/0887-6185(88)90004-7.

860. Brennan PA, Mednick SA, Hodgins S. Major mental disorders and criminal violence in a Danish birth cohort. Arch Gen Psychiatry. 2000;57:494–500.

861. Carraro A, Quadros L de CM de, Ribeiro FG, Motta JV dos S, Gigante DP, Horta BL, et al. Social Mobility and Mental Disorders at 30 Years of Age in Participants of the 1982 Cohort, Pelotas, Rio Grande Do Sul – RS. PLoS One. 2015;10:e0136886.

862. Caye A, Rocha TB-M, Anselmi L, Murray J, Menezes AMB, Barros FC, et al. Attention-Deficit/Hyperactivity Disorder Trajectories From Childhood to Young Adulthood: Evidence From a Birth Cohort Supporting a Late-Onset Syndrome. JAMA Psychiatry. 2016;73:705—712.

863. Colman I, Croudace TJ, Wadsworth MEJ, Jones PB. Factors associated with antidepressant, anxiolytic and hypnotic use over 17 years in a national cohort. J Affect Disord. 2008;110:234–240.

864. Colman I, Murray J, Abbott RA, Maughan B, Kuh D, Croudace TJ, et al. Outcomes of conduct problems in adolescence: 40 Year follow-up of national cohort. BMJ. 2009;338:208–211.

865. de Graaf R, ten Have M, Enns MW, Henriksen CA, Stein MB, Sareen J, et al. Common Mental Disorder Diagnosis and Need for Treatment are Not the Same: Findings from the NEMESIS Study. Adm Policy Ment Heal Ment Heal Serv Res. 2016;44:572–581.

866. Dickerson DL, Fisher DG, Reynolds GL, Baig S, Napper LE, Anglin MD. Substance use patterns among high-risk american indians/alaska natives in los angeles county. Am J Addict. 2012;21:445–452.

867. Eaton WW, Kramer M, Anthony JC, Dryman A, Shapiro S, Locke BZ. The incidence of specific DIS/DSM-III mental disorders: data from the NIMH Epidemiologic Catchment Area Program. Acta Psychiatr Scand. 1989;79:163–178.

868. Gerstenberg M, Hauser M, Al-Jadiri A, Sheridan EM, Kishimoto T, Borenstein Y, et al. Frequency and correlates of DSM-5 attenuated psychosis syndrome in a sample of adolescent inpatients with nonpsychotic psychiatric disorders. J Clin Psychiatry. 2015;76:e1449–e1458.

869. Grant BF. The relationship between ethanol intake and DSM-III-R alcohol dependence: results of a national survey. J Subst Abuse. 1993;5:257—267.

870. Grant BF. Age at smoking onset and its association with alcohol consumption and DSM-IV alcohol abuse and dependence: results from the National Longitudinal Alcohol Epidemiologic Survey. J Subst Abuse. 1998;10:59—73.

871. Grant BF. The impact of a family history of alcoholism on the relationship between age at onset of alcohol use and DSM-IV alcohol dependence: results from the National Longitudinal Alcohol Epidemiologic Survey. Alcohol Health Res World. 1998;22:144—147.

872. Harford TC, Yi H-Y, Grant BF. The five-year diagnostic utility of 'diagnostic orphans' for alcohol use disorders in a national sample of young adults. J Stud Alcohol Drugs. 2010;71:410—417.

873. Harvey SB, Wessely S, Kuh D, Hotopf M. The relationship between fatigue and psychiatric disorders: Evidence for the concept of neurasthenia. J Psychosom Res. 2009;66:445–454.

874. Harvey SB, Sellahewa DA, Wang M-J, Milligan-Saville J, Bryan BT, Henderson M, et al. The role of job strain in understanding midlife common mental disorder: a national birth cohort study. The Lancet Psychiatry. 2018;5:498–506.

875. Hatch SL, Mishra G, Hotopf M, Jones PB, Kuh D. Appraisals of stressors and common mental disorder from early to mid-adulthood in the 1946 British birth cohort. J Affect Disord. 2009;119:66–75.

876. Hatch SL, Harvey SB, Maughan B. A developmental-contextual approach to understanding mental health and well-being in early adulthood. Soc Sci Med. 2010;70:261–268.

877. Heimberg RG, Stein MB, Hiripi E, Kessler RC. Trends in the prevalence of social phobia in the United States: A synthetic cohort analysis of changes over four decades. Eur Psychiatry. 2000;15:29–37.

878. Helzer JE, Canino GJ, Yeh EK, Bland RC, Lee CK, Hwu HG, et al. Alcoholism--North America and Asia. A comparison of population surveys with the Diagnostic Interview Schedule. Arch Gen Psychiatry. 1990;47:313—319.

879. Hennig T, Jaya ES, Koglin U, Lincoln TM. Associations of attention-deficit/hyperactivity and other childhood disorders with psychotic experiences and disorders in adolescence. Eur Child Adolesc Psychiatry. 2017;26:421–431.

880. Isohanni M, Miettunen J, Koivumaa-Honkanen H-TJ, Räsänen P, Fenton W, Alaräisänen A. Suicide rate in schizophrenia in the Northern Finland 1966 Birth Cohort. Soc Psychiatry Psychiatr Epidemiol. 2009;44:1107–1110.

881. Kalaydjian A, Swendsen J, Chiu W-T, Dierker L, Degenhardt L, Glantz M, et al. Sociodemographic predictors of transitions across stages of alcohol use, disorders, and remission in the National Comorbidity Survey Replication. Compr Psychiatry. 2009;50:299—306.

882. Karno M, Leaf PJ, Florio LP, Weissman MM, Bruce ML, Tischler GL, et al. Affective disorders in five United States communities. Psychol Med. 2009;18:141.

883. Kessler RC, Stein MB, Berglund P. Social phobia subtypes in the National Comorbidity Survey. Am J Psychiatry. 1998;155:613—619.

884. Koenen KC, Ratanatharathorn A, Ng L, McLaughlin KA, Bromet EJ, Stein DJ, et al. Posttraumatic stress disorder in the World Mental Health Surveys. Psychol Med. 2017;47:2260–2274.

885. Kraus L, Bloomfield K, Augustin R, Reese A. Prevalence of alcohol use and the association between onset of use and alcohol-related problems in a general population sample in Germany. Addiction. 2000;95:1389–1401.

886. Kit-Wa Chan S, Shuk-Kuen Kwok V, Lai-Ming Hui C, Chang W-C, Yu-Hai Chen E, Wing-Yan Poon V, et al. Prevalence and predictors of medication non-adherence among Chinese patients with first-episode psychosis. Psychiatry Res. 2015;228:680–687.

887. Lee S, Ng KL, Kwok KPS, Tsang A. Prevalence and correlates of social fears in Hong Kong. J Anxiety Disord. 2009;23:327–332.

888. Lev-Ran S, Le Foll B, McKenzie K, George TP, Rehm J. Bipolar disorder and co-occurring cannabis use disorders: characteristics, co-morbidities and clinical correlates. Psychiatry Res. 2013;209:459—465.

889. Louie P, Wheaton B. Prevalence and Patterning of Mental Disorders Through Adolescence in 3

Cohorts of Black and White Americans. Am J Epidemiol. 2018;187:2332–2338.

890. Lukat J, Becker ES, Lavallee KL, van der Veld WM, Margraf J. Predictors of Incidence, Remission and Relapse of Axis I Mental Disorders in Young Women: A Transdiagnostic Approach. Clin Psychol Psychother. 2017;24:322–331.

891. Marshall M. Combining insights from epidemiological and ethnographic data to investigate substance use in Truk, Federated States of Micronesia. Br J Addict. 1990;85:1457—1468.

892. Moffitt TE, Caspi A, Newman DL, Silva PA, Stanton WR, Magdol L. Psychiatric disorder in a birth cohort of young adults: Prevalence, comorbidity, clinical significance, and new case incidence from ages 11 to 21. J Consult Clin Psychol. 2005;64:552–562.

893. Nagl M, Jacobi C, Paul M, Beesdo-Baum K, Höfler M, Lieb R, et al. Prevalence, incidence, and natural course of anorexia and bulimia nervosa among adolescents and young adults. Eur Child Adolesc Psychiatry. 2016;25:903–918.

894. Nierenberg AA, Akiskal HS, Angst J, Hirschfeld RM, Merikangas KR, Petukhova M, et al. Bipolar disorder with frequent mood episodes in the national comorbidity survey replication (NCS-R). Mol Psychiatry. 2010;15:1075–1087.

895. Nock MK, Kazdin AE, Hiripi E, Kessler RC. Lifetime prevalence, correlates, and persistence of oppositional defiant disorder: Results from the National Comorbidity Survey Replication. J Child Psychol Psychiatry Allied Discip. 2007;48:703–713.

896. Ouk Park H, Woo Kim C, Ouk Kim Y, Lee KH, Ho Chae C, Saeng Jung Y, et al. Anxiety symptoms and occupational stress among young Korean female manufacturing workers. Ann Occup Environ Med. 2015;27:1–9.

897. Paksarian D, Trabjerg BB, Merikangas KR, Mors O, Borglum AD, Hougaard DM, et al. The role of genetic liability in the association of urbanicity at birth and during upbringing with schizophrenia in Denmark. Psychol Med. 2018;48:305–314.

898. Patton GC, Coffey C, Romaniuk H, Mackinnon A, Carlin JB, Degenhardt L, et al. The prognosis of common mental disorders in adolescents: A 14-year prospective cohort study. Lancet. 2014;383:1404–1411.

899. Perkonigg A, Kessler RC, Storz S, Wittchen HU. Traumatic events and post-traumatic stress disorder in the community: Prevalence, risk factors and comorbidity. Acta Psychiatr Scand. 2000. 2000. https://doi.org/10.1034/j.1600-0447.2000.101001046.x.

900. Perkonigg A, Pfister H, Hofler M, Frohlich C, Zimmermann P, Lieb R, et al. Substance use and substance use disorders in a community sample of adolescents and young adults: incidence, age effects and patterns of use. Eur Addict Res. 2006;12:187—196.

901. Ravens-Sieberer U, Otto C, Kriston L, Rothenberger A, Döpfner M, Herpertz-Dahlmann B, et al. The longitudinal BELLA study: design, methods and first results on the course of mental health problems. Eur Child Adolesc Psychiatry. 2015;24:651–663.

902. Rees S, Steel Z, Creamer M, Teesson M, Bryant R, McFarlane AC, et al. Onset of common mental disorders and suicidal behavior following women's first exposure to gender based violence: A retrospective, population-based study. BMC Psychiatry. 2014;14:1–8.

903. Ruggeri M, Urbani A, Riolo R, Mazzoncini R, De Santi K, Tosato S, et al. The impact of cannabis use on age of onset and clinical characteristics in first-episode psychotic patients. Data from the Psychosis Incident Cohort Outcome Study (PICOS). J Psychiatr Res. 2013;47:438–444.

904. Sandanger I, Nygard JF, Ingebrigtsen G, Sørensen T, Dalgard OS. Prevalence, incidence and age at onset of psychiatric disorders in Norway. Soc Psychiatry Psychiatr Epidemiol. 1999;34:570–579.

905. Shetye SS. Prevalence and correlates of depression in elderly Chinese in Hong Kong. 2007. 2007.

906. Simon GE, VonKorff M. Reevaluation of secular trends in depression rates. Am J Epidemiol. 1992;135:1411—1422.

907. Stice E, Marti CN, Shaw H, Jaconis M. NIH Public Access. Eat Disord. 2010;118:587–597.

908. Sung M, Erkanli A, Angold A, Costello EJ. Effects of age at first substance use and psychiatric comorbidity on the development of substance use disorders. Drug Alcohol Depend. 2004;75:287—299.

909. Van Milligen BA, Lamers F, De Hoop GT, Smit JH, Penninx BWJH. Objective physical functioning in patients with depressive and/or anxiety disorders. J Affect Disord. 2011;131:193–199.

910. Vilalta-Franch J, Planas-Pujol X, López-Pousa S, Llinàs-Reglà J, Merino-Aguado J, Garre-Olmo J. Depression subtypes and 5-years risk of mortality in aged 70 years: a population-based cohort study. Int J Geriatr Psychiatry. 2012;27:67—75.

911. Wang PS, Berglund PA, Kessler RC. Patterns and correlates of contacting clergy for mental disorders in the United States. Health Serv Res. 2003;38:647–673.

912. Weissman MM, Leaf PJ, Holzer CE, Myers JK, Tischler GL. The epidemiology of depression. An update on sex differences in rates. J Affect Disord. 1984. 1984. https://doi.org/10.1016/0165-0327(84)90039-9.

913. White A, Castle IJP, Chen CM, Shirley M, Roach D, Hingson R. Converging Patterns of Alcohol Use and Related Outcomes Among Females and Males in the United States, 2002 to 2012. Alcohol Clin Exp Res. 2015;39:1712–1726.

914. WITTCHEN H-U, BECKER E, LIEB R, KRAUSE P. Prevalence, incidence and stability of premenstrual dysphoric disorder in the community. Psychol Med. 2008;32:119–132.

915. Alvarado GF. Epidemiology of Cocaine Use and Dependence. Michigan State University, Epidemiology; 2012.

916. Angermeyer MC, Kühn L. Gender differences in age at onset of schizophrenia. An overview. Eur Arch Psychiatry Neurol Sci. 1988;237:351—364.

917. Bucholz KK. Nosology and epidemiology of addictive disorders and their comorbidity. Psychiatr Clin North Am. 1999;22:221–240.

918. Burcusa SL, Iacono WG. Risk for recurrence in depression. Clin Psychol Rev. 2007;27:959—985.

919. Burns JK, Jhazbhay K, Esterhuizen T, Emsley R. Exposure to trauma and the clinical presentation of first-episode psychosis in South Africa. J Psychiatr Res. 2011;45:179–184.

920. Clarizio HF. Continuity in childhood depression. Adolescence. 1989;24:253—267.

921. De Lijster JM, Dierckx B, Utens EMWJ, Verhulst FC, Zieldorff C, Dieleman GC, et al. The age of onset of anxiety disorders: A meta-analysis. Can J Psychiatry. 2017;62:237–246.

922. Flor-Henry P. Schizophrenia: sex differences. Can J Psychiatry. 1985;30:319—322.

923. Gold DD. Late age of Onset Schizophrenia: present but unaccounted for. Compr Psychiatry. 1984;25:225—237.

924. Hardy K V, Noordsy DL, Ballon JS, McGovern MP, Salomon C, Wiltsey Stirman S. Impact of age of onset of psychosis and engagement in higher education on duration of untreated psychosis. J Ment Heal. 2018;27:257–262.

925. Kessler RC, Bromet EJ. The epidemiology of depression across cultures. Annu Rev Public Heal. 2013;34:119–138.

926. Loeber R, Lahey BB, Thomas C. Diagnostic conundrum of oppositional defiant disorder and conduct disorder. J Abnorm Psychol. 1991;100:379—390.

927. Spinhoven P, van Balkom AJLM, van Oppen P, Penninx BW, Anholt GE, Tibi L, et al. Examining determinants of early and late age at onset in panic disorder: An admixture analysis. J Psychiatr Res. 2013;47:1870–1875.

928. Winter EC, Bienvenu OJ. Temperament and anxiety disorders. Handb. child Adolesc. anxiety Disord., Springer; 2011. p. 203–212.

929. Zoccolillo M, Price R, Ji THC, Hwu H-G. Antisocial personality disorder: Comparisons of prevalence, symptoms, and correlates in four countries. Hist. Geogr. Influ. Psychopathol., 1999.

930. Welch KL. Do angels fall? An analysis of late onset offending and substance use in adults based on findings from the National Youth Survey Family Study. Diss Abstr Int Sect A Humanit Soc Sci. 2010. 2010.
